# Blood matters: the hematological signatures of Coronavirus infection

**DOI:** 10.1038/s41419-024-07247-8

**Published:** 2024-11-28

**Authors:** Ayelen Toro, Ana P. Arévalo, Marianoel Pereira-Gómez, Agustina Sabater, Eric A. Zizzi, Paula Perbolianachis, Gaston Pascual, Sofia Lage-Vickers, Jorge L. Pórfido, Ines Achinelli, Rocio Seniuk, Juan Bizzotto, Pablo Sanchis, Alvaro Olivera, Alejandro Leyva, Pilar Moreno, Alicia Costábile, Alvaro Fajardo, Federico Carrión, Martín Fló, Natalia Olivero-Deibe, Fernando Rodriguez, Nicolas Nin, Nicolas Anselmino, Estefania Labanca, Elba Vazquez, Javier Cotignola, Daniel F. Alonso, Maria P. Valacco, Marcelo Marti, Francesco Gentile, Artem Cherkasov, Martina Crispo, Gonzalo Moratorio, Geraldine Gueron

**Affiliations:** 1 Departamento de Química Biológica, Facultad de Ciencias Exactas y Naturales, Universidad de Buenos Aires, Buenos Aires, Argentina; 2 Instituto de Química Biológica de la Facultad de Ciencias Exactas y Naturales (IQUIBICEN), CONICET-Universidad de Buenos Aires, Buenos Aires, Argentina; 3 Unidad de Biotecnología en Animales de Laboratorio, Institut Pasteur de Montevideo, Montevideo, Uruguay; 4 Laboratorio de Virología Molecular, Facultad de Ciencias, Universidad de la República, Montevideo, Uruguay; 5 Laboratorio de Evolución Experimental de Virus, Institut Pasteur de Montevideo, Montevideo, Uruguay; 6 Instituto de Tecnología (INTEC), Universidad Argentina de la Empresa (UADE), Buenos Aires, Argentina; 7 PolitoBIOMed Lab, Department of Mechanical and Aerospace Engineering, Politecnico di Torino, Turin, Italy; 8 Department of Chemistry and Biomolecular Sciences, University of Ottawa, Ottawa, ON Canada; 9 Laboratorio de Alta Resolución, Departamento de Desarrollo Tecnológico, Centro Universitario Regional Este (CURE), Universidad de la República, Rocha, Uruguay; 10 Instituto de Investigaciones Biológicas Clemente Estable, Montevideo, Uruguay; 11 Unidad de Bioquímica y Proteómica Analíticas, Institut Pasteur de Montevideo, Montevideo, Uruguay; 12 Centro de Innovación en Vigilancia Epidemiológica, Institut Pasteur de Montevideo, Montevideo, Uruguay; 13 Sección Bioquímica, Facultad de Ciencias, Universidad de la República, Montevideo, Uruguay; 14 Laboratorio de Inmunovirología, Institut Pasteur de Montevideo, Montevideo, Uruguay; 15 Departamento de Inmunobiología, Facultad de Medicina, Universidad de la República, Montevideo, Uruguay; 16 Unidad de Cuidados Intensivos, Hospital Español “Juan José Crottoggini”, Administración de Servicios de Salud del Estado, Montevideo, Uruguay; 17 Department of Genitourinary Medical Oncology and The David H. Koch Center for Applied Research of Genitourinary Cancers, The University of Texas MD Anderson Cancer Center, Houston, TX USA; 18 Centro de Oncología Molecular y Traslacional y Plataforma de Servicios Biotecnológicos, Departamento de Ciencia y Tecnología, Universidad Nacional de Quilmes, Bernal, Argentina; 19 Ottawa Institute of Systems Biology, Ottawa, ON Canada; 20 Vancouver Prostate Centre, University of British Columbia, Vancouver, BC Canada

**Keywords:** Infection, Viral infection

## Abstract

Recent developments have broadened our perception of SARS-CoV-2, indicating its capability to affect the body systemically beyond its initial recognition as a mere respiratory pathogen. However, the pathways of its widespread are not well understood. Employing a dual-modality approach, we integrated findings from a Murine Hepatitis Virus (MHV) infection model with corroborative clinical data to investigate the pervasive reach of Coronaviruses. The novel presence of viral particles within red blood cells (RBCs) was demonstrated via high-resolution transmission electron microscopy, with computational modeling elucidating a potential heme-mediated viral entry mechanism via Spike protein affinity. Our data affirm viral localization in RBCs, suggesting heme moieties as facilitators for cellular invasion. Exacerbation of MHV pathology upon hemin administration, contrasted with chloroquine-mediated amelioration, underscoring a heme-centric pathway in disease progression. These observations extend the paradigm of Coronavirus pathogenicity to include hemoprotein interactions. This study casts new light on the systemic invasion capabilities of Coronaviruses, linking RBC hemoproteins with viral virulence. The modulation of disease severity through heme-interacting agents heralds a promising avenue for COVID-19 therapeutics. Our findings propose a paradigm shift in the treatment approach, leveraging the virus-heme interplay as a strategic hinge for intervention.

## Introduction

The SARS-CoV-2 virus, while predominantly known for respiratory symptoms, also exerts systemic effects, extending to the heart, kidneys, and even the brain [1]. The resultant condition known as ‘long COVID’ presents a multisystemic syndrome affecting both adults and children, with a significant proportion—between 50 and 70%—experiencing persistent or emergent symptoms months following the initial infection [2, 3].

Long COVID symptoms have been linked to sustained hematological abnormalities observed post-recovery [4]. Notably, this extends to hematological biomarkers such as elevated ferritin, decreased hemoglobin (HGB), and increased erythrocyte sedimentation rate, alongside observable phenotypic alterations in blood cells persisting for months post-infection [4]. Thus, it is evident that persistent post-infection symptomatology requires a more accurate and extensive focus on the hematological manifestations. Moreover, SARS-CoV-2 infection has been linked to hemolytic complications, including thrombocytopenia and anemia [5]. Further, the binding of heme to viral and host proteins, such as the 7a protein, Spike (S), and ACE2, proposes a role for red blood cells (RBCs) in the virus’s systemic spread [6–9].

In this study, we explore the multi-organ impact of Coronavirus infection and the involvement of RBCs in disease propagation. We utilize the murine hepatitis virus (MHV-A59), a type 2 RNA Coronavirus analogous to SARS-CoV-2, as a model to gain mechanistic insights that may inform therapeutic strategies for COVID-19 [10]. Our findings present a composite picture of multi-organ disease and hematological dysregulation, enhancing our understanding of the progression of SARS-CoV-2 infection and the significance of RBCs in harboring infectious particles in COVID-19 patients.

## Methods

### Experimental model and study participant details

#### Human samples

Nasopharyngeal swabs and serum samples of ten COVID-19 patients, and tissues from human autopsies performed on a total of eight COVID-19 deceased patients were collected from Hospital Español (Administración de los Servicios de Salud del Estado, Uruguay). All patients were confirmed for SARS-CoV-2 infection through RT-qPCR analysis performed on nasopharyngeal swabs. Viral RNA was extracted using the QIAamp Viral RNA mini kit (QIAgen) and the qualitative and quantitative detection of SARS-CoV-2 was performed using the kit “COVID-19 RT-PCR Real TM Fast-HEX/Cy5 Kit” (ATGen, Institut Pasteur de Montevideo, Universidad de la República), according to the manufacturer’s protocols. Viral load was estimated using a calibration curve as previously reported [11]. Patient demographics are summarized in Supplementary Table S1.

All procedures performed in studies involving human subjects were in accordance with the ethical standards of the institutional and/or national research committee and with the 1964 Helsinki Declaration and its later amendments or comparable ethical standards. Documented approval was obtained from the Ethics Committee of Hospital Español (Administración de los Servicios de Salud del Estado, Uruguay).

#### Virus and cells

MHV-A59 (ATCC VR-764) viruses were cultured in our laboratory conditions by performing 10 serial passages in murine L929 cells (SIGMA). This stock was stored at $-$80 °C until further use. Cells were maintained in DMEM medium (Gibco) supplemented with 10% vol/vol FBS (Fetal Bovine Serum, Gibco), 1% vol/vol penicillin-streptomycin and incubated at 37 °C and 5% CO_2_.

#### In vivo experiments

A total of 38 BALB/cJ female mice (8–10 weeks old) were randomly distributed into five groups according to the experiment: (a) control mock-infected (MOCK, *n* = 9) were injected with 100 µL of vehicle (PBS), (b) MHV-infected (MHV + PBS, *n* = 11), (c) MHV-infected treated with hemin (#16009-13-5, SIGMA) (MHV + H, *n* = 6; 1 dose of 10 mg/kg on day 0, *i.p*.), d) MHV-infected treated with chloroquine (CQ) (#6628, SIGMA), (MHV + CQ, *n* = 6; 3 doses of 30 mg/kg on days -2, -1 and 1, *i.p*.), and e) MHV-infected treated with both hemin and CQ (MHV + H + CQ, *n* = 6). H and CQ doses were selected considering our previous experience [12] and supporting literature [13–15]. The sample size was determined by our previous experience using this animal model [16]. Experiments were conducted in two independent replicates. Mice were bred at the animal facility of the Laboratory Animals Biotechnology Unit of Institut Pasteur de Montevideo, infected with MHV-A59 (ATCC VR-764), and blood and tissue samples were collected after euthanasia as previously described in [17]. All the experimental protocols were approved by the institutional *Comisión de Ética en el Uso de Animales* (protocol #006-22) and were performed according to national law #18.611 and relevant international laboratory animal welfare guidelines and regulations. No blinding was done.

### Method details

#### Blood biochemistry profile

Individual whole blood (100 μL) was collected in 20 U/mL of heparin (Heparin Sodium salt, SIGMA) and analyzed for liver and kidney profile using the Pointcare V2 automatic device (Tianjin MNCHIP Technologies Co) at the beginning (pre-infection determination) and at the end of the experiment (post-infection determination). Analyzed parameters included total protein (TP), albumin (ALB), globulin (GLO), alanine aminotransferase (ALT), aspartate aminotransferase (AST), blood urea nitrogen (BUN), and glucose (GLU). ALT detection limit corresponds to 1500 (U/L) and AST, to 1600 (U/L).

For hematological analysis, aliquots of 20 μL of blood were collected into 0.5 mL microtubes containing EDTA potassium salts (W anticoagulant, Wiener lab) in a ratio of 1:10 (EDTA: blood) before and after infection. All measurements were conducted within four hours after collection. RBC count, HGB, hematocrit (HCT), White Blood Cells (WBC) and platelets (PLT) counts, and lymphocyte, neutrophil, and monocyte percentages were evaluated using the auto hematology analyzer BC-5000Vet (Mindray Medical International Ltd). Neutrophil-to-lymphocyte ratio (NLR) and platelet-to-lymphocyte ratio (PLR) were calculated using absolute values.

#### Organs’ RNA abundance assessment

Different organ samples were retrieved, and excess blood was removed by washing twice with PBS. Total RNA was isolated with Quick-Zol (Kalium technologies) according to the manufacturer’s protocol. cDNAs were synthesized with TransScript One-Step gDNA Removal and cDNA Synthesis SuperMix (Transgen Biotech) using random primers. Taq DNA Polymerase (Invitrogen) was used for real-time PCR amplification in a QuantStudio 3 Real-Time PCR System (Thermo Fisher Scientific). Quantification of RNA was performed as previously described [17].

#### Median tissue culture infectious doses

A total of 5 × 10^4^ L929 cells (SIGMA) were seeded in 96-well plates. Tenfold serial dilutions of supernatants from MHV-infected mice organs were prepared in serum-free DMEM media (Gibco). Infections were performed in twelve replicates. After five days, living cell monolayers were fixed and stained with crystal violet 0.2% vol/vol in formaldehyde 10% vol/vol. Median Tissue Culture Infectious Doses (TCID_50_) values obtained were normalized to the weight of the organ sampled and expressed as TCID_50_/g of tissue.

#### Blood fractionation

Blood with 10% vol/vol EDTA was initially centrifuged at 3000 × *g* for 30 min at 4 °C to separate RBC from plasma. 100 μL of PBS 1× were added to each fraction and split into two aliquots. One aliquot of each fraction was used to perform TRIzol RNA extraction for viral genome equivalents determination and the other aliquot was used to determine viable viral particles by plaque assay.

#### Determination of viral genome equivalents in blood fractions

RNA was extracted using TRIzol reagent (Invitrogen) according to the manufacturer’s protocol. The extracted RNA was used for MHV-A59 detection using a specific RT-qPCR protocol with a Taqman probe (ACAAGCTCAGGCACCTCCTGTACAA) labeled at the 5′-end with FAM (10 mM). The following primers were used for viral load determination: Nsp2 forward: TGGATGGCTTTGCTACCAG, and Nsp2 reverse: CCAGACAAGATAGAAACCGAC. All RT-qPCRs were performed in duplicate. Thermal cycling was run on a QuantStudio™ seven (Applied Biosystems). The standard curve was constructed as depicted in Supplementary Methods.

#### Plaque assays

L929 cells were seeded in 12-well plates and supernatants from MHV-infected mice blood fractions (plasma or RBC-enriched) were serially ten-fold diluted in DMEM serum-free medium (Gibco). After 1 h of viral adsorption, monolayers were topped with a semisolid overlay of DMEM medium supplemented with 2% FBS and 1% wt/vol agarose. Forty-eight hours post infection, cells were fixed with formaldehyde 10% vol/vol and stained with crystal violet 0.2% vol/vol.

#### Histological analysis

A detailed description of sample processing, staining, and examination is described in the Supplementary Methods section.

#### Transmission electron microscopy

HR-TEM analysis was performed to identify viral particles in the RBC-enriched fraction from MHV-infected mice at the High Resolution Laboratory of CURE Technological Development Department, Rocha, University of the Republic (Uruguay), as described in Supplementary Methods.

#### Proteomic analyses

A detailed description of the sample collection, protein extraction, LC ESI-MS/MS, and data analysis is reported in the Supplementary Methods section.

#### In silico interaction prediction

A detailed description of the computational methods used to build and refine the *in-silico* model of the MHV S protein, to perform the docking of heme, and to carry out molecular dynamics’ simulations of the MHV S-heme complex is reported in the Supplementary Methods section.

#### Validation of Spike–heme interaction

A detailed description of SARS-CoV-2 recombinant Spike protein purification, hemin binding assay and data analysis is reported in the Supplementary Methods section.

### Quantification and statistical analysis

Paired student’s *t*-test was performed to determine statistical differences between pre- and post-infection measurements in mice. ANOVA followed by Tukey test was performed to determine statistical differences between conditions. Statistical significance was set at *p* < 0.05.

## Results

### Coronavirus multi-organ tropism in COVID-19 patients and MHV-infected mice

**Fig. 1:**
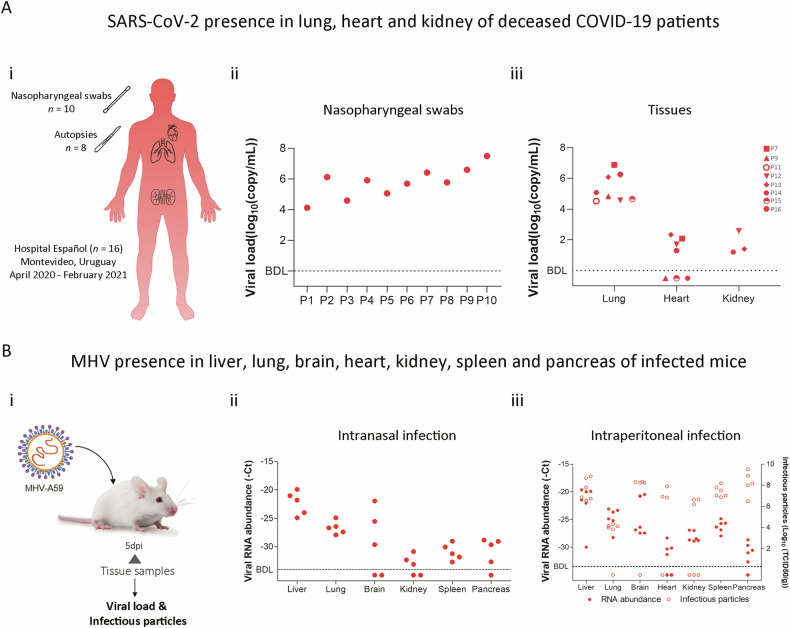
SARS-CoV-2 and MHV RNA were detected in different tissues. **A** (**i**) Schematic representation of sample acquisition from COVID-19 patients from Hospital Español (Administración de los Servicios de Salud del Estado, Uruguay). Nasopharyngeal swabs (*n* = 10) and lung, heart, and kidney samples from human autopsies (*n* = 8) were collected for viral load by RT-qPCR analysis. (**ii**) Viral load (log_10_(copy/mL) of nasopharyngeal swabs and samples from COVID-19 patients (*P*). (**iii**) Viral load (log_10_(copy/mL) of different organ samples (lung, heart, and kidney) obtained from patient autopsies. **B** (**i**) Experimental design of murine Coronavirus infection. BALB/cJ mice were infected with MHV-A59 by intranasal administration (4000 PFU) or by *i.p*. injection (6000 PFU). Five days post-infection (dpi), the liver, lung, brain, heart, kidney, spleen, and pancreas were dissected for RT-qPCR analyzes. (**ii**) Viral RNA abundance (−Ct) measured by RT-qPCR in liver, lung, brain, kidney, spleen, and pancreas samples from mice (*n* = 5) infected by intranasal administration. (**iii**) Viral RNA abundance (−Ct) measured by RT-qPCR (filled circles) and viral titer (log_10_(TCID50/g) (open circles) in liver, lung, brain, heart, kidney, spleen, and pancreas samples from mice (*n* = 6) infected by intraperitoneal administration. BDL below detection limit.

In this cohort study (*n* = 16), we confirmed SARS-CoV-2 infection assessing nasopharyngeal swabs (*n* = 10) (Fig. 1Ai, Aii, Supplementary Fig. S1 and Table S1). Further, post-mortem examinations were conducted on individuals who succumbed to the disease (*n* = 8), which revealed high viral loads in all pulmonary specimens, while viral RNA was also detected in 57.14% of cardiac tissues (4/7) and universally in renal tissues, albeit at lower viral loads (Fig. 1Aiii). To extend these findings, we leveraged a murine pre-clinical model infected with MHV-A59 through intranasal or intraperitoneal routes. A comprehensive organ analysis was conducted five days post-infection (dpi) (Fig. 1Bi). revealing viral RNA in liver, lung, brain, heart, kidney, spleen, and pancreas regardless of the infection route (Fig. 1Bii,1Biii), paralleling the human viral dissemination in various organs. Moreover, we detected infectious viral particles across all the organs examined (Fig. 1Biii), substantiating the hypothesis of a multi-organ tropism in SARS-CoV-2 infection, reflecting the pathological observations in human COVID-19 and underscoring the systemic nature of this viral pathogen.

### SARS-CoV-2 and MHV shared pathophysiological consequences

**Fig. 2:**
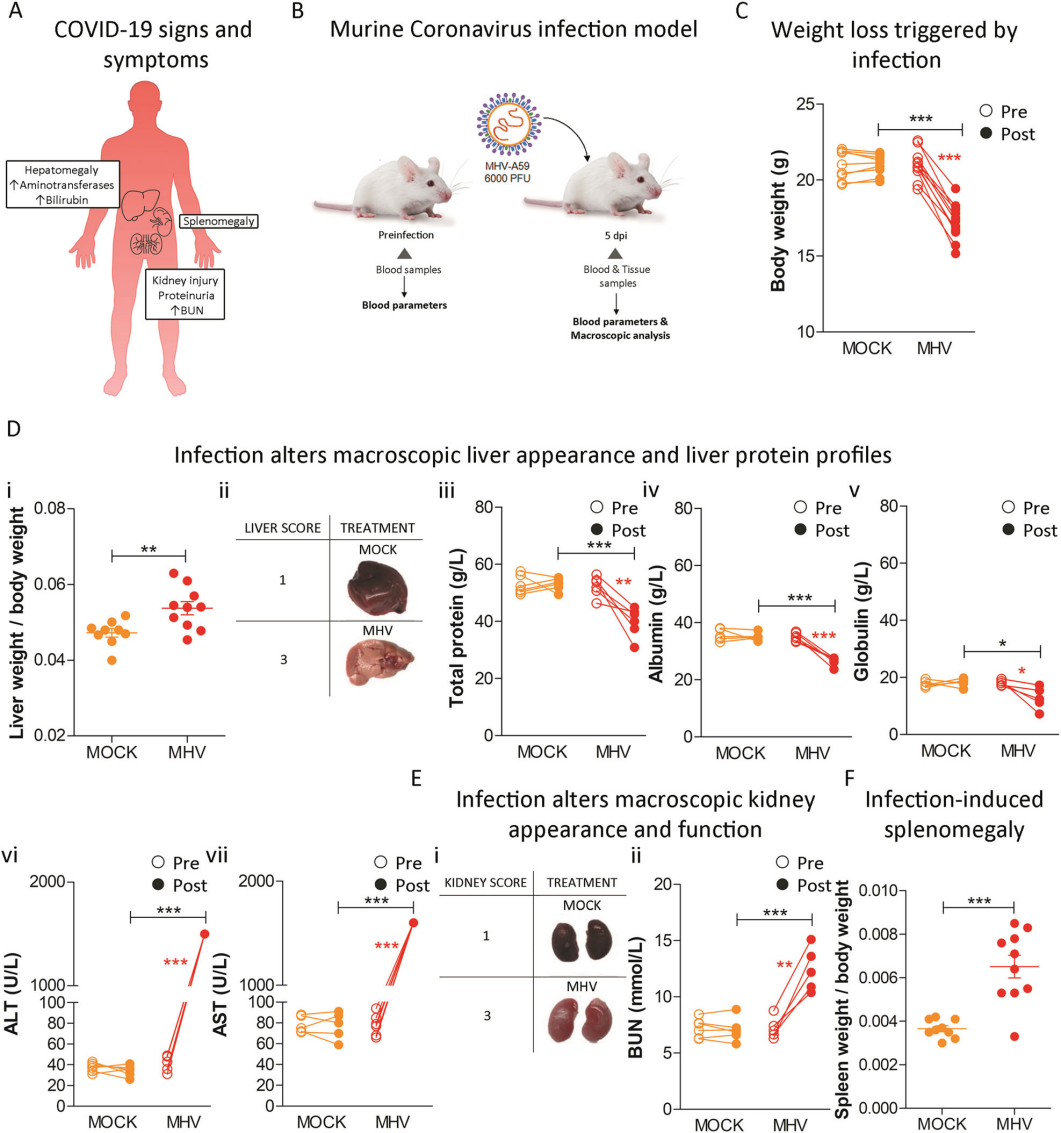
Comparative pathophysiology of SARS-CoV-2 and MHV infections. **A** COVID-19 multiorgan manifestations (adapted from [18]) **B** Experimental design of murine Coronavirus infection. BALB/cJ mice were infected with 6000 PFU of MHV-A59 by intraperitoneal injection. Five days post-infection (dpi), the liver and kidney were dissected for macroscopic analyzes. Blood samples were also taken pre- and post-infection for determining blood parameters. **C** Body weight pre- and post-infection of MHV-infected (MHV) and uninfected (MOCK) mice. **D** Liver weight (**i**), macroscopic appearance and pathological scores (grades 1 to 3, where 1 is no damage and 3 is the most damaged) (**ii**) at necropsy five dpi in MHV-infected (MHV), and uninfected (MOCK) mice. Total protein (g/L) (**iii**) albumin (g/L) (**iv**) globulin (g/L) (**v**) alanine transaminase (ALT) (g/L) (**vi**) and aspartate aminotransferase (AST) (g/L) (**vii**) levels measured in the blood of MHV-infected (MHV), and uninfected (MOCK) mice, pre- and post-infection. **E** Kidney macroscopic appearance and pathological scores (grades 1 to 3, where 1 is no damage and 3 is the most damaged) (**i**) at necropsy five dpi in MHV-infected (MHV), and uninfected mice (PBS). Blood urea nitrogen (BUN) (mmol/L) (**ii**) levels measured in the blood of MHV-infected (MHV), and uninfected mice (MOCK), pre- and post-infection. **F** Spleen weight at necropsy five dpi in MHV-infected (MHV), and uninfected (MOCK) mice. Results are shown as the mean ±SD. Paired student’s *t*-test was performed to determine statistical differences between pre- and post-infection. Unpaired student’s *t*-test was performed to determine statistical differences between MHV and mock. Statistical significance was set at *p* < 0.05. * *p* < 0.05, ** *p* < 0.01, *** *p* < 0.001.

The pathophysiological parallels between COVID-19 and MHV infection are increasingly evident. SARS-CoV-2 infection is implicated in numerous extrapulmonary manifestations, with alterations in liver and kidney functions frequently noted in patients with COVID-19 [18] (Fig. 2A). Utilizing a preclinical MHV infection model, we evaluated the impact of this Coronavirus on a range of biochemical parameters by analyzing paired blood pre- and post-infection samples (Fig. 2B). MHV infection precipitated a marked reduction in body weight (*p* < 0.001; Fig. 2C), and hepatomegaly was observed (*p* < 0.01; Fig. 2Di), with an associated pathological score increase (Fig. 2Dii). Moreover, liver from infected mice showed multifocal to coalescent necrosis affecting the entire parenchyma, mild inflammatory reactions, and severe cytoplasmic vacuolation (Supplementary Fig. S2). Pertinently, liver function parameters including TP (Fig. 2Diii), ALB (Fig. 2Div), and GLO (Fig. 2Dv) were diminished following MHV infection (*p* < 0.01; *p* < 0.001; and *p* < 0.05, respectively). Conversely, enzymes indicative of liver damage, Alanine Transaminase (ALT) (Fig. 2Dvi), and AST (Fig. 2Dvii), were elevated (*p* < 0.001 for both comparisons). Kidney mass remained unchanged post-infection (Supplementary Fig. S3Aiv). Nevertheless, kidneys from MHV-infected mice exhibited notable pathological alterations, garnering the highest severity score (Fig. 2Ei), accompanied by a significant elevation in BUN levels post-infection (*p* < 0.01; Fig. 2Eii). Further, histological analysis of kidneys from infected mice showed multifocal areas of tubular necrosis in the collecting tubules with mild lymphoplasmacytic inflammatory reaction (Supplementary Fig. S2). Additionally, splenomegaly was observed upon MHV infection (*p* < 0.001; Fig. 2F) and lymphoid hyperplasia, congestion, and abundant presence of macrophages and multinucleated inflammatory cells were observed in the spleen of MHV-infected mice (Supplementary Fig. S2). Notably, a comparative reduction in cardiac mass (*p* < 0.05; Supplementary Fig. S3Aiii) with diffuse myocarditis (Supplementary Fig. S2) was recorded in MHV-infected mice compared to their uninfected counterparts. No significant changes were observed in the weight or macroscopic appearance of the lungs and brain of MHV-infected but histological analysis showcased mild edema and interstitial pneumonia, and mild gliosis, respectively (Supplementary Figs. S2, S3Ai, S3Aii, S3B, S3C, lines 1 and 2). Pancreases of infected mice did not exhibit significant variations in weight or phenotypic alterations, and their macroscopic appearance and pathological scores remained unchanged (Supplementary Figs. S3Av, S3B, S3C, lines 1 and 2). These findings mirror multiple aspects of the multi-organ impact observed in human COVID-19.

### Systemic presence of SARS-CoV-2 and MHV in blood specimens

**Fig. 3:**
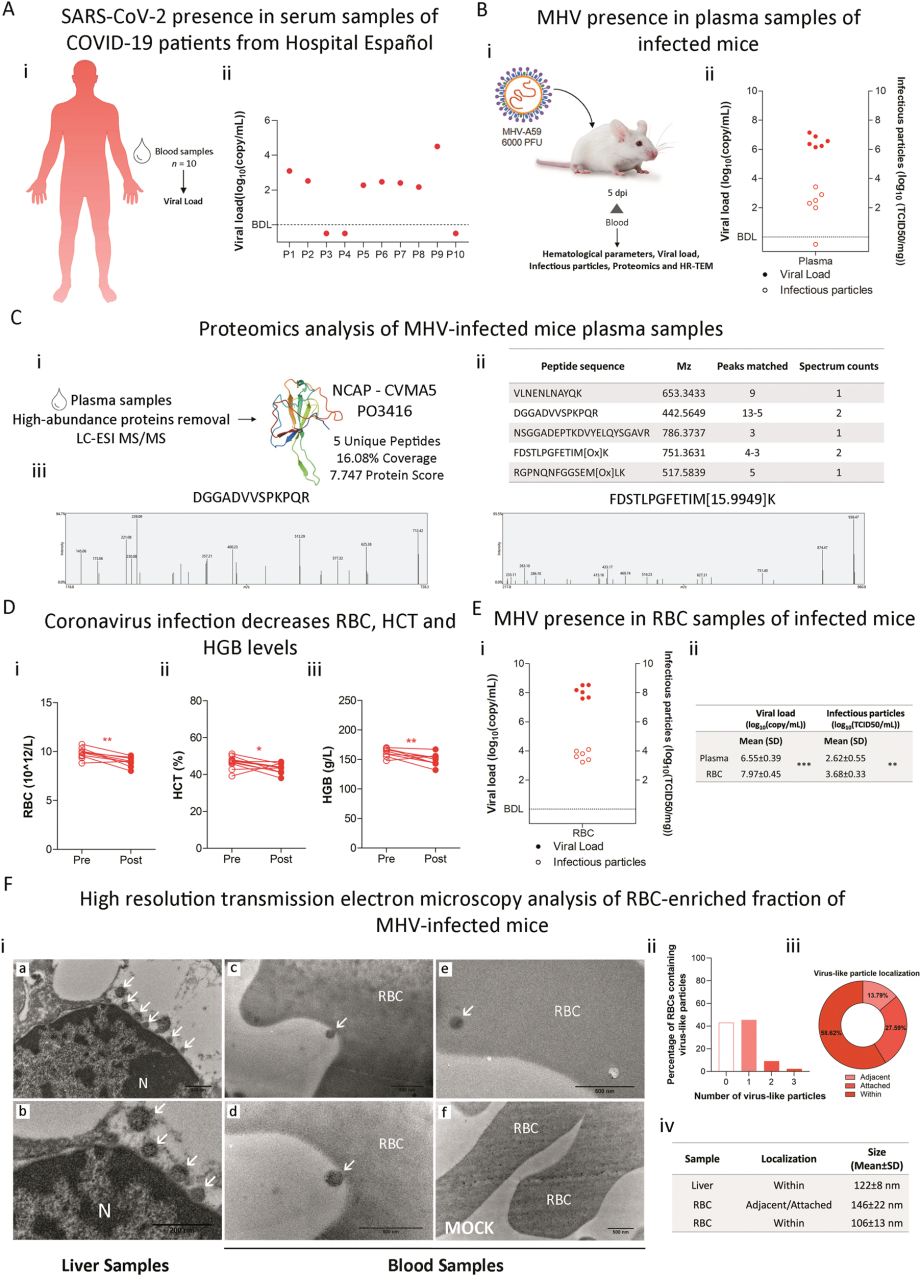
Presence of SARS-CoV-2 and MHV in blood specimens. **A** (**i**) Schematic representation of sample acquisition from COVID-19 patients from Hospital Español (Administración de los Servicios de Salud del Estado, Uruguay). Blood samples (*n* = 10) were collected for viral load by RT-qPCR analysis. (**ii**) Viral load (log_10_(copy/mL) of serum samples from COVID-19 patients (P). BDL below detection limit. **B** (**i**) Experimental design of murine Coronavirus infection. BALB/cJ mice were infected with 6000 PFU of MHV-A59 by intraperitoneal injection (MHV). Blood samples were taken before and after infection to assess hematological parameters. Blood fractionation was performed by centrifugation. Five days post-infection (dpi), hematological parameters, viral load, and infectious particles were determined in plasma and red blood cell (RBC)-enriched fraction by RT-qPCR analyzes and viral plaque assays, respectively. Additionally, proteomics and high-resolution transmission electron microscopy (HR-TEM) analyzes were performed to identify viral proteins and particles. (**ii**) Presence of viral RNA and infectious particles in plasma of MHV-infected mice (*n* = 6). Viral load (log_10_(copy/mL)) measured by RT-qPCR (filled circles) and viral titers (log_10_(TCID50/mL)) determined by plaque assays (open circles). BDL below detection limit. **C** (**i**) Detection of viral proteins by LC ESI-MS/MS. MHV-infected BALB/cJ mice were subjected to blood extraction and fractionation. Plasma fractions were depleted of the 3 most abundant proteins using the Multiple affinity removal spin cartridge mouse 3 (Agilent) and analyzed by LC ESI-MS/MS. The NCAP-CVMA5 protein (P03416) was identified. (**ii**) Table depicting the 5 unique peptides found for NCAP-CVMA5 protein. Mz, the amount of peaks matched and spectrum counts are shown. (**iii**) Identification of N protein. Spectrum from 2 unique peptides found with 2 spectrum counts (DGGADVVSPKPQR and FDSTLPGFETIM[Ox]K). **D** (**i**) Hematological parameters assessment in MHV-infected (MHV), and uninfected mice (MOCK). Red blood cell (RBC) (10^12^/L) (**i**) hematocrit (HTC) (%) (**ii**), and hemoglobin (HGB) (g/L) (**iii**) levels pre- and post-infection. Paired student’s *t*-test was performed to determine statistical differences between pre- and post-infection. **E** (**i**) Presence of viral RNA and infectious particles in RBC-enriched fraction of MHV-infected mice (*n* = 6). Viral load (log_10_(copy/mL)) measured by RT-qPCR (filled circles) and viral titers (log_10_(PFU/mL)) determined by plaque assays (open circles). BDL below detection limit. (**ii**) Table depicting the mean value for viral load (log_10_(copy/mL)) and titer (log_10_(PFU/mL)) from plasma and RBC-enriched fractions. SD: standard deviation. Unpaired student’s *t*-test was performed to determine statistical differences between plasma and RBC-enriched fractions. Statistical significance was set at *p* < 0.05. *p < 0.05, ** *p* < 0.01, ***p < 0.001. **F** (**i**) High-resolution transmission electron microscopy (HR-TEM) of liver samples (**a**–**b,** (**b**) shows a magnification of what is observed in (**a**)), RBC-enriched fraction from MHV-infected (**c**–**e,** (**d**) shows a magnification of what is observed in (**c**)), and mock (**f**) mice. White arrows indicate virus-like particles with electron-dense appearance. Control samples from non-infected mice served as a baseline. Liver samples were used as a positive control of infection. Scale bar: 500 nm. RBC red blood cell, N nucleus. (**ii**) Bar plot depicting the percentage of imaging fields containing 0, 1, 2, or 3 virus-like particles, observed in the RBC-enriched fraction of MHV-infected mice. (**iii**) Pie chart depicting the localization (adjacent, attached, or within) of virus-like particles found in the RBC-enriched fraction of MHV-infected mice. (**iv**) Table summarizing the size (mean ± SD) of virus-like particles observed within liver cells and adjacent/attached/within RBCs.

Reflecting on the widespread tissue affinity of SARS-CoV-2, we postulated that the virus might permeate various tissues through hematological routes. Viral load was assessed in serum samples from a cohort of ten COVID-19 patients (Fig. 3Ai), detecting SARS-CoV-2 RNA in 70% of these individuals (Fig. 3Aii). Pursuing a comparative analysis, we assessed the presence of MHV RNA and infectious particles in the plasma of infected mice (Fig. 3Bi, Bii). Proteomics analysis validated the presence of viral particles in this compartment, revealing five unique peptides corresponding to the MHV nucleocapsid protein (Fig. 3C).

Further, LC ESI-MS/MS was employed to compare the proteomes of pharyngeal swabs from MHV-infected and uninfected mice (Supplementary Fig. S4A), revealing a distinct proteomic profile (Supplementary Fig. S4B, S4C). Gene ontology classification of the differentially expressed proteins highlighted several categories related to erythrocytes, such as hemostasis, gas transport, and heme binding, corroborating the impact of infection on blood physiology (Supplementary Table S2–S3 and Fig. S4D). The hematological parameters of MHV-infected mice corroborated these findings, with significant decrements in RBC count (*p* < 0.01; Fig. 3Di), HCT (*p* < 0.05; Fig. 3Dii), and HGB levels (*p* < 0.01; Fig. 3Diii). Correspondingly, WBC counts declined post-infection (*p* < 0.001; Supplementary Fig. S5Ai), with a concomitant reduction in lymphocytes (*p* < 0.001; Supplementary Fig. S5Aii) and an increase in neutrophils and monocytes (*p* < 0.001 and *p* < 0.01, respectively; Supplementary Fig. S5Aiii, S5Aiv). Additionally, thrombocytopenia (PLT count) was observed (*p* < 0.001; Supplementary Fig. S5Av), with both NLR and PLR elevated post-infection (*p* < 0.01 for both cases, Supplementary Fig. S5Bi,S5Bii).

Given the alterations in hematological parameters, we posited a significant role for RBCs in the propagation of viral infection. Our subsequent investigations confirmed the presence of both viral RNA and infectious particles within the RBC-enriched fractions of MHV-infected mice (Fig. 3Ei, Eii), which were higher than those found for the plasma fractions (Fig. 3Eii), underscoring a potential mechanism for widespread viral distribution. Additionally, high-resolution transmission electron microscopy (HR-TEM) evidenced virus-like particles adsorbed onto the surface of RBCs and within RBCs (Fig. 3Fi and Supplementary Fig. S6). More than 50% of the images (56.82%) analyzed showed at least one virus-like particle (Fig. 3Fii). Regarding their localization, our analysis showed that 58.62% were found within RBCs (Fig. 3Fiii). The virus-like particles adsorbed on the surface had an average size of 146 ± 22 nm, while the particles inside the RBCs had an average size of 106 ± 13 nm (Fig. 3Fiv and Supplementary Fig. S6**)**, consistent with the average size of viral particles found in the liver (122 ± 8 nm). Collectively, these observations underscore the involvement of SARS-CoV-2 and MHV within the blood compartment, with implications for the alteration of critical blood parameters.

### Heme interaction with SARS-CoV-2 and MHV Spike proteins: structural insights and potential therapeutic implications

**Fig. 4:**
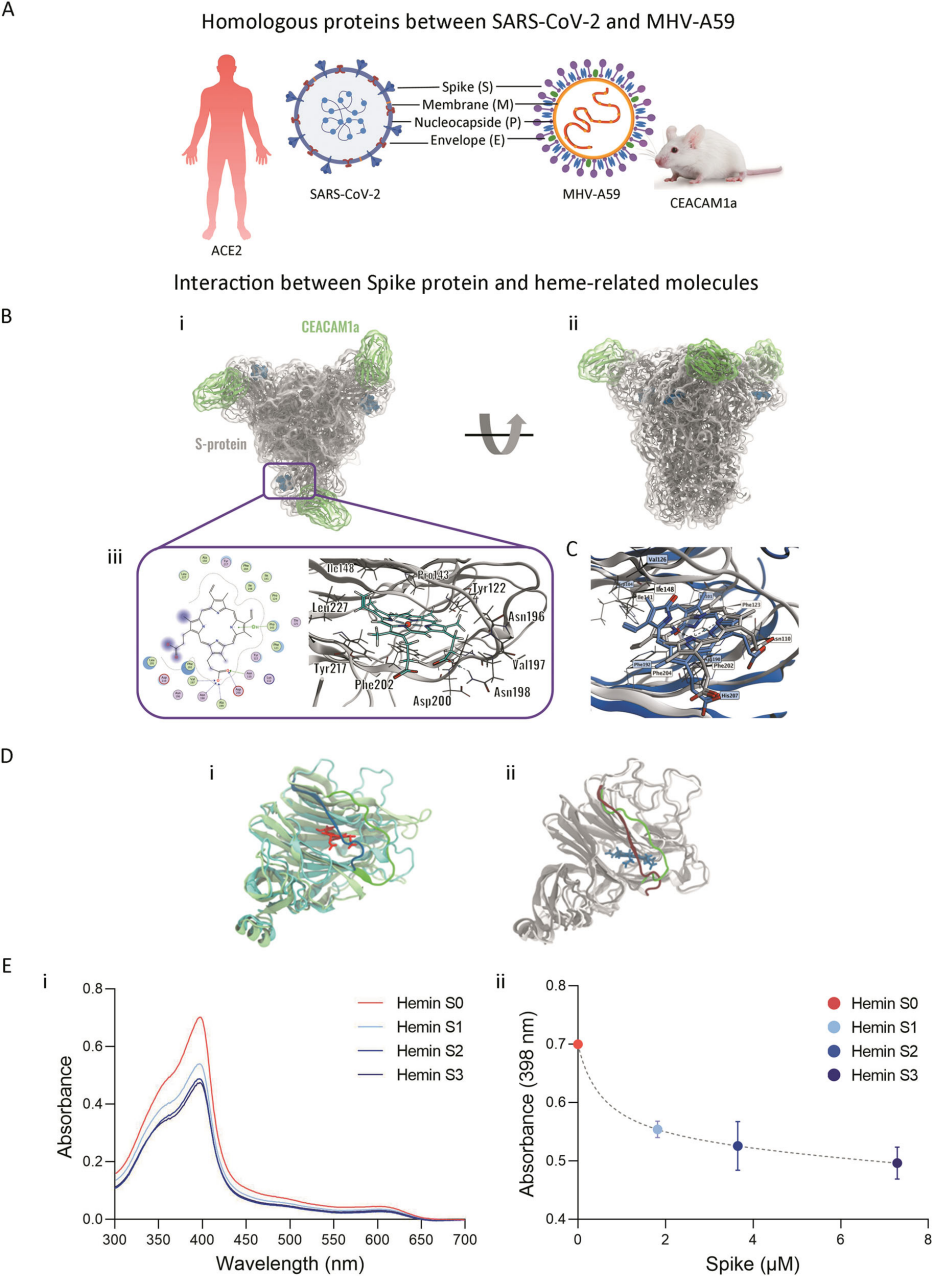
Heme can bind to the SARS-CoV-2 and MHV Spike proteins. **A** Homologous proteins and viral receptors between SARS-CoV-2 and MHV-A59. **B** (**i**) Visualization of the final MHV S protein-CEACAM1a complex from top view. (**ii**) Visualization of the final MHV S protein-CEACAM1a complex from side view. (**iii**) 2D view of the interactions between heme and the NTD binding site on the left, 3D representation of the docked heme in the beta subunit of the MHV sNTD on the right. S protein is shown in gray. CEACAM1a D1 subunit is shown in green. Heme molecules are shown in dark blue spheres. Transparent overlays represent molecular surfaces of MHV S protein and CEACAM1a. **C** Overlap of heme—MHV S protein complex (in gray, predicted by docking) and experimental biliverdin - SARS-CoV-2 S protein complex (in blue); residues involved in conserved interactions are represented as sticks and labeled. **D** (**i**) Visual comparison of the superposed SARS-CoV-2 sNTD (light green) and MHV-sNTD (cyan). (**ii**) Comparison of the Val188-Asp200 loop in the MHV-sNTD before (dark red) and after (green) manually re-sampling the loop conformation, in the presence of the biliverdin molecule (dark blue) from the SARS-CoV-2 sNTD template (PDB 7BS2). **E** (**i**) UV–visible spectra of hemin alone (10 µM, red) or in the presence of increasing amounts of purified SARS-CoV-2 Spike (S1: 1.8 µM in light blue, S2: 3.6 µM in blue, S3: 7.2 µM in dark blue). (**ii**) Determination of binding affinities (K_d_ value) between hemin (10 µM) and purified recombinant SARS-CoV-2 Spike protein (S1: 1.8 µM in light blue, S2: 3.6 µM in blue, S3: 7.2 µM in dark blue) by UV–Visible spectrophotometry. Absorbance at 398 nm, which corresponds to Soret band peak, was plotted to determine Kd using GraphPad Software. Results are shown as the mean ± SEM. Data are representative of three independent experiments.

Structural and functional homologies between MHV and SARS-CoV-2 provide profound mechanistic insights into SARS-CoV-2 pathogenesis (Fig. 4A) [19]. Specific binding affinity of heme to the N-terminal domain (NTD) of the SARS-CoV-2 Spike (S) protein has been proposed [8]; thus, we employed computational docking strategies to explore the analogous interaction between heme and the MHV S protein. We pinpointed a homologous binding site on the MHV S protein and successfully docked ferric heme into each of the three NTD subunits (Fig. 4Bi, Bii) in complex with the D1 domain of the CEACAM1a, the principal receptor for MHV entry. The binding site is lined by a series of aromatic and hydrophobic residues (Fig. 4Biii). The porphyrin ring is buried within the hydrophobic core of the pocket, forming π-stacking interactions with aromatic side chains of Tyr217 and Phe202, and enables interactions between the vinyl and methyl groups and the surrounding hydrophobic residues. Remarkably, the docking pose of heme into the putative MHV S protein site is also consistent with the experimentally solved S protein-biliverdin complex of SARS-CoV-2 (Fig. 4C,Di–ii). Key hydrophobic interactions are maintained in the MHV complex and are detailed in Supplementary Results. Additionally, molecular dynamics simulations further confirmed the conformational stability of the predicted docking pose of heme onto the binding site of S protein (Supplementary Results).

Cumulatively, our findings indicate that heme and its derivative molecules possess the capacity to bind to the MHV S protein, paralleling the binding mechanisms identified in SARS-CoV-2 [20]. To confirm the interaction between S protein and heme we performed UV-visible spectroscopy. We observed that the addition of increasing amounts of purified SARS-CoV-2 S protein showed a consistent decrease of the overall hemin Soret and Q bands (Fig. 4Ei). We estimated the dissociation constant (K_d_) for the interaction of SARS-CoV-2 S protein with hemin at 0.596 μM (Fig. 4Eii). The observed absorptivity change in the hemin spectra is consistent with a change in the hemin direct environment, probably due to binding to the S protein. The lack of change in Soret or Q band positions is consistent with hemin-S protein interaction lacking direct coordination to the heme iron, which is in agreement with our in silico-based heme binding mode (Fig. 4B, C). Additionally, similar results were obtained when we analyzed hemin-binding proteins in conditioned media from non-infected (mock) and MHV-infected L929 cells as we observed a decrease of the Soret and Q bands when proteins from MHV-infected cells were incubated with hemin (Supplementary Fig. 7A). To further validate our results on the impact of heme on virus infectivity on this Coronavirus model, we evaluated the effect of hemin on MHV infection in vitro, revealing that a higher viral titer is observed in the presence of hemin (Supplementary Fig. 7B).

Overall, our results suggest a conserved mode of interaction between S protein and heme-related molecules that could have profound implications for the pathogenesis of Coronavirus infections and their potential interventions.

### Hemin-induced augmentation of viral infection in the MHV model

**Fig. 5:**
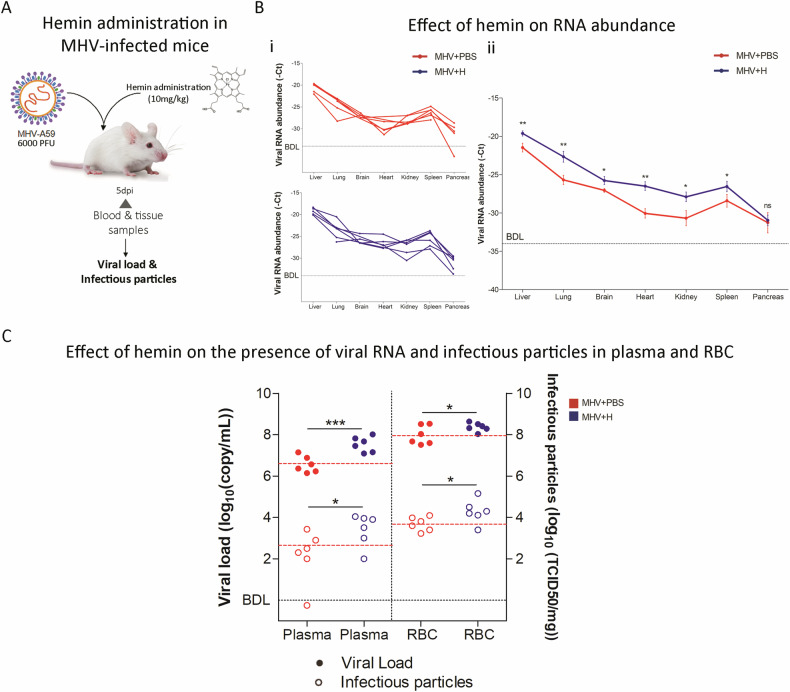
Hemin-induced augmentation of viral infection in the MHV model. **A** Experimental design of murine Coronavirus infection. BALB/cJ mice were infected with 6000 PFU of MHV-A59 by intraperitoneal injection (MHV). Mice were treated with hemin (a single dose of 10 mg/kg, *i.p*.). Five days post-infection (dpi), the liver, lung, brain, heart, kidney, spleen, and pancreas were dissected for RT-qPCR analyses. Blood samples were also taken pre- and post-infection. Blood fractionation was performed by centrifugation. Additionally, viral load and infectious particles were determined in plasma and RBC-enriched fraction by RT-qPCR analyzes and viral plaque assays, respectively. **B** Viral RNA abundance (-Ct), measured by RT-qPCR, in liver, lung, brain, heart, kidney, spleen, and pancreas samples from MHV + PBS (*n* = 6, red) and MHV + H (*n* = 6, blue) mice. Each dot represents the mean value of three technical replicates (**i**) or biological replicates (**ii**). Viral RNA abundance is shown as the mean ± SEM. **C** Viral load (log_10_(copy/mL)), measured by RT-qPCR (filled circles), and viral titers (log_10_(PFU/mL), determined by plaque assays (open circles), in the plasma and RBC-enriched fractions from MHV-infected mice. Red and blue dots represent MHV-infected mice (*n* = 6) and MHV-infected and treated with hemin mice (*n* = 6), respectively. Red dashed lines represent the mean value of control group (MHV + PBS). BDL below detection limit. Unpaired student’s *t*-test was performed to determine statistical differences between MHV + PBS and MHV + H. Statistical significance was set at *p* < 0.05. *p < 0.05, ***p < 0.001.

Given the potential of RBCs as targets for Coronavirus infection and the predictive binding of heme to viral proteins, we investigated the influence of hemin, a heme analog, on the course of MHV infection. MHV-infected mice were administered hemin (10 mg/kg, *i.p*.) to probe the exogenous heme impact (Fig. 5A). Subsequent analysis performed five dpi revealed no discernible macroscopic or histological effects of hemin compared to MHV-infected mice (Supplementary Figs. S2 and S3C). Biochemical assays also indicated no significant alterations on ALB, GLO, ALT, AST, or GLU levels between MHV-infected mice with and without hemin treatment (MHV + H vs. MHV, Supplementary Fig. S8Ai–Aiv,S8C). Kidney pathology remained unaltered by hemin (Supplementary Fig. S8Bi), although BUN levels were elevated in MHV + H vs. MHV (Supplementary Fig. S8Bii).

Notably, viral RNA abundance in the liver (*p* < 0.01), lung (*p* < 0.01), brain (*p* < 0.05), heart (*p* < 0.01), kidney (*p* < 0.05), and spleen (*p* < 0.05) of hemin-treated mice (MHV + H) was higher in comparison to their untreated counterparts (MHV), with this enhancement not extending to the pancreas (Fig. 5Bi, Bii). Furthermore, a significant increment in viral load was detected in both plasma and RBC-enriched fractions of the hemin-treated group (*p* < 0.01 and *p* < 0.05, respectively; Fig. 5C), alongside heightened viral titers within these fractions (*p* < 0.05 for both; Fig. 5C). These findings implicate heme as a facilitator of Coronavirus infection, promoting viral infectivity within host cells.

### Chloroquine mitigating effects of hemin-enhanced MHV infection

**Fig. 6:**
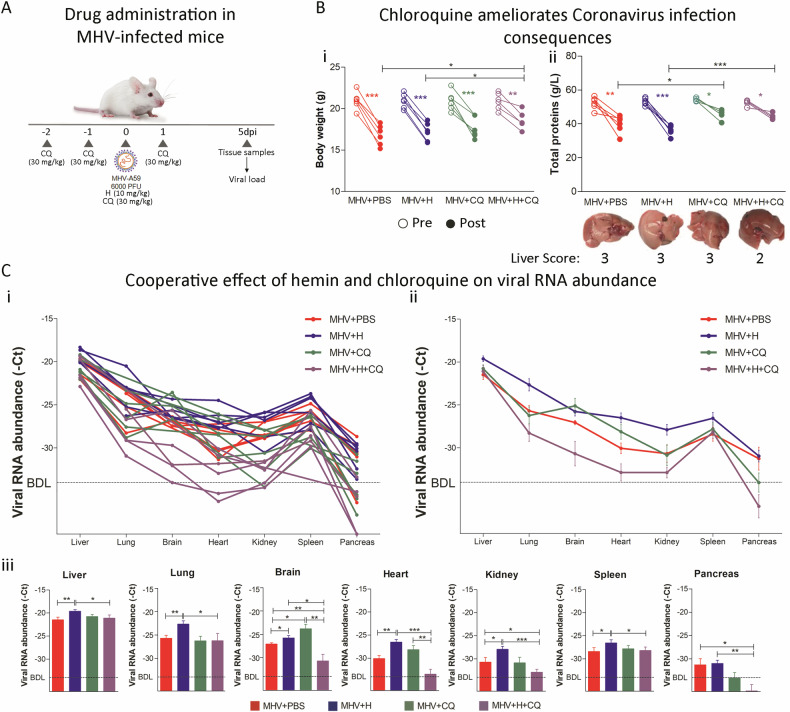
Hemin and chloroquine combined treatment reversed the enhanced infection promoted by hemin. **A** Experimental design of murine Coronavirus infection. BALB/cJ mice were infected with 6000 PFU of MHV-A59 by intraperitoneal injection. Mice were treated with chloroquine (CQ) (four doses of 30 mg/kg, *i.p*.) (MHV + CQ) and/or hemin (a single dose of 10 mg/kg, *i.p*.) (MHV + H and MHV + H + CQ). Infected untreated mice (MHV + PBS) received 100 µL of PBS by *i.p*. injection. Five days post-infection (dpi), the liver, lung, brain, heart, kidney, spleen, and pancreas were dissected for RT-qPCR analyzes and viral plaque assays. **B** (**i**) Body weight pre- (empty circles) and post- (filled circles) infection of MHV + PBS, MHV + H, MHV + CQ, and MHV + H + CQ mice. (**ii**) Total protein (g/L) levels measured in the blood of MHV + PBS, MHV + H, MHV + CQ, and MHV + H + CQ mice, pre- (empty circles) and post- (filled circles) infection (Upper panel). Liver macroscopic appearance (Lower panel). **C** Viral RNA abundance (-Ct), measured by RT-qPCR, in liver, lung, brain, heart, kidney, spleen, and pancreas samples from all of the MHV + PBS (*n* = 6), MHV + H (*n* = 6), MHV + CQ (*n* = 6) and MHV + H + CQ (*n* = 6) mice (**i**) mean ± S.E.M of each group (**ii**) and bar plot for each organ depicting statistical differences between groups (**iii**). Each dot represents the mean value of three technical replicates (**i**) or biological replicates (**ii**). Viral RNA abundance is shown as the mean ± SEM. BDL below detection limit. Statistical significance was set at *p* < 0.05. *p < 0.05, **p < 0.01, ***p < 0.001.

CQ known to interact and induce structural modifications in heme group [21], was evaluated for its capacity to counteract the hemin-mediated increase in Coronavirus infectivity. MHV-infected mice were treated with either hemin (10 mg/kg, *i.p.)*, CQ (30 mg/kg, *i.p*.), or their combination (Fig. 6A). Subsequent assessments five dpi showed that while CQ alone did not affect the body weight (Fig. 6Bi), its combination with hemin partially mitigated the weight loss associated with MHV infection (*p* < 0.05; Fig. 6Bi) and ameliorated liver pathology and function (*p* < 0.001; Fig. 6Bii). Further, histological analysis showcased that livers from MHV-infected mice treated with H + CQ present less cytoplasmic vacuolation than MHV + H group (Supplementary Fig. S2). CQ alone did not improve the macroscopic or histological appearance of the liver or kidneys compared to the MHV group (Fig. 6Bii, Supplementary Figs. S2 and S7Bi) and had no impact on other organs (Supplementary Fig. S3C line 4). Biochemical parameters were unaltered by CQ compared to the control group (Supplementary Fig. S8Ai–iv, S8Bii), whereas GLU levels were elevated in groups treated with CQ, both alone and in combination with hemin (*p* < 0.05; Supplementary Fig. S8C). Interestingly, the combination of hemin and CQ partially reverted the pathological changes associated to infection in lung and spleen. Specifically, lungs from MHV + H + CQ mice are well preserved and present mild inflammatory reaction while MHV + H group showed edema and interstitial pneumonia (Supplementary Fig. S2). Regarding spleen, histological analysis showed that MHV + H mice are characterized by congestion and the presence of abundant macrophages and multinucleated inflammatory cells while MHV + H + CQ mice only present lymphoid hyperplasia (Supplementary Fig. S2).

Concerning the presence of viral genome, MHV + CQ mice showed no significant differences in the observed organs compared with untreated MHV-infected mice, except for the brain (*p* < 0.05; Fig. 6C). However, a lower viral RNA abundance was observed in the brain (*p* < 0.01), kidney (*p* < 0.05), and pancreas (*p* < 0.05) of mice with the combined treatment compared with MHV-infected mice (Fig. 6Ciii). MHV + H + CQ mice also showed a decrease in the viral RNA abundance of liver (*p* < 0.05), lung (*p* < 0.05), brain (*p* < 0.05), heart (*p* < 0.001), kidney (*p* < 0.001), spleen (*p* < 0.05) and pancreas (*p* < 0.01) compared to MHV + H mice (Fig. 6Ciii). MHV + H + CQ mice showed lower viral RNA abundance in the brain (*p* < 0.01) and heart (*p* < 0.01) compared to MHV + CQ mice (Fig. 6Ciii). These findings collectively indicate that CQ can attenuate the pro-infective effects of hemin on Coronavirus infection.

### Hemin and chloroquine treatment attenuate viral load and normalize hematological disruptions in MHV infection

**Fig. 7:**
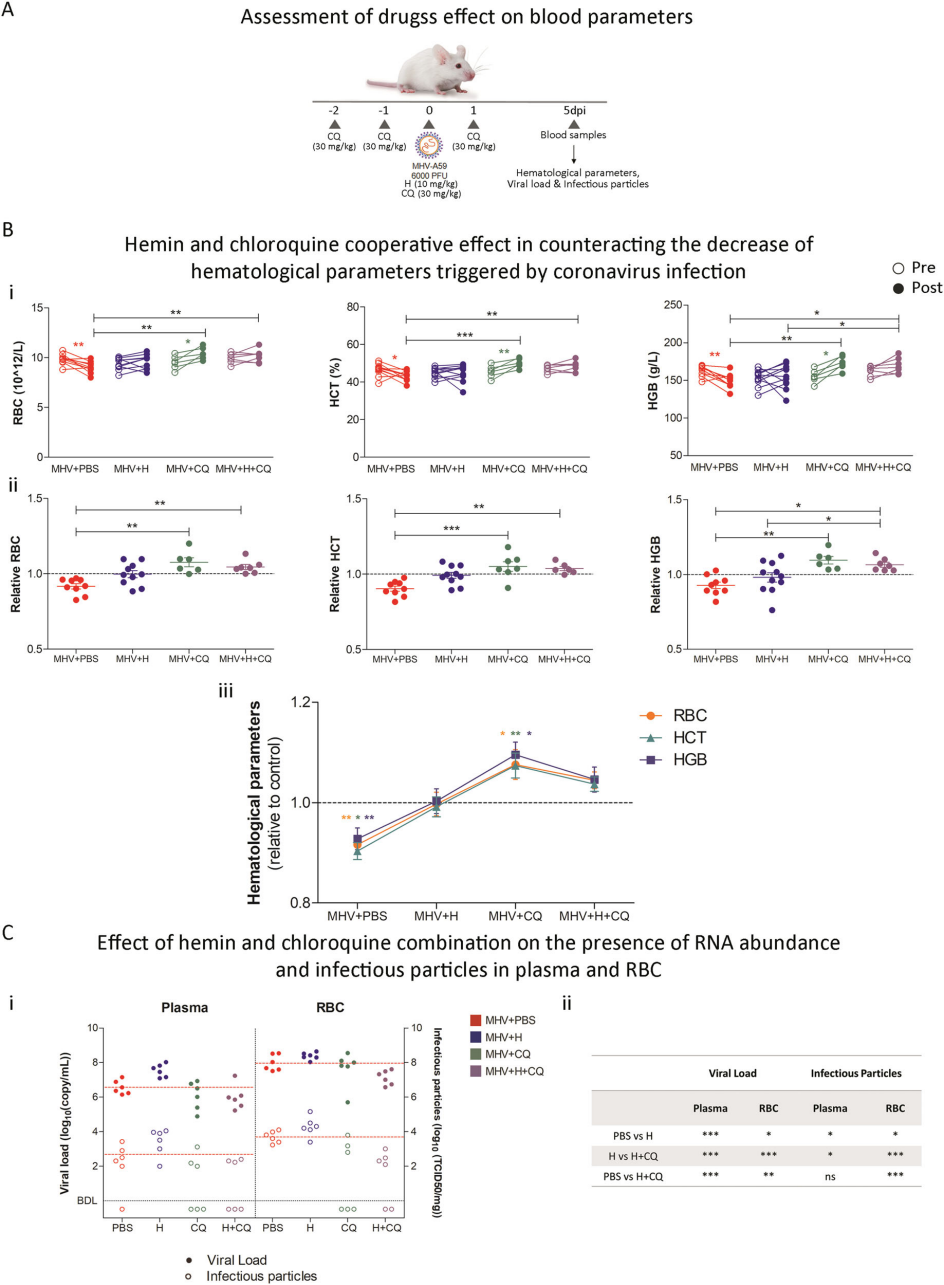
Blood biochemical profiles were improved by hemin and chloroquine combined treatment. **A** Experimental design of murine Coronavirus infection. BALB/cJ mice were infected with 6000 PFU of MHV-A59 by intraperitoneal injection. Mice were treated with chloroquine (CQ) (four doses of 30 mg/kg, *i.p*.) (MHV + CQ) and/or hemin (a single dose of 10 mg/kg, *i.p*.) (MHV + H and MHV + H + CQ). Infected untreated mice (MHV + PBS) received 100 µL of PBS by *i.p*. injection. Blood samples were also taken pre- and post-infection for hematological parameters determination. Additionally, five days post-infection (dpi), blood fractionation was performed by centrifugation, and viral load and infectious particles were determined in plasma and red blood cell (RBC)-enriched fraction by RT-qPCR analyzes and viral plaque assays, respectively. **B** Hematological parameters assessment in MHV + PBS, MHV + H, MHV + CQ, and MHV + H + CQ mice. Absolut (**i**) and relative (to the MOCK group, dashed line) (**ii**–**iii**) red blood cell (RBC) (1012/L), hematocrit (HTC) (%), and hemoglobin (g/L) levels pre- and post-infection. **C** (**i**) Viral load (log_10_(copy/mL)), measured by RT-qPCR, and viral titers (log_10_(PFU/mL), determined by plaque assays, in the plasma and RBC-enriched fractions from MHV + PBS, MHV + H, MHV + CQ, and MHV + H + CQ mice. Red, blue, green, and purple dots represent MHV + PBS (*n* = 6), MHV + H (*n* = 6), MHV + CQ (*n* = 6) and MHV + H (*n* = 6), respectively. Red dashed lines represent the mean value of control group (MHV + PBS). (**ii**) Table depicting the statistical significance between experimental groups. Unpaired student’s *t*-test was performed to determine statistical differences between conditions. Statistical significance was set at *p* < 0.05. * *p* < 0.05, **p < 0.01, ***p < 0.001.

Blood biochemical profiles (Fig. 7A) of both MHV + CQ and MHV + H + CQ groups were significantly restored compared to MHV infection alone (*p* < 0.01 for RBC, *p* < 0.001, and *p* < 0.01 for HCT, *p* < 0.01, and *p* < 0.05 for HGB; Fig. 7B). Hemin by itself seems to improve hematological parameters, however these changes were not significant compared to the MHV + PBS group (Fig. 7B). Furthermore, WBC counts were not impacted by either treatment (Supplementary Fig. S5Ai). However, MHV + H + CQ partially impaired the reduction in lymphocyte and the increase in neutrophil percentages when compared to MHV mice (*p* < 0.01 for both parameters; Supplementary Fig. S5Aii, S5Aiii), without affecting monocytes or PLT (Supplementary Fig. S5Aiv,S5Av). Interestingly, MHV + CQ mice showed an elevated PLR (*p* < 0.01; Supplementary Fig. S5Bii), while MHV + H + CQ treatment was associated with a decreased NLR compared to MHV-only mice (*p* < 0.01; Supplementary Fig. S5Bi), and a higher PLR compared to the MHV + H group (*p* < 0.05; Supplementary Fig. S5Bii).

Finally, the combined treatment resulted in significantly lower viral load in the plasma fraction compared to MHV-infected mice (*p* < 0.001 for both cases; Fig. 7C) and in a lower viral load and titer compared to MHV + H mice (*p* < 0.001 and *p* < 0.05; Fig. 7C). Moreover, the combined treatment also resulted in a significant decrease in viral load and titer in the RBC-enriched fraction compared to MHV-infected mice (*p* < 0.01 and *p* < 0.001, respectively; Fig. 7C) and to MHV + H mice (*p* < 0.001 for both cases; Fig. 7C). These comprehensive findings underscore the efficacy of CQ in mitigating the pro-viral effects of hemin treatment and curbing the spread of viral particles within the bloodstream.

## Discussion

In this study, we present novel findings on the presence of Coronavirus genetic material and its infectious capacity within the blood compartment. We provide evidence that Coronaviruses may exploit heme from lysed RBCs, shedding light on its potential role in systemic viral dissemination. Our in silico analyses have demonstrated the capability of MHV S protein to bind heme, mirroring interactions observed with SARS-CoV-2 [8]. This binding may contribute to the observed increase in viral infectivity when heme is abundant, as evidenced in our animal model. Significantly, our data indicate that CQ can mitigate the pro-infective influence of hemin, reversing hematological disturbances and supporting the hypothesis that heme interactions play a role in Coronavirus pathogenicity.

The perception of COVID-19 has evolved from a purely respiratory illness to one with recognized multisystemic effects. Hematological abnormalities, including leukopenia, thrombocytopenia, and coagulopathies, are frequently observed in severe cases of COVID-19 [22]. Notably, functional RBC anomalies have been implicated in the etiology of persistent post-acute sequelae of SARS-CoV-2 infection [23].

Our findings showcase the virus’s multiple organotropism, corroborating observations in human cases [24]. While early in the SARS-CoV-2 outbreak comprehensive multi-organ autopsies were scarce, limiting multi-organ viral detection, subsequent analyses and emerging literature affirm viral presence in the heart and kidneys across patients [25]. The lower viral detection in patient sera, in contrast to the pronounced viral presence in plasma and RBCs in our model, may be the reliance of viral particles on clotting factors, absent in serum but present in plasma, which includes PLTs known to express various viral receptors [26, 27].

A growing body of evidence links abnormal RBC dynamics to COVID-19, with several studies suggesting that the virus may induce hemolysis [5, 28–30]. There is increasing interest in therapeutic agents that target hemolysis as a protective strategy against severe manifestations of the disease. Our results align with findings that demonstrate heme binding to viral S protein and protein 7a, highlighting the potential role of labile heme in exacerbating hemolytic conditions in COVID-19 patients[6–8].

Reports indicating elevated levels of free heme in patients with severe COVID-19 compared to those with milder disease support the theory that the virus may exploit RBC lysis [31]. Our experimental introduction of hemin, a heme analog, into MHV-infected mice supports this hypothesis, with a notable increase in viral RNA across multiple organs. Furthermore, we observed augmented viral load and particles in both plasma and RBCs following hemin administration. Intriguingly, like SARS-CoV-2, MHV-A59 lacks hemagglutinin-esterase [32, 33], suggesting that hemin associates with alternative viral proteins. Our findings thus reinforce the concept that heme availability may confer a survival advantage to the virus, facilitating its replication and propagation.

In the wake of our findings, we postulate that the affinity of hemin/heme for viral proteins confers a distinct advantage to the virus in translocating across the host system. To mitigate this process, we turned to CQ, a molecule with a well-known affinity for heme [21], hypothesizing that it could neutralize the facilitative effect of hemin on viral spread. CQ documented antiviral properties against diverse viruses [34, 35] positioned it as a potential repurposed drug during the early stages of the COVID-19 pandemic [36, 37], though clinical trials yielded mixed results [38]. This multifaceted drug, however, may not have been fully evaluated within the context of its interaction with the blood compartment. Our study confirmed that the H + CQ regimen led to a marked reduction in viral RNA abundance across most organs. This was accompanied by a notable decrease in both viral load and infectivity in plasma and RBCs when compared with MHV infection alone or MHV + H. Additionally, we observed improvements in blood parameters following H + CQ treatment, suggesting that the presence of heme—whether supplemented artificially or derived from hemolysis—and its predicted binding to the viral S protein may confer an evolutionary advantage to the virus. Altogether, our data suggests that CQ can indeed disrupt the viral advantage provided by heme, pointing to a need for a re-evaluation of its therapeutic potential, particularly considering its effect on blood-based viral dissemination.

Our research has elucidated that the multi-organ virulence of Coronavirus infection is paralleled by the presence of viral particles within the blood compartment, underlining the potential role of RBCs in viral infectivity. This finding bears significant implications for blood-related abnormalities observed in COVID-19 patients. Thus, blood parameters could play a pivotal role in the development of targeted treatments for individuals with severe COVID-19 manifestations.

### Limitations of the study

Our study unveils a novel pathway of SARS-CoV-2 dissemination via hemoproteins and introduces the potential of CQ as a modulating agent, a novel insight that could pivot the direction of COVID-19 therapeutic strategies. However, limitations to this study include the small number of patients and the lack of clinical data. Additionally, we also consider that the use of hamsters or a mouse-adapted SARS-CoV-2 strain would be more appropriate, however our institutions do not have BSL3 animal facility.

## Supplementary information

Supplementary Information

## Data Availability

The datasets generated and/or analyzed during the current study are available from the corresponding author on reasonable request. Proteomics data is already available at the ProteomeXchange Consortium via the PRIDE partner repository with the dataset identifier PXD054355.

## References

[1] Raveendran AV, Jayadevan R, Sashidharan S. Long COVID: an overview. Diabetes Metab Syndr. 2021;15:869–75. 33892403 10.1016/j.dsx.2021.04.007 PMC8056514

[2] Davis HE, McCorkell L, Vogel JM, Topol EJ. Long COVID: major findings, mechanisms and recommendations. Nat Rev Microbiol. 2023;21:133–46. 36639608 10.1038/s41579-022-00846-2 PMC9839201

[3] Lopez-Leon S, Wegman-Ostrosky T, Ayuzo del Valle NC, Perelman C, Sepulveda R, Rebolledo PA. et al. Long-COVID in children and adolescents: a systematic review and meta-analyses. Sci Rep. 2022;12:9950. 35739136 10.1038/s41598-022-13495-5 PMC9226045

[4] Korompoki E, Gavriatopoulou M, Fotiou D, Ntanasis-Stathopoulos I, Dimopoulos MA, Terpos E. Late-onset hematological complications post COVID-19: an emerging medical problem for the hematologist. Am J Hematol. 2022;97:119–28. 34687462 10.1002/ajh.26384 PMC8646944

[5] Sahu KK, Borogovac A, Cerny J. COVID-19 related immune hemolysis and thrombocytopenia. J Med Virol. 2021;93:1164–70. 32776538 10.1002/jmv.26402 PMC7436763

[6] Hopp M-T, Rathod DC, Imhof D. Host and viral proteins involved in SARS-CoV-2 infection differentially bind heme. Protein Sci. 2022;31:e4451. 36161737 10.1002/pro.4451 PMC9538320

[7] Lechuga GC, Souza-Silva F, Sacramento CQ, Trugilho MRO, Valente RH, Napoleão-Pêgo P. et al. SARS-CoV-2 proteins bind to hemoglobin and its metabolites. Int J Mol Sci. 2021;22:9035. 34445741 10.3390/ijms22169035 PMC8396565

[8] Rosa A, Pye VE, Graham C, Muir L, Seow J, Ng KW, et al. SARS-CoV-2 can recruit a heme metabolite to evade antibody immunity. Sci Adv. 2021;7:eabg7607. 10.1126/sciadv.abg7607 PMC8163077 33888467

[9] Freeman SL, Oliveira ASF, Gallio AE, Rosa A, Simitakou MK, Arthur CJ, et al. Heme binding to the SARS-CoV-2 spike glycoprotein. J Biol Chem. 2023;299:105014. 37414149 10.1016/j.jbc.2023.105014 PMC10416065

[10] Körner RW, Majjouti M, Alejandre Alcazar MA, Mahabir E. Of mice and men: the Coronavirus MHV and mouse models as a translational approach to understand SARS-CoV-2. Viruses. 2020;12:880. 32806708 10.3390/v12080880 PMC7471983

[11] Pereira-Gómez M, Fajardo Á, Echeverría N, López-Tort F, Perbolianachis P, Costábile A, et al. Evaluation of SYBR Green real time PCR for detecting SARS-CoV-2 from clinical samples. J Virol Methods. 2021;289:114035. 33285190 10.1016/j.jviromet.2020.114035 PMC7831559

[12] Leonardi DB, Anselmino N, Brandani JN, Jaworski FM, Páez AV, Mazaira G, et al. Heme oxygenase 1 impairs glucocorticoid receptor activity in prostate cancer. Int J Mol Sci. 2019;20:1006. 30813528 10.3390/ijms20051006 PMC6429053

[13] Ramos-Avila A, Ventura-Gallegos JL, Zentella-Dehesa A, Machuca-Rodríguez C, Moreno-Altamirano MM, Narváez V, et al. Immunomodulatory role of chloroquine and pyrimethamine in Plasmodium yoelii 17XL infected mice. Scand J Immunol. 2007;65:54–62. 17212767 10.1111/j.1365-3083.2006.01869.x

[14] Yang M, Cao L, Xie M, Yu Y, Kang R, Yang L, et al. Chloroquine inhibits HMGB1 inflammatory signaling and protects mice from lethal sepsis. Biochem Pharmacol. 2013;86:410–8. 23707973 10.1016/j.bcp.2013.05.013 PMC3713089

[15] Vigerust DJ, McCullers JA. Chloroquine is effective against influenza A virus in vitro but not in vivo. Influenza Other Respi Viruses. 2007;1:189–92. 10.1111/j.1750-2659.2007.00027.x PMC4941887 19453426

[16] Arévalo AP, Pagotto R, Pórfido JL, Daghero H, Segovia M, Yamasaki K, et al. Ivermectin reduces in vivo coronavirus infection in a mouse experimental model. Sci Rep. 2021;11:7132. 33785846 10.1038/s41598-021-86679-0 PMC8010049

[17] Arévalo AP, Pagotto R, Pórfido JL, Daghero H, Segovia M, Yamasaki K, et al. Ivermectin reduces in vivo coronavirus infection in a mouse experimental model. Sci Rep. 2021;11:7132. 33785846 10.1038/s41598-021-86679-0 PMC8010049

[18] Gupta A, Madhavan MV, Sehgal K, Nair N, Mahajan S, Sehrawat TS, et al. Extrapulmonary manifestations of COVID-19. Nat Med. 2020;26:1017–32. 32651579 10.1038/s41591-020-0968-3 PMC11972613

[19] Körner RW, Majjouti M, Alcazar MAA, Mahabir E. Of mice and men: the Coronavirus MHV and mouse models as a translational approach to understand SARS-CoV-2. Viruses. 2020;12:880. 32806708 10.3390/v12080880 PMC7471983

[20] Rosa A, Pye VE, Graham C, Muir L, Seow J, Ng KW, et al. SARS-CoV-2 can recruit a heme metabolite to evade antibody immunity. Science Advances. 2021;7:17. 10.1126/sciadv.abg7607 PMC8163077 33888467

[21] Bachhawat K, Thomas CJ, Surolia N, Surolia A. Interaction of chloroquine and its analogues with heme: an isothermal titration calorimetric study. Biochem Biophys Res Commun. 2000;276:1075–9. 11027592 10.1006/bbrc.2000.3592

[22] Mina A, van Besien K, Platanias LC. Hematological manifestations of COVID-19. Leuk Lymphoma. 2020;61:2790–8. 32643489 10.1080/10428194.2020.1788017

[23] Brundyn J-L, Gillan J, Singh I. Hematologic abnormalities associated with post-acute COVID-19 sequelae or “long-COVID”-a systematic review. Int J Biomed Lab Sci. 2022;11:23–42.

[24] Bian X-W. Autopsy of COVID-19 patients in China. Natl Sci Rev. 2020;7:1414–8. 34192086 10.1093/nsr/nwaa123 PMC7313767

[25] Stein SR, Ramelli SC, Grazioli A, Chung J-Y, Singh M, Yinda CK, et al. SARS-CoV-2 infection and persistence in the human body and brain at autopsy. Nature. 2022;612:758–63. 36517603 10.1038/s41586-022-05542-y PMC9749650

[26] Antoniak S, Mackman N. Platelets and viruses. Platelets. 2021;32:325–30. 33615982 10.1080/09537104.2021.1887842 PMC7987802

[27] Panteleev MA, Sveshnikova AN, Shakhidzhanov SS, Zamaraev AV, Ataullakhanov FI, Rumyantsev AG. The ways of the virus: interactions of platelets and red blood cells with SARS-CoV-2, and their potential pathophysiological significance in COVID-19. Int J Mol Sci. 2023;24:17291. 38139118 10.3390/ijms242417291 PMC10743882

[28] Marshall M. The lasting misery of coronavirus long-haulers. Nature. 2020;585:339–41. 32929257 10.1038/d41586-020-02598-6

[29] Al-Kuraishy HM, Al-Gareeb AI, Kaushik A, Kujawska M, Batiha GE-S. Hemolytic anemia in COVID-19. Ann Hematol. 2022;101:1887–95. 35802164 10.1007/s00277-022-04907-7 PMC9263052

[30] Al-Aly Z, Xie Y, Bowe B. High-dimensional characterization of post-acute sequelae of COVID-19. Nature. 2021;594:259–64. 33887749 10.1038/s41586-021-03553-9

[31] Huang C, Wang Y, Li X, Ren L, Zhao J, Hu Y, et al. Clinical features of patients infected with 2019 novel coronavirus in Wuhan, China. Lancet. 2020;395:497–506. 31986264 10.1016/S0140-6736(20)30183-5 PMC7159299

[32] Kazi L, Lissenberg A, Watson R, de Groot RJ, Weiss SR. Expression of hemagglutinin esterase protein from recombinant mouse hepatitis virus enhances neurovirulence. J Virol. 2005;79:15064–73. 16306577 10.1128/JVI.79.24.15064-15073.2005 PMC1316009

[33] Zandi M, Behboudi E, Soltani S. Role of glycoprotein hemagglutinin-esterase in COVID-19 pathophysiology? Stem Cell Rev Rep. 2021;17:2359–60. 10.1007/s12015-021-10210-1 PMC8237253 34181186

[34] Devaux CA, Rolain J-M, Colson P, Raoult D. New insights on the antiviral effects of chloroquine against coronavirus: what to expect for COVID-19? Int J Antimicrob Agents. 2020;55:105938. 32171740 10.1016/j.ijantimicag.2020.105938 PMC7118659

[35] Oscanoa TJ, Romero-Ortuno R, Carvajal A, Savarino A. A pharmacological perspective of chloroquine in SARS-CoV-2 infection: an old drug for the fight against a new coronavirus? Int J Antimicrob Agents. 2020;56:106078. 32629115 10.1016/j.ijantimicag.2020.106078 PMC7334645

[36] Cortegiani A, Ingoglia G, Ippolito M, Giarratano A, Einav S. A systematic review on the efficacy and safety of chloroquine for the treatment of COVID-19. J Crit Care. 2020;57:279–83. 32173110 10.1016/j.jcrc.2020.03.005 PMC7270792

[37] Gies V, Bekaddour N, Dieudonn‚ Y, Guffroy A, Frenger Q, Gros F, et al. Beyond anti-viral effects of chloroquine/hydroxychloroquine. Front Immunol. 2020;11:1409. 32714335 10.3389/fimmu.2020.01409 PMC7343769

[38] Huang M, Li M, Xiao F, Pang P, Liang J, Tang T, et al. Preliminary evidence from a multicenter prospective observational study of the safety and efficacy of chloroquine for the treatment of COVID-19. Natl Sci Rev. 2020;7:1428–36. 34676087 10.1093/nsr/nwaa113 PMC7313782

