# Supplementary material for: Blood matters: the hematological signatures of Coronavirus infection
Source: Cell Death Dis. 2024 Nov 28;15(11):863. doi: 10.1038/s41419-024-07247-8 (PMC11605097; doi:10.1038/s41419-024-07247-8)
Supplement: Supplementary file 1 — Supplementary Information [file 41419_2024_7247_MOESM1_ESM.docx]

SUPPORTING INFORMATION TEXT

1. SUPPLEMENTARY METHODS

**SUPPLEMENTARY VIRAL LOAD ASSESSMENT METHODS**

For the quantification standard, a qPCR product of 161 bp was cloned into pCR™2.1-TOPO® using the TOPO® TA Cloning® Kit (Invitrogen) following manufacturer’s instructions and transformed in NEB® 5-alpha Competent E. coli (High Efficiency) by the heat shock method (42°C, 30 s). Plasmids were isolated using PureLink Quick Plasmid Miniprep Kit (Invitrogen) and spectrophotometrically quantified (Bio-photometer, Eppendorf). Next, 1 μg of plasmid DNA was linearized with SpeI and in vitro transcribed with T7 RNA Polymerase (Thermo Fisher Scientific) following manufacturer’s instructions. In vitro transcribed RNA was treated with Dnase, purified with TURBO DNA-free™ Kit (Thermo Fisher Scientific) and fluorometrically quantified (Qubit 2.0, Thermo Fisher Scientific). The number of copies/μL was calculated as: $\frac{N_{A}\times C}{MW}$, where N_A_ is the Avogadro constant expressed in mol–1, C is the concentration expressed in g/μL, and MW is the molecular weight expressed in g/mol. A stock containing 9.5 × 1010 copies/μL of the RNA in vitro transcribed was used for standard curve by 10-fold serial dilutions in triplicates. Standard curve was represented as Ct vs log copy number/reaction. The linear regression curve (y) was determined as y = -3.7x + 46.1 and the coefficient of determination (R2) was equal to 0.9917.

**HISTOLOGICAL METHODS**

Histological analysis was performed as described before[1]. Briefly, immediately after necropsy, the organs were fixed in 10% neutral buffered formalin (pH 7.4) for further processing. For evaluation, they were embedded in paraffin, sectioned in 4 µm sections and stained with hematoxylin–eosin (H&E) according to Kyuwa et al*.*[2]. Whole specimens were examined under a light microscope (BX41, Olympus, Tokyo, Japan) at 10 × in three randomly selected areas or in the highest incidence areas of each specimen by a pathologist to perform a histopathological analysis in each case.

**SUPPLEMENTARY HIGH RESOLUTION TRANSMISSION ELECTRON MICROSCOPY METHODS**

Blood from mock and MHV-infected mice (500 µl) was collected in an Eppendorf tube containing ethylenediaminetetraacetic acid (EDTA 1:10) and centrifuged at 3000 rpm for 15–20 min. After discarding the plasma and buffy coat layers, erythrocyte fraction was mixed for fixation with a solution of paraformaldehyde 2% vol/vol and glutaraldehyde 1% vol/vol in phosphate buffer 0.1M (1:1). Sample preparation continued with Karnovsky's fixative used in phosphate buffer 0.1 M pH 7.2-7.4, ON at 4°C; it was post-fixed in osmium tetroxide 1% vol/vol and routine protocol was followed. Centrifugations were performed at 3000 rpm for 5 min, and it was included in Aralidite. Ultrathin sections (70 nm) were raised on Copper grids with formvar film. The images were taken by a high-resolution transmission electron microscope (HR-TEM JEOL JEM 2100, 200Kv) at the High-Resolution Laboratory of CURE Technological Development Department, Rocha, University of the Republic (Uruguay).

**SUPPLEMENTARY PROTEOMIC METHODS**

*Sample collection and protein extraction from pharyngeal swabs*

For proteomics analysis, 12 BALB/cJ female mice (8–10 weeks old) were distributed into two groups according to the experiment: a) control mock infected (MOCK, n = 6) intranasal administered dilute in 10 µL of vehicle (PBS) and b) MHV-infected (MHV, n = 6) intranasal administered 15,000 PFU diluted in 10 µL of vehicle (PBS). Five days after the infection, pharyngeal swabs were collected using a sterile cotton swab (Puritane) and stored in a falcon tube at room temperature. Protein extraction from swabs containing a 60% acetonitrile (ACN), 0.1% trifluoracetic acid (TFA) solution was performed by 1 h of vigorous agitation.

*Sample collection and protein extraction from plasma fractions*

For proteomics analysis, 10 BALB/cJ female mice (8–10 weeks old) were distributed into two groups according to the experiment: a) control mock infected (MOCK, n = 5) intraperitoneally administered with vehicle (PBS) and b) MHV-infected (MHV, n = 5) intraperitoneally administered 6,000 PFU diluted in vehicle (PBS). Five days after the infection, blood samples were collected and blood fractionation was performed as mentioned in the main methods section. Plasma fractions were depleted from albumin, transferrin and G immunoglobins using Multiple Affinity Removal Spin Cartridge Mouse-3 (Agilent Technologies). 30 ug of protein were purified by SDS-Page.

*LC-MS/MS and data analysis (Pharyngeal swabs)*

Samples were dried and resuspended in 6M urea, 50 mM ammonium bicarbonate (AMBIC), pH 8.0. Disulfide bonds reduction and Cys alkylation were performed with 10 mM dithiothreitol (DTT) (1 h, RT) and 55mM iodoacetamide (IAA) (45 min, RT, in the dark), respectively. Urea was diluted to 1M previous to protein digestion with 0.5 ug of sequence grade trypsin (Promega) overnight at RT. Tryptic peptides were desalted using C18 Ziptips (Millipore) and analyzed on a nano-HPLC UltiMate 3000 (Thermo Sc.) coupled to a Q-Orbitrap mass spectrometer Q Exactive Plus (Thermo Sc.) through an Easy-Spray source (Thermo Sc.). Samples were loaded into an Acclaim PepMap^TM^ 100 C18 trap column (75 µm x 2 cm, 3 µm particle size, Thermo Sc.) and separated in a PepMap^TM^ RSLC C18 analytical column (75 µm x 50 cm, 2 µm particle size, 100 Å, Thermo Sc.) at 40 °C using a two-solvent system: (A) 0.1% formic acid (FA) in water, (B) 0.1% FA in ACN. Separation was achieved through an elution gradient as follows: 1% to 35% B over 90 min and 35% to 99% B over 20 min, at 200 nL/min. The mass spectrometer was set in positive mode with a top-12 data-dependent acquisition method. Ion spray voltage and capillary temperature were set at 2.0 kV and 250 °C, respectively. The survey scans were acquired between 200 and 2000 m/z with a resolution of 70000 at 200 m/z, an AGC target value of 1E6 and a maximum ion injection time of 100 ms. Precursor fragmentation occurred in an HCD cell with a resolution of 17500 at 200 m/z, an AGC target value of 1E5 and a maximum ion injection time of 50 ms. Precursor ions with unassigned, single, five and higher charge states were excluded for fragmentation. Normalized collision energy was used in steps of NCE 25, 30 and 35. Dynamic exclusion time was set to 5 s.

PatternLab for Proteomics software (<http://www.patternlabforproteomics.org>) [3,4] was used for peptide identification and label-free quantitative analysis. MS raw files were searched against a target-reverse database containing sequences from *Mus musculus* and Murine Hepatitis Virus strain A59 (MHV-A59) downloaded from UniProt (http://www.uniprot.org, 11/12/2020) and the most common contaminants in proteomics experiments. For data search, m/z precursor tolerance was set at 40 ppm. Methionine oxidation and cysteine carbamidomethylation were defined as variable and fixed modifications respectively. A maximum of 2 missed cleavages and 2 variable modifications per peptide were allowed. Search results were filtered by the Search Engine Processor (SEPro) algorithm from PatternLab with a maximum FDR value ≤ 1% at protein level and 10 ppm tolerance for precursor ions. Proteomic data from both groups were normalized by adding the total number of identified spectra in each sample. PatternLab’s Venn diagram statistical module was used to determine proteins exclusively detected in each biological condition with a p-value < 0.05. PatternLab’s TFold module was used to relatively quantify proteins present in both biological conditions using a label-free quantification method based in spectral counting. Proteins present in at least 3 biological replicates from the total of 6 per condition were considered for TFold analysis. This module uses the Benjamini-Hochberg’s theoretical estimator to deal with multiple T-tests, identifications satisfying a fold change cutoff that varies with the p-values (BH q<0.05) are considered significant. The resulting proteins obtained from Venn diagram and TFold Patternlab’s modules, were subjected to gene ontology (GO) analysis using the *clusterProfiler* package [5].

*LC-MS/MS and data analysis (Plasma)*

Protein samples were separated up to 1 cm into an SDS-PAGE resolving gel, fixed and stained with Coomassie blue R-250. In-gel protein digestion and peptide extraction were performed as previously described [6] with minimal variations. Briefly, the gel bands containing the total proteins were excised, and the cysteine residues were sequentially reduced with 10 mM dithiothreitol (DTT) and alkylated with 55 mM iodoacetamide (IAA). Tryptic digestion was performed in-gel by overnight incubation at 37°C with sequencing grade trypsin (Promega) in a protease:protein ratio of 1:50 (w/w). Tryptic peptides were extracted from the gel by adding 60% ACN / 0.1% trifluoroacetic acid (TFA) in two steps of 1h incubation at 30°C. Samples were dried under vacuum and peptides were desalted using in-house made StageTips packed with Empore^TM^ Octadecyl C18 SPE membrane (Supelco, 66883-U). Eluted peptides were vacuum dried and dissolved with 0.1% formic acid (FA).

Tryptic peptides were analyzed on a nano-HPLC UltiMate 3000 (Thermo Sc.) coupled to an Orbitrap Exploris 240 mass spectrometer (Thermo Sc.) through an Easy-Spray source (Thermo Sc.). Samples were loaded into an Acclaim PepMap^TM^ 100 C18 trap column (75 µm x 2 cm, 3 µm particle size, Thermo Sc.) and separated in a PepMap^TM^ RSLC C18 analytical column (75 µm x 50 cm, 2 µm particle size, 100 Å, Thermo Sc.) at 40 °C using a two-solvent system: (A) 0.1% formic acid (FA) in water, (B) 0.1% FA in ACN. Separation was achieved through an elution gradient as follows: 1% to 35% B over 150 min and 35% to 99% B over 15 min, at 200 nL/min. The mass spectrometer was set in positive mode with a top-20 data-dependent acquisition method. Ion spray voltage and capillary temperature were set at 2.0 kV and 250 °C, respectively. The survey scans were acquired between 200 and 2000 m/z with a resolution of 90000 at 200 m/z. Precursor fragmentation occurred in an HCD cell with a resolution of 22500 at 200 m/z. The AGC target value and the maximum ion injection time were set to Standard and Auto, respectively, for full and fragmentation scans. Precursor ions with unassigned, single and higher than five charge states were excluded for fragmentation. Normalized collision energy was used in steps of NCE 25, 30 and 35. Dynamic exclusion time was set to 10 s.

PatternLab for Proteomics software (<http://www.patternlabforproteomics.org>) [3,4] was used for peptide identification and label-free quantitative analysis. MS raw files were searched against a target-reverse database containing sequences from *Mus musculus* and Murine Hepatitis Virus strain A59 (MHV-A59) downloaded from UniProt (http://www.uniprot.org, 17/10/2023) and the most common contaminants in proteomics experiments. For data search, m/z precursor tolerance was set at 35 ppm. Methionine oxidation and cysteine carbamidomethylation were defined as variable and fixed modifications respectively. A maximum of 2 missed cleavages and 2 variable modifications per peptide were allowed. Search results were filtered by the Search Engine Processor (SEPro) algorithm from PatternLab with a maximum FDR value ≤ 1% at protein level and 10 ppm tolerance for precursor ions.

*Proteomic data availability*

The mass spectrometry proteomics data have been deposited to the ProteomeXchange Consortium via the PRIDE partner repository [7] with the dataset identifier PXD054355.

**SUPPLEMENTARY COMPUTATIONAL METHODS**

*System Preparation*

The MHV Spike (S) protein is a large glycosylated homotrimer composed of three identical subunits, and its structure in complex with the CEACAM1a receptor has recently been solved with a resolution of 3.94 Å and released with PDB ID 6VSJ [8]. To obtain additional insights into the binding between heme and the MHV S protein, we prepared a computational model of the latter starting from the aforementioned 6VSJ structure. As a first step, the missing residues in the structure (residues 483-493 and 832-853) were modelled as unstructured loops using the loop modeler tool of MOE 2022 [9]. Residues 1170 to 1227 in the C-terminal domain, which are also missing in the solved structure, were excluded, given that the focus of the present investigation is on the N-terminal domain (NTD), to reduce the computational cost. The complete model was subsequently protonated at physiological pH (7.4) and salt concentration (0.15M) and subjected to a first round of energy-minimization in MOE using the Amber10:EHT force field, with the reaction field scheme to account for solvation and with non-bonded cutoffs of 0.8 and 1 nm, until the RMS gradient fell below 0.1 kcal/mol/A [9]. The three CEACAM1a subunits included in the reference structure (residues Glu35 to His142) were also retained and included in this procedure.

*Determination and refinement of the heme binding site*

No experimentally solved crystal structure is available to this date of heme in complex with the MHV S protein. However, recent studies have shown that biliverdin, a product of heme catabolism with high similarity to the heme molecule, binds to each of the three NTDs of the SARS-CoV-2 S glycoprotein, and the complex between the two has been experimentally solved with 1.82Å resolution (PDB ID 7B62) [10], highlighting a specific binding site located between two sets of beta-sheets (residues Phe186-Leu212 and Ile101-Cys131, respectively) on each NTD. This information was used to guide the placement of three heme molecules on the MHV S protein NTDs (MHV-sNTD) according to the following procedure: after preparing the MHV S protein structure and fixing all missing residues and gross atomic clashes as previously described, we aligned the MHV-sNTD with the NTD domain of the experimental SARS-Cov-2 S protein-biliverdin complex (PDB 7B62). Alignment was performed in MOE using the structure-assisted alignment, which relies on the Mean Square Distance deviation of matching protein atoms as described in Shapiro et al. [11] to aid the sequence alignment. This methodology optimizes the superposition of matching secondary structures even in the case of poorer sequence identity, as is the case for the S proteins of different coronaviruses (the two NTDs in this study share a sequence similarity of 16.5%).

**Figure 4D** reports the detail of the superposition between the MHV-sNTD (in cyan) and the NTD of SARS-CoV-2 with its complexed biliverdin molecule (in light green and red, respectively). Notably, there is a good structural superposition between the MHV-sNTD and the SARS-CoV-2 NTD beta sheets forming the biliverdin binding site, despite the comparably low sequence identity. However, in the case of MHV-sNTD, the loop lining the outside portion of the binding site (residues Val188 to Asp200, shown in dark blue in **Supplementary Figure S8A**) is much shorter than the corresponding loop in the SARS-CoV-2 S protein (shown in bright green), resulting in a tighter binding site opening, as also highlighted by the clash between the superposed biliverdin molecule and the MHV-sNTD loop. Thus, while the overall secondary structure of the binding site region seems to be conserved, the significant difference in steric hindrance at the binding site entry between the two NTDs in their experimentally solved conformation. Indeed, the original work by Rosa et al. [10] discusses the mobility of this loop, which leads to a gate-like mechanism locking the biliverdin molecules into place after binding. Thus, after aligning the two NTDs and the biliverdin molecule as discussed, we manually refined the Val188-Asp200 loop of each MHV-sNTD subunit as follows: new conformations of the loop, in the presence of the biliverdin molecule, were obtained using the loop modeler utility in MOE, using an RMSD rejection cutoff of 1 Å, a loop limit of 50 and an energy window of 50 kcal/mol, in the AMBER10:EHT [12] force field. Among the top-scoring loops, we retained the conformation that was most similar to the corresponding loop in the SARS-CoV-2 template, i.e. the one allowing for proper ligand accommodation without clashes.

The result of this procedure is shown for one MHV-sNTD in **Supplementary Figure S9**. The re-sampled conformation is more similar to the corresponding loop of the SARS-CoV-2 sNTD template and prevents atomic clashes with the reference binding pose of biliverdin as obtained experimentally.

After completing the loop remodeling procedure for all three subunits, the final MHV S-biliverdin complex was visually inspected and the sidechain of residue Ile148 of subunit B was repacked due to a clash with one of the biliverdin rings. The complex was then subjected to a further round of energy minimization, with the same parameters as described above, to relax any remaining clashes or unfavourable sidechain positions. The final position of the biliverdin molecules in the MHV-sNTD clefts was finally used as a reference to perform docking of the heme ligand to the target MHV-sNTD.

*Docking of heme onto the MHV S protein*

The atomic model of heme was obtained as a 3D-SDF file from PubChem (CID 26945). The ligand was prepared by first assigning the correct protonation state (in absence of the Fe^3+^ ion) using the *Protonate3D* tool and subsequently energy-minimizing the correctly protonated ligand in MOE. The ligand was finally docked to each of the three MHV-sNTD by using the position of the aligned biliverdin molecule as a reference, after loop remodelling as described above. Docking was performed in MOE according to the following protocol: for each of the three clefts, 100 initial poses were generated using the triangle matcher algorithm and London dG (REF) scoring function. The poses were subsequently refined with the receptor kept rigid and re-scored using the GBVI/WSA dG scoring function. The top 10 poses were retained, and among the best scoring conformations, we retained the pose for which the position of the porphyrin rings featured the best overlap with the original biliverdin molecule, and for which (a) the relative orientation of the methyl and vinyl groups in the pocket was conserved with respect to the original biliverdin and (b) both propionic acid groups faced the outside of the binding cleft, as in the case of biliverdin. Given that the difference in predicted affinity among the top 10 poses was below the thermal noise level of 0.6 kcal/mol, this selection did not imply significant energy penalties.

*Glycosylation of the MHV S-protein*

The S protein of coronaviruses is a glycoprotein featuring a variable number of glycosylation sequins of type NXS or NXT (with X being any residue except P), depending on the specific family and variant. Many studies have analyzed the glycosylation pattern of the S protein in great detail (*51*–*54*), especially in the context of the recent research effort against SARS-CoV-2, and the importance of the glycan shield for other coronaviruses such as SARS-CoV-2 has been confirmed by previous experimental evidence and by highly detailed computational studies [17]. Thus, we decided to explicitly glycosylate the MHV S-protein model to account for their role in ligand binding, conformational variability, and immune evasion. Since to the best of our knowledge the exact glycan composition of MHV S is yet to be experimentally determined, we fully N-glycosylated our MHV S-protein model using the following criteria:

1. Glycosylation sites that were present in the original experimental structure 6VSJ were fully N-glycosylated with oligo-mannose Man_[5-9]_GlcNAc_2_ type glycans, chosen with random length, which have been shown to be the dominant type of glycans on coronaviruses [18,19];
2. Glycosylation sites that are conserved between the SARS-CoV-2 S-protein and MHV S-protein were N-glycosylated using the dominant glycans reported in Watanabe et al. [13] for the SARS-CoV-2 virus.
3. All remaining NXS and NXT motifs in the MHV S-protein sequence were fully N-glycosylated using Man_[5-9]_GlcNAc_2_ type glycans with random length, provided they were accessible on the protein surface and not buried.

Glycans were added using the *Glycan Reader & Modeler* tool [20] included in CHARMM-GUI suite [21]. The final model features a total of 45 N-glycosylation sites (15 on each of the three subunits). Asparagine sidechains were manually checked and adjusted whenever necessary to enable the fitting of the glycans. The final glycan composition is reported in **Supplementary Table 4**.

## Molecular Dynamics simulations

All-atom molecular dynamics simulations of the assembled systems with and without bound heme were carried out in GROMACS 2022.3 [22] on the Béluga cluster maintained by Calcul Québec and the Digital Research Alliance of Canada. The simulations were performed following the suggested CHARMM-GUI protocol [20] in a dodecahedric box with periodic boundary conditions: after an initial 5000 steps of energy minimization using the steepest descent algorithm, systems were equilibrated in the NVT ensemble for 250 ps with a time step of 1 fs, using the Nose-Hoover [23] thermostat with a reference temperature of 303.15K. We subsequently equilibrated the systems in the NPT ensemble for 500 ps (with a time step of 1 fs) using the Nosé-Hoover thermostat and the isotropic Berendsen [24] barostat with a reference pressure of 1 bar and a time constant of 5.0 ps. Finally, production MD simulation were performed in the NPT ensemble for 1 microsecond each, with a time step of 2 fs, relying on the velocity rescale thermostat [25] and the Parrinello-Rahman barostat [26], with reference values of 303.15 K and 1 bar, respectively. Electrostatic interactions were described using the PME algorithm [27], and short-range interactions were cut off at 0.9 nm. Van-der-Waals interactions were also cutoff at 0.9 nm without any additional modifiers. The lengths of the bonds involving hydrogen were constrained using the LINCS algorithm [28].

## MD data analysis

Simulations were analyzed using the integrated GROMACS tools (for RMSD, RMSF and SASA calculations) as well as custom python scripts using MDAnalysis [29]. To calculate the amount of SASA of the S-protein ectodomain shielded by glycans, we subtracted the SASA of the S protein in the absence of the glycan shield from the same SASA calculated with the bound glycans, similarly to what is described in [17].

Data were sampled every 100 ps unless otherwise specified, and the first 150 ns of both simulations (*apo* and *holo*) were regarded as additional structural equilibration and discarded from the analyses. Visualizations of the molecular systems were created using the VMD software [30].

**METHODS TO ANALYSE HEMIN-BINDING PROTEINS**

*SARS-CoV-2 Spike protein production*

S2 stable cell line expressing EctoS-ST was obtained as described before [31] by co-transfection with the respective expression vector and pCoPuro selection plasmid using Effectene reagent (QIAGEN). Transfected cells were selected at 28°C in Insect Xpress medium (LONZA) added with 6 μg/mL puromycin. Stable S2 cell line was grown in glass flasks at 28°C with 110 rpm agitation in standard orbital shaker and induced at 5 × 10^6^ cells/mL with 5 μM CdCl_2_. After 4 days of induction, cells were harvested by centrifugation at 150g for 5 min, and both proteins were purified from the culture supernatant by AC. For this, the supernatant was centrifuged at 6.000g, filtered through 0.22 μm and loaded in a 5 mL Strep-Tactin®XT 4Flow® columns (IBA), following the manufacturer's recommendations.

Strep-tag was removed by incubating overnight with Enterokinase-His (Genscript) (20 U mg^−1^), followed by overnight dialysis in 0.1 M Tris-Cl pH 8.0, 0.15 M NaCl, incubation with Ni-Sepharose® (Cytiva) for 2 h and finally subjected to a second AC step in 0.1 M Tris-Cl pH 8.0, 0.15 M NaCl, 1 mM EDTA (storage buffer).

Finally, recombinant protein was subjected to SEC in storage buffer, using Superdex 75 and 200 columns (Cytiva) for RBD-ST/RBD^clv^ and shACE2-ST/shACE2^clv^, respectively.

The SARS-CoV-2 Spike protein sequence was the following:

RSCVNLTTRTQLPPAYTNSFTRGVYYPDKVFRSSVLHSTQDLFLPFFSNVTWFHAIHVSGTNGTKRFDNPVLPFNDGVYFASTEKSNIIRGWIFGTTLDSKTQSLLIVNNATNVVIKVCEFQFCNDPFLGVYYHKNNKSWMESEFRVYSSANNCTFEYVSQPFLMDLEGKQGNFKNLREFVFKNIDGYFKIYSKHTPINLVRDLPQGFSALEPLVDLPIGINITRFQTLLALHRSYLTPGDSSSGWTAGAAAYYVGYLQPRTFLLKYNENGTITDAVDCALDPLSETKCTLKSFTVEKGIYQTSNFRVQPTESIVRFPNITNLCPFGEVFNATRFASVYAWNRKRISNCVADYSVLYNSASFSTFKCYGVSPTKLNDLCFTNVYADSFVIRGDEVRQIAPGQTGKIADYNYKLPDDFTGCVIAWNSNNLDSKVGGNYNYLYRLFRKSNLKPFERDISTEIYQAGSTPCNGVEGFNCYFPLQSYGFQPTNGVGYQPYRVVVLSFELLHAPATVCGPKKSTNLVKNKCVNFNFNGLTGTGVLTESNKKFLPFQQFGRDIADTTDAVRDPQTLEILDITPCSFGGVSVITPGTNTSNQVAVLYQDVNCTEVPVAIHADQLTPTWRVYSTGSNVFQTRAGCLIGAEHVNNSYECDIPIGAGICASYQTQTNSPNNARSVASQSIIAYTMSLGAENSVAYSNNSIAIPTNFTISVTTEILPVSMTKTSVDCTMYICGDSTECSNLLLQYGSFCTQLNRALTGIAVEQDKNTQEVFAQVKQIYKTPPIKDFGGFNFSQILPDPSKPSKRSFIEDLLFNKVTLADAGFIKQYGDCLGDIAARDLICAQKFNGLTVLPPLLTDEMIAQYTSALLAGTITSGWTFGAGAALQIPFAMQMAYRFNGIGVTQNVLYENQKLIANQFNSAIGKIQDSLSSTASALGKLQDVVNQNAQALNTLVKQLSSNFGAISSVLNDILSRLDPPEAEVQIDRLITGRLQSLQTYVTQQLIRAAEIRASANLAATKMSECVLGQSKRVDFCGKGYHLMSFPQSAPHGVVFLHVTYVPAQEKNFTTAPAICHDGKAHFPREGVFVSNGTHWFVTQRNFYEPQIITTDNTFVSGNCDVVIGIVNNTVYDPLQPELDSFKEELDKYFKNHTSPDVDLGDISGINASVVNIQKEIDRLNEVAKNLNESLIDLQELGKYEQYIKWPGPFEDDDDKAGWSHPQFEKGGGSGGGSGGGSWSHPQFEK

Number of amino acids: 1240

Molecular weight: 137155.67

Theoretical pI: 5.92

Extinction coefficients:

Extinction coefficients are in units of M -1 cm -1 , at 280 nm measured in water.

Ext. coefficient 145355

Abs 0.1% (=1 g/l) 1.060, assuming all pairs of Cys residues form cystines.

Ext. coefficient 143480

Abs 0.1% (=1 g/l) 1.046, assuming all Cys residues are reduced.

*Protein purification and quantification from supernatants.*

1 mL of supernatant was concentrated from each infected and uninfected monolayer of L929 cells using the Vivaspin® 20, 100 kDa MWCO Polyethersulfone (Millipore, Sigma) at 600g for 10 min to obtain a volume of 60-100 µL. Then a volume of 2X lysis buffer (200mM Tris-HCl pH 8.0, 300mM NaCl, 20% Glycerol, 1.2% Triton X-100) was added to each sample and incubated on ice for 20 min. Finally, each lysate was centrifuged at 12500 g for 20 min at 4ºC and the supernatants were collected. Then the supernatant samples were diluted (1/4-1/6) in water and mixed with 150 uL of Pierce™ 660nm Protein Assay Reagent (Thermo Scientific™) following the manufacturer’s instructions in a multi-well plate. In parallel, the standard BSA curve of 2 mg/mL – 0.05 mg/mL was performed. The absorption spectrum at 660 nm was obtained with the Varioskan™ LUX multimode microplate reader (Thermo Scientific™) with 1 min of agitation before the measurement.

*UV-visible spectra*

Hemin (550 μM in NaOH 0.5 N followed by dilution to 10 μM in DMSO 40%) was added to a quartz cuvette containing 5, 10 or 20 μg of SARS-CoV-2 recombinant Spike protein or 5 μg of protein extract obtained from MHV infected or non-infected L929 cells. The spectrophotometric analysis was carried out at 300–700 nm using a Cary 60 UV-Vis Spectrophotometer (Agilent Technologies).

1. **SUPPLEMENTARY RESULTS**

**SUPPLEMENTARY RESULTS RELATED TO MOLECULAR DYNAMICS**

*Extended results of comparison between MHV and SARS-CoV-2 docking with heme*

Key hydrophobic interactions between the ligand and Ile101, Val126 and Phe192 in the SARS-CoV-2 system are maintained in the MHV complex predicted by docking, with Phe123, Ile148 and Phe204, respectively (**Figure 4C**). Additional hydrophobic interactions between the ligand and Trp104 and His207 observed in the SARS-CoV-2 complex are replaced by analogous interactions with Ile141 and Phe202 in the MHV complex. Finally, a hydrogen bond between one of the propionic acid groups and Asn110 is predicted by docking in the MHV complex, similar to the one observed between biliverdin and Arg190 residue of SARS-CoV-2 S protein. Importantly, a similar pattern of interactions between heme and SARS-CoV-2 S protein, as predicted by molecular modeling, was suggested in a recent study [32].

*Glycan shield*

Molecular dynamics (MD) simulations of the glycosylated S-protein trimer in complex with the CEACAM1a D1 domains, in the presence and in the absence of bound heme, were run. The system is visualized in **Supplementary Figure S8**. Our results were consistent for all MHV S protein subunits, as indicated by the similar docking scores obtained (-9 to -11 kcal/mol), with only minor differences caused by slight fluctuations in sidechain positioning after the initial energy minimization of the system. In addition to docking, we assembled a fully glycosylated model of the MHV S-protein and performed molecular dynamics simulations of the glycosylated S-protein trimer in complex with the CEACAM1a D1 domains, in the presence and in the absence of bound heme (**Supplementary Figure S8**). Structural stability and convergence of the simulation was assessed through RMSD analysis of the S protein. After an initial 150 ns of structural equilibration, the S trimer reached structural stability both in the control simulation and in the simulation with bound heme (**Supplementary Figure S8C**). In addition, we assessed the stability of the bound heme by calculating its RMSD throughout the simulation. As highlighted in **Supplementary Figure S8E**, the ligands remained stable in their binding site after the initial equilibration phase, with no unbinding or major conformational rearrangement observed throughout the 1000ns of sampling, except for the ligand bound to subunit B, which showed a minor rearrangement within the cleft after about 600 ns.

The use of molecular dynamics simulations of a glycosylated MHV S-protein model allowed us to observe the role of the glycans bound to the S surface. More in detail, the high mobility of the 45 total glycans (15 on each S-protein subunit) masks part of the solvent-exposed protein surface, as highlighted in **Supplementary** **Figure S8D**. This process has been suggested to be involved in immune evasion in other coronaviruses [S2–4], and might additionally play a role in the stability of the heme molecule bound in the sNTD. Indeed, glycans at sites Asn192 are located at the entrance of the heme binding site, and their mobility effectively alters the solvent-accessible portion of the site and influences the movement of the Lys190-Asn198 loop, which influences the conformational freedom of the bound ligand. Overall, the high flexibility of the N-linked glycans is responsible for a shielding effect on the S-protein surface, which reduces the solvent-accessible surface area (SASA) of the S-protein by up to 12.96 ± 0.86 %, with little differences both in total SASA and in shielded SASA between the *apo* and *holo* simulations.

1. SUPPLEMENTARY FIGURES


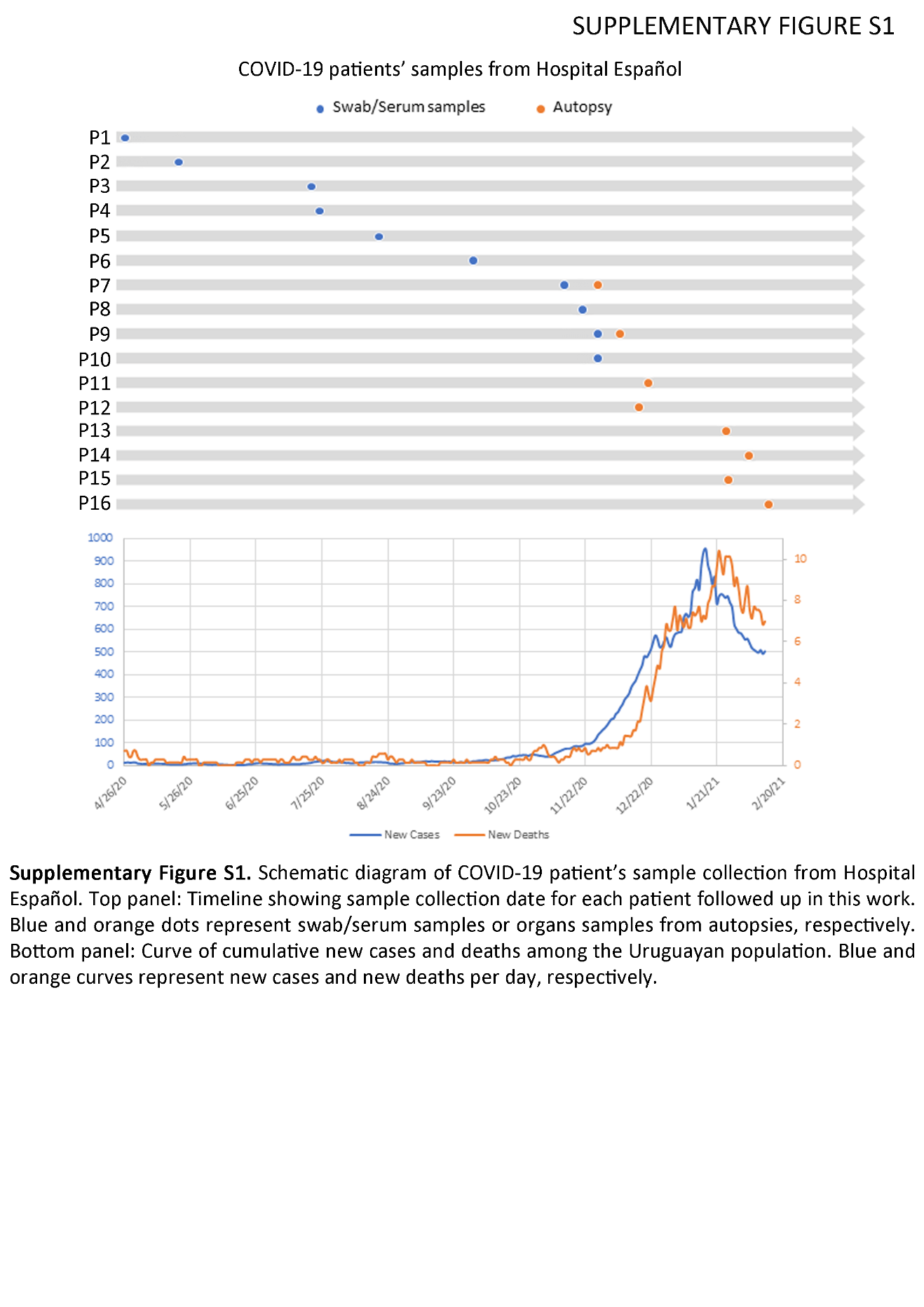


Supplementary Figure S1. Schematic diagram of COVID-19 patients’ sample collection from Hospital Español. Top panel: Timeline showing sample collection date for each patient followed up in this work. Blue and orange dots represent swab/serum samples or tissue samples from autopsies, respectively. Bottom panel: Curve of cumulative new cases and deaths among the Uruguayan population. Blue and orange curves represent new cases and new deaths per day, respectively.

**
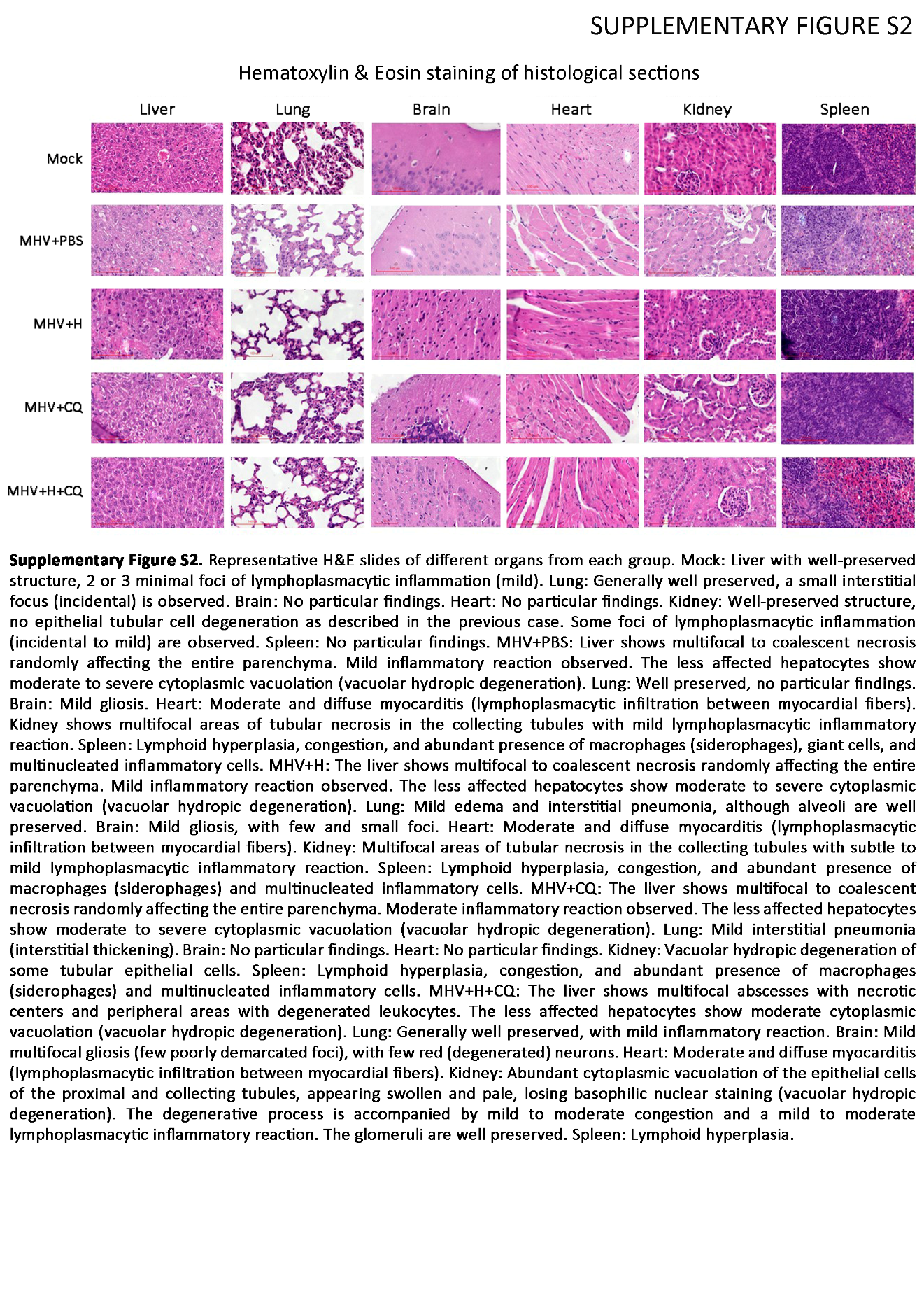
**

**Supplementary Figure S2.** Representative Hematoxylin & Eosine slides of liver, lung, brain, heart, kidney and spleen from Mock, MHV+PBS, MHV+H, MHV+CQ, and MHV+H+CQ mice. Scale bar: 100 µm.


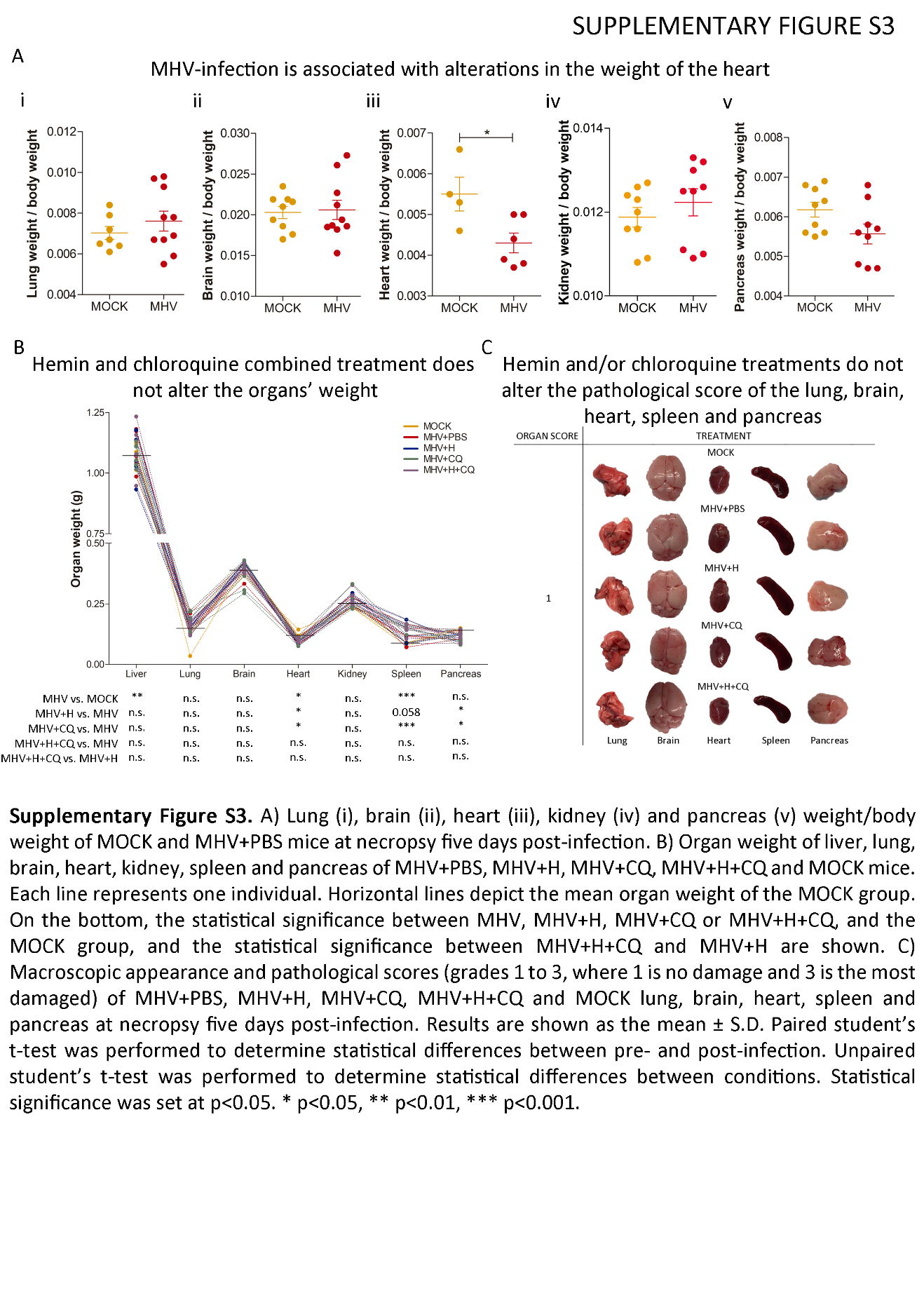


Supplementary Figure S3. A) Lung (i), brain (ii), heart (iii), kidney (iv) and pancreas (v) weight/body weight of MOCK and MHV+PBS mice at necropsy five days post-infection. B) Organ weight of liver, lung, brain, heart, kidney, spleen and pancreas of MHV+PBS, MHV+H, MHV+CQ, MHV+H+CQ and MOCK mice. Each line represents one individual. Horizontal lines depeict the mean organ weight of the MOCK group. On the bottom, the statistical significance between MHV, MHV+H, MHV+CQ or MHV+H+CQ and the MOCK group, and the statistical significance between MHV+H+CQ and MHV+H are shown. C) Macroscopic appearance and pathological scores (grades 1 to 3, where 1 is no damage and 3 is the most damaged) of MHV+PBS, MHV+H, MHV+CQ, MHV+H+CQ and MOCK lung, brain, heart, spleen and pancreas at necropsy five days post infection. Results are shown as the mean±S.D. Paired student’s t-test was performed to determine statistical differences between conditions. Statistical significance was set at p<0.05. *p<0.05, **p<0.01, ***p<0.001.


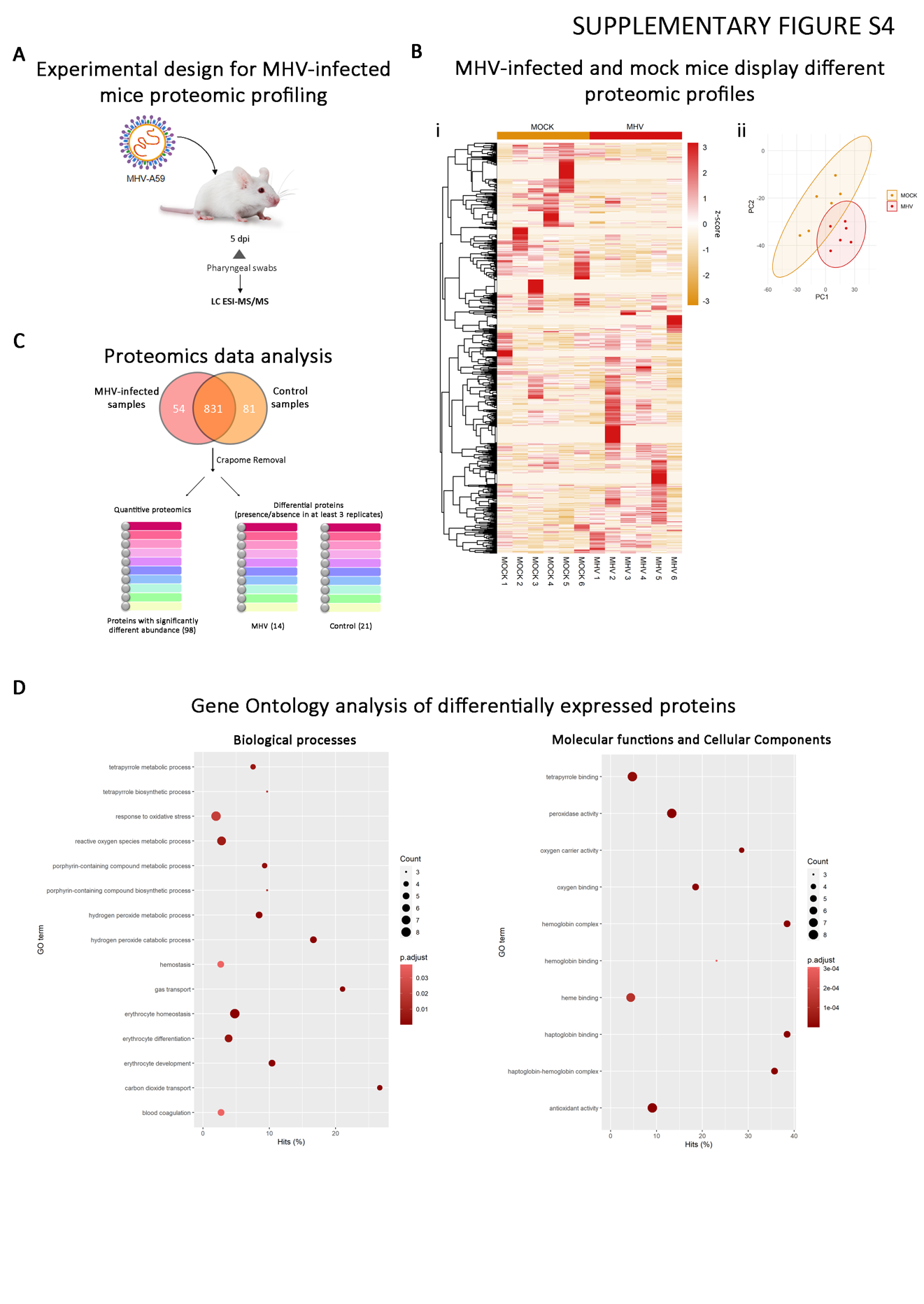
Supplementary Figure S4. A) Schematic representation of experimental design. B) i) Heatmap depicting an unsupervised clustering analysis of the proteomic profiles of pharyngeal swabs from MHV-infected (n=6, 4000 PFU) and mock (n=6) mice, considering the expression of 831 proteins. Normalized z-scores of protein abundance are shown. ii) Multidimensional Scaling (MDS) analysis of the proteomics data obtained from pharyngeal swabs showing the segregation of MHV-infected (red) and mock samples (orange). C) Data analysis was based on label-free spectral counting, obtaining an average of 885 and 912 proteins in MHV-infected and mock proteomes, respectively. 54 proteins were only found in MHV samples, while 81 were differentially detected in mock samples and 831 proteins were shared by both MHV-infected and mock samples. Two proteomic datasets were generated, taking into account the proteins that were found in at least 3 replicates of each group and that were also not shared between both groups (14 proteins for MHV and 21 for mock). A third proteomic dataset composed by 98 proteins that are shared and that present a significant difference in abundance was also generated. D) GO analysis of the three proteomic datasets that were generated using MHV-infected and mock proteomes. Only significant categories (-log P≥1.5) of interest from biological process, cellular components and molecular functions are shown. The complete results of the GO analysis can be found in Supplementary Table S3.


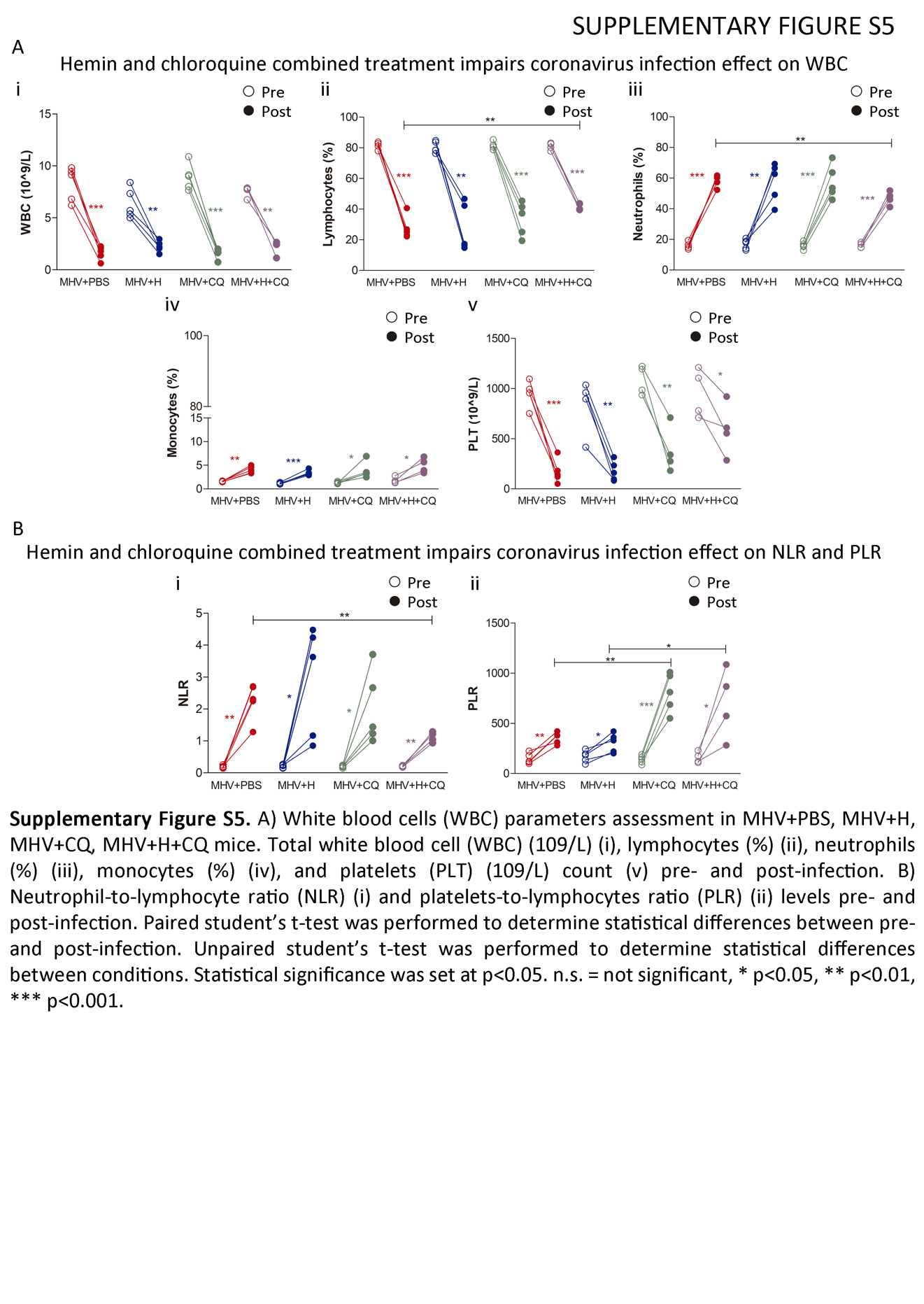


Supplementary Figure S5. A) White blood cells (WBC) parameters assessment in MHV+PBS, MHV+H, MHV+CQ and MHV+H+CQ mice. Total white blood cell (WBC) (10^9^/L) (i), lymphocytes (%) (ii), neutrophils (%) (iii), monocytes (%) (iv), and platelets (PLT) (10^9^/L) count (v) pre- and post-infection. B) Neutrophil-to-lymphocyte ratio (NLR) (i) and platelets-to-lymphocytes ratio (PLR) (ii) levels pre- and post-infection. Paired student’s t-test was performed to determine statistical differences between pre- and post-infection. Unpaired student’s t-test was performed to determine statistical differences between conditions. Statistical significance was set at p<0.05. n.s. = not significant, *p<0.05, **p<0.01, ***p<0.001.


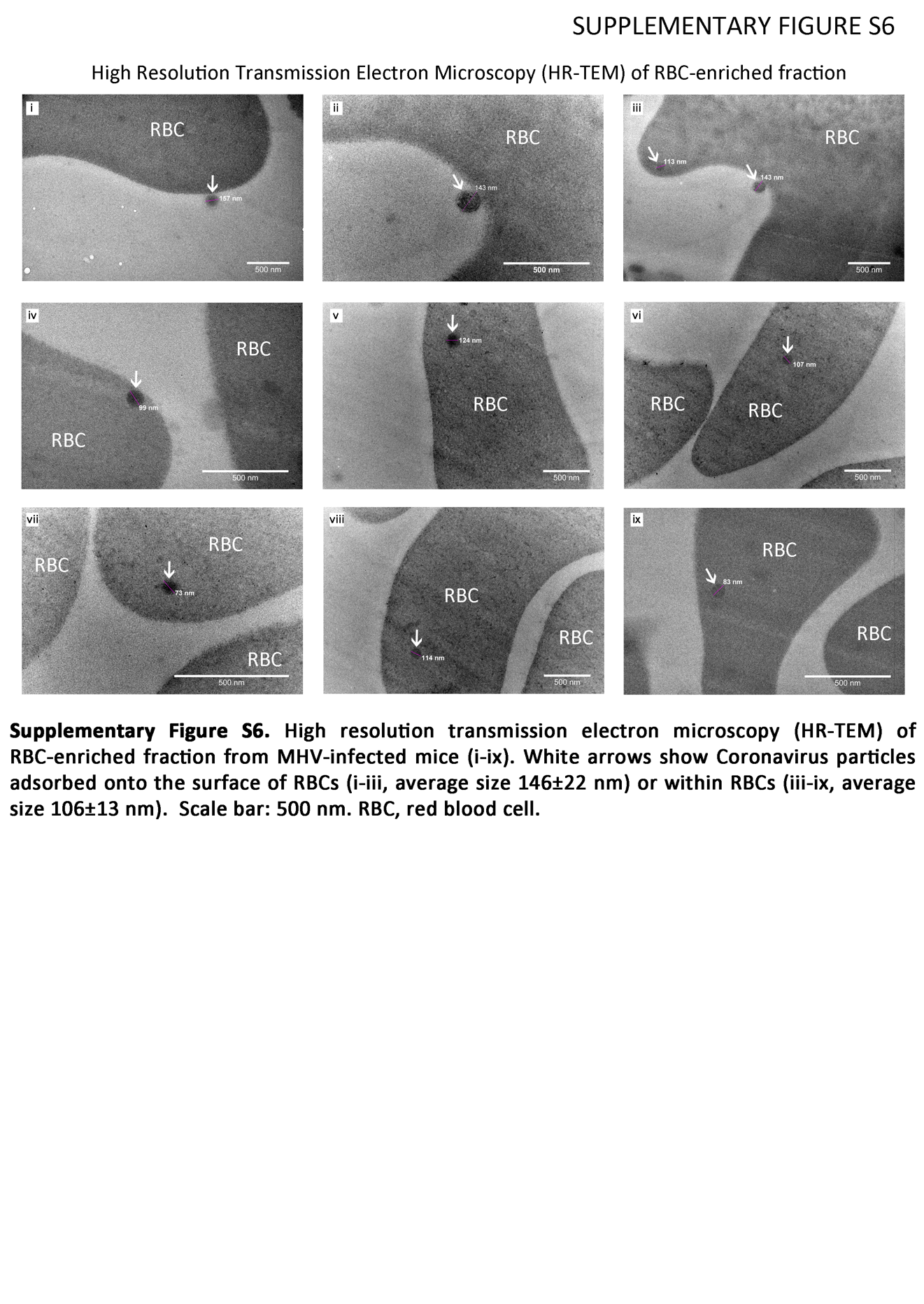


Supplementary Figure S6. High resolution transmission electron microscopy (HR-TEM) of RBC-enriched fraction from MHV-infected mice (i-ix). White arrows show Coronavirus particles adsorbed onto the surface of RBCs (i-iii, average size 146±22 nm) or within RBCs (iii-ix, average size 106± 13 nm). Scale bar: 500 nm. RBC, red blood cell.


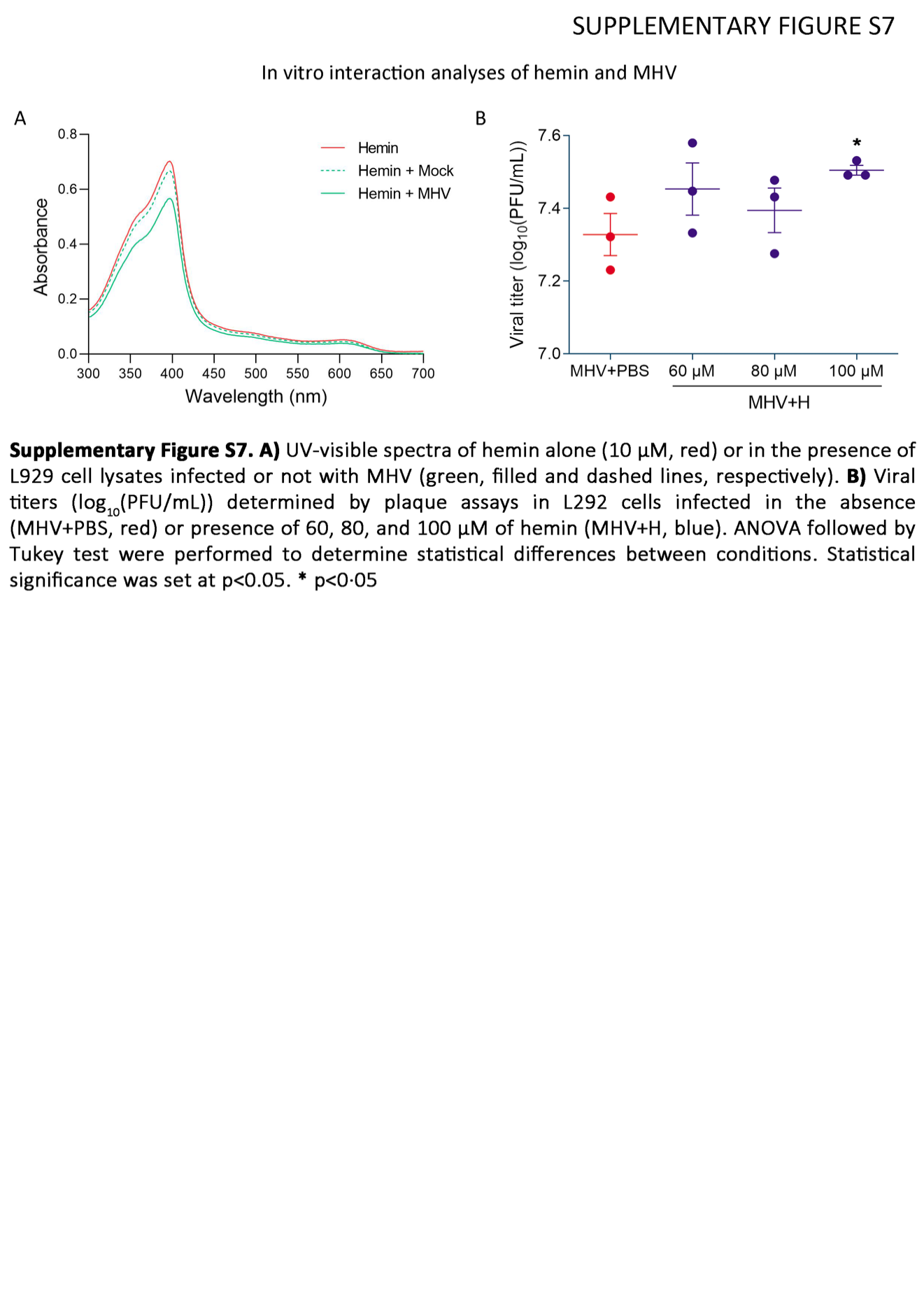
Supplementary Figure S7. A) UV-visible spectra of hemin alone (10 µM, red) or in the presence of L929 cell lysates infected or not with MHV (green, filled and dashed lines, respectively). B) Viral titers (log_10_(PFU/mL)) determined by plaque assays in L292 cells infected in the absence (MHV+PBS, red) or presence of 60, 80, and 100 µM of hemin (MHV+H, blue). ANOVA followed by Tukey test were performed to determine statistical differences between conditions. Statistical significance was set at p<0.05. * p<0.05.


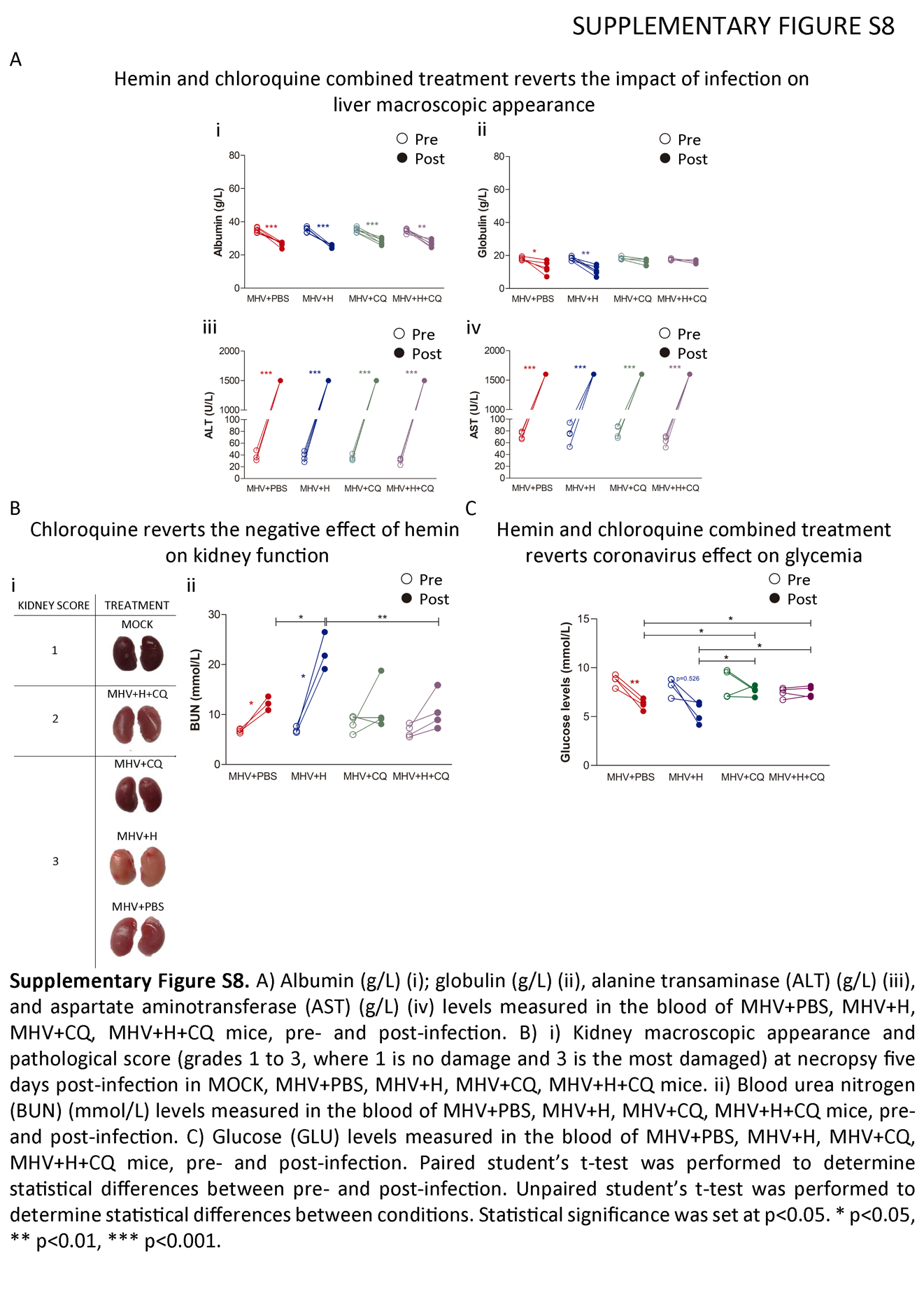


Supplementary Figure S8. A) Albumin (g/L) (i); globulin (g/L) (ii), alanine transaminase (ALT) (g/L) (iii), and aspartate aminotransferase (AST) (g/L) (iv) levels measured in the blood of MHV+PBS, MHV+H, MHV+CQ, MHV+H+CQ mice, pre- and post-infection. B) i) Kidney macroscopic appearance and pathological score (grades 1 to 3, where 1 is no damage and 3 is the most damaged) at necropsy five days post-infection in MOCK, MHV+PBS, MHV+H, MHV+CQ, MHV+H+CQ mice. ii) Blood urea nitrogen (BUN) (mmol/L) levels measured in the blood of MHV+PBS, MHV+H, MHV+CQ, MHV+H+CQ mice, pre- and post-infection. C) Glucose (GLU) levels measured in the blood of MHV+PBS, MHV+H, MHV+CQ, MHV+H+CQ mice, pre- and post-infection. Unpaired student’s t-test was performed to determine statistical differences between conditions. Statistical significance was set at p<0.05. *p<0.05, **p<0.01, ***p<0.001.


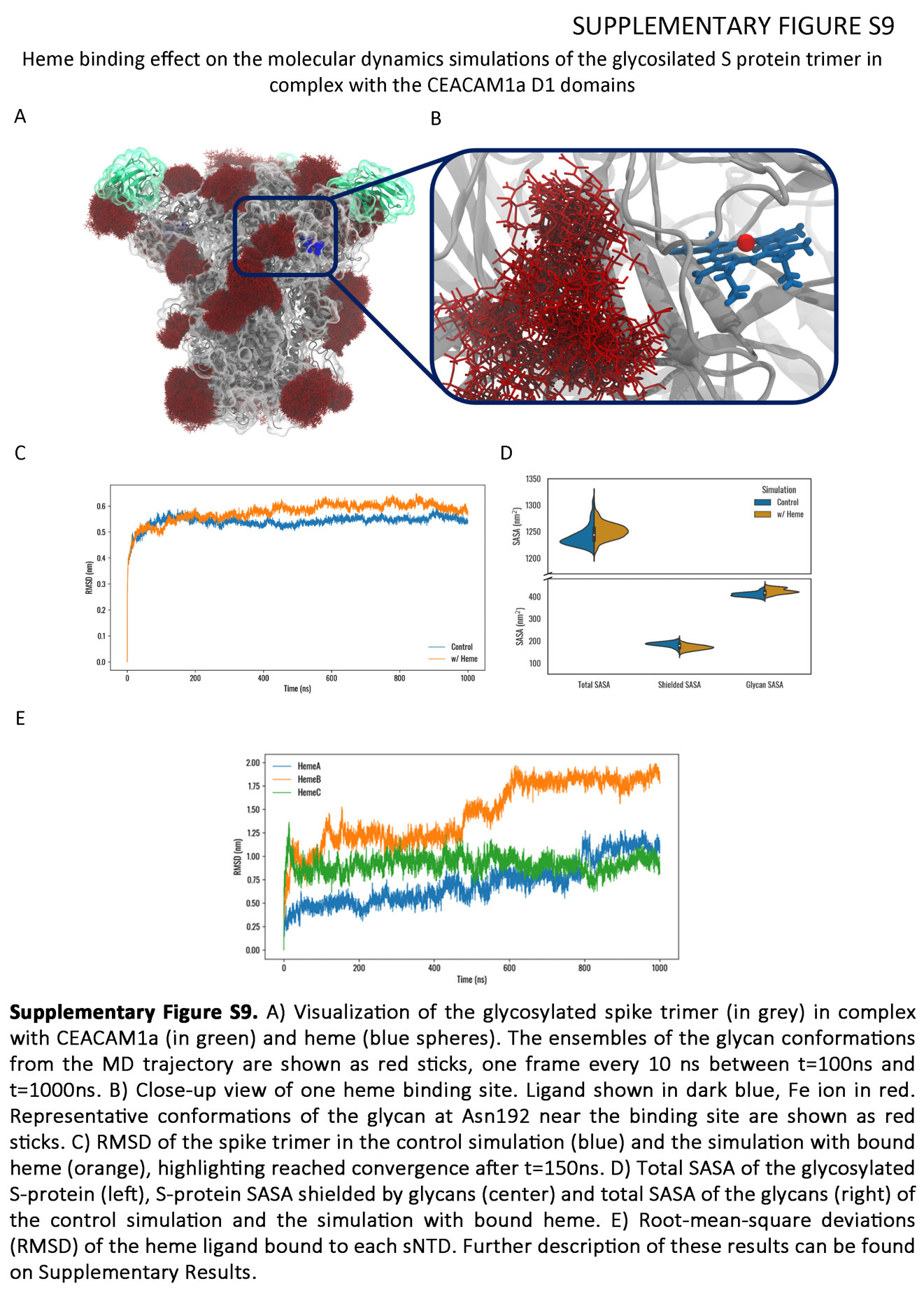


Supplementary Figure S9. A) Visualization of the glycosylated spike trimer (in grey) in complex with CEACAM1a (in green) and heme (blue spheres). The ensembles of the glycan conformations from the MD trajectory are shown as red sticks, one frame every 10 ns between t=100ns and t=1000ns. B) Close-up view of one heme binding site. Ligand shown in dark blue, Fe ion in red. Representative conformations of the glycan at Asn192 near the binding site are shown as red sticks. C) RMSD of the spike trimer in the control simulation (blue) and the simulation with bound heme (orange), highlighting reached convergence after t=150ns. D) Total SASA of the glycosylated S-protein (left), S-protein SASA shielded by glycans (center) and total SASA of the glycans (right) of the control simulation and the simulation with bound heme. E) Root-mean-square deviations (RMSD) of the heme ligand bound to each sNTD. Further description of these results can be found on Supplementary Results.

1. SUPPLEMENTARY TABLES

Table S1. Demographics of COVID-19 patients from Hospital Español (Administración de los servicios de Salud del Estado, Uruguay).

| Patients data |  |  |
| --- | --- | --- |
| Sex | Male | 19/28 (67.85 %) |
|  | Female | 9/28 (32.15 %) |
| Age | Range | 19 to 86 |
| (years) | <30 | 3 (10.72%) |
|  | 30s | 2 (7.14%) |
|  | 40s | 5 (17.86%) |
|  | 50s | 2 (7.14%) |
|  | 60s | 7 (25%) |
|  | $\geq$70 | 7 (25%) |
|  | Median | 60 |
|  | Mean | 56 |
|  | Unknown | 2 (7.14%) |

Table S2. Results of proteomic analyses from pharyngeal swabs of MHV-infected and mock mice.

Proteins with significantly different abundance in MHV vs mock samples (98)

| name | Replicate.count | Total.signal | Fold.Change | pval | gpi | botton | top |
| --- | --- | --- | --- | --- | --- | --- | --- |
| A0A087WRN7 | 12 | 756 | 3.515639122 | 0.001898 | 809 | 0.00149 | 0.00524 |
| A0A0A0MQA3 | 12 | 441 | -1.754435182 | 0.037966 | 200 | 0.002541 | 0.001449 |
| A2AEN9 | 12 | 1464 | 3.463747023 | 0.018997 | 528 | 0.003118 | 0.010798 |
| A2AEP0 | 12 | 4279 | 1.752761943 | 0.008563 | 526 | 0.014225 | 0.024934 |
| A2BHD2 | 12 | 4308 | 1.733379416 | 0.009097 | 529 | 0.01442 | 0.024995 |
| A2BHR2 | 12 | 340 | 1.68391702 | 0.008499 | 1111 | 0.00114 | 0.00192 |
| O88593 | 12 | 268 | 1.599540855 | 0.006643 | 1122 | 0.000921 | 0.001473 |
| P00687 | 12 | 3563 | 1.877454537 | 0.000633 | 531 | 0.011135 | 0.020906 |
| P00688 | 12 | 1042 | 1.805128865 | 0.000845 | 532 | 0.003348 | 0.006044 |
| P01027 | 12 | 431 | -3.171378989 | 0.015779 | 377 | 0.00297 | 0.000936 |
| P02815 | 12 | 189 | 4.212774324 | 0.000132 | 561 | 0.000332 | 0.0014 |
| P02816 | 12 | 1139 | 2.584984443 | 0.00013 | 816 | 0.002887 | 0.007463 |
| P11591 | 12 | 1124 | 1.838447016 | 0.015389 | 120 | 0.003536 | 0.006502 |
| P17897 | 12 | 291 | 2.077466335 | 0.020636 | 813 | 0.000858 | 0.001782 |
| P18242 | 12 | 361 | 1.592206738 | 0.01056 | 155 | 0.00123 | 0.001958 |
| P18761 | 12 | 1043 | 1.991637023 | 0.002282 | 544 | 0.003114 | 0.006202 |
| P22599 | 12 | 415 | -1.893650791 | 0.033578 | 199 | 0.002458 | 0.001298 |
| P49183 | 12 | 287 | 1.732240442 | 0.002234 | 354 | 0.000942 | 0.001632 |
| P63017 | 12 | 361 | -1.541638169 | 0.006323 | 114 | 0.001904 | 0.001235 |
| P97347 | 12 | 250 | -2.509376583 | 0.016511 | 1196 | 0.001517 | 0.000605 |
| P99027 | 12 | 351 | 1.648230713 | 0.000307 | 1028 | 0.001187 | 0.001956 |
| Q14AJ3 | 12 | 437 | 1.777226242 | 0.019172 | 547 | 0.001419 | 0.002522 |
| Q3KQQ2 | 12 | 406 | 1.961027761 | 0.010365 | 121 | 0.001237 | 0.002425 |
| Q5FW60 | 12 | 388 | 1.669821694 | 0.020223 | 123 | 0.001301 | 0.002173 |
| Q5SW46 | 12 | 336 | 4.126563918 | 0.015873 | 665 | 0.000618 | 0.002549 |
| Q61114 | 12 | 2989 | 1.838335502 | 0.006041 | 1197 | 0.009537 | 0.017532 |
| Q61900 | 12 | 476 | 2.034098652 | 0.000741 | 194 | 0.001409 | 0.002867 |
| Q66VB7 | 12 | 309 | 5.367282875 | 0.011414 | 1187 | 0.00046 | 0.002467 |
| Q8BND5 | 12 | 223 | 1.952374775 | 0.007831 | 126 | 0.000685 | 0.001337 |
| Q8C6C9 | 12 | 550 | 2.384085312 | 6.86E-05 | 673 | 0.001467 | 0.003497 |
| Q8CGR5 | 12 | 458 | 2.13934846 | 0.000564 | 811 | 0.00132 | 0.002824 |
| Q8JZX1 | 12 | 1406 | 2.516073457 | 0.000101 | 928 | 0.003579 | 0.009006 |
| Q8K1H9 | 12 | 1658 | 1.761226677 | 0.037473 | 188 | 0.00541 | 0.009528 |
| Q8R1E9 | 12 | 1469 | 3.522986854 | 0.000877 | 808 | 0.002901 | 0.010222 |
| Q91WB5 | 12 | 671 | 2.532230218 | 4.47E-05 | 459 | 0.001705 | 0.004318 |
| Q91XA9 | 12 | 1413 | 2.173293912 | 0.000831 | 660 | 0.004008 | 0.008711 |
| Q91Z98 | 12 | 2582 | 1.994252118 | 0.033576 | 659 | 0.007657 | 0.015271 |
| Q99M20 | 12 | 312 | 1.828931859 | 0.000559 | 221 | 0.000991 | 0.001813 |
| Q9CPP7 | 12 | 1643 | 2.340605974 | 0.005422 | 53 | 0.004465 | 0.010452 |
| Q9D3H2 | 12 | 3375 | 2.036643081 | 0.008569 | 527 | 0.010232 | 0.020839 |
| Q9JM84 | 12 | 278 | 1.78776821 | 0.003979 | 80 | 0.000908 | 0.001624 |
| A2BH64 | 12 | 167 | 1.947536096 | 4.81E-05 | 460 | 0.000507 | 0.000988 |
| O08692 | 12 | 135 | -2.145757461 | 0.006713 | 1202 | 0.000804 | 0.000375 |
| O09116 | 12 | 162 | -2.208317826 | 0.004026 | 1186 | 0.000961 | 0.000435 |
| P10639 | 12 | 112 | 1.821135335 | 0.00991 | 1190 | 0.00035 | 0.000637 |
| P26041 | 12 | 99 | -1.898929317 | 0.038156 | 205 | 0.00057 | 0.0003 |
| P56567 | 12 | 138 | 1.558849261 | 0.008575 | 678 | 0.000479 | 0.000747 |
| Q00612 | 12 | 148 | -1.993287977 | 0.004847 | 817 | 0.000856 | 0.00043 |
| Q03401 | 12 | 116 | 2.351054556 | 0.000484 | 1036 | 0.000311 | 0.000732 |
| Q3UQ05 | 12 | 113 | 2.149340703 | 0.03702 | 715 | 0.000331 | 0.000712 |
| Q58EV3 | 12 | 149 | 1.96046024 | 0.025592 | 122 | 0.000449 | 0.00088 |
| Q60648 | 12 | 155 | 1.60549835 | 0.004068 | 941 | 0.00053 | 0.00085 |
| Q61233 | 12 | 138 | -2.496395112 | 0.023263 | 668 | 0.000859 | 0.000344 |
| Q62472 | 12 | 161 | 1.710675518 | 0.027489 | 940 | 0.000537 | 0.000918 |
| Q8C1E1 | 12 | 116 | 2.239661107 | 0.003139 | 127 | 0.000323 | 0.000725 |
| Q91WR8 | 12 | 141 | 1.971885394 | 0.012096 | 203 | 0.000437 | 0.000861 |
| Q9CPU0 | 12 | 127 | 1.893059015 | 0.041651 | 166 | 0.000383 | 0.000725 |
| A8DUK4 | 11 | 2522 | -3.81973637 | 0.028138 | 97 | 0.021059 | 0.005513 |
| P00920 | 11 | 343 | -2.247605401 | 0.048328 | 716 | 0.002445 | 0.001088 |
| P02088 | 11 | 3093 | -3.58837051 | 0.030106 | 95 | 0.02544 | 0.00709 |
| P02089 | 11 | 2076 | -3.329733195 | 0.035406 | 96 | 0.016807 | 0.005048 |
| P11247 | 11 | 178 | -3.134592601 | 0.018945 | 735 | 0.001236 | 0.000394 |
| Q6JHY2 | 11 | 496 | 3.00234436 | 0.00422 | 161 | 0.001177 | 0.003533 |
| O54962 | 11 | 53 | 2.686937619 | 0.000307 | 832 | 0.000146 | 0.000393 |
| O88968 | 11 | 124 | 2.911183338 | 0.004599 | 934 | 0.0003 | 0.000874 |
| P10605 | 11 | 47 | 1.679676658 | 0.003448 | 836 | 0.000173 | 0.000291 |
| P62082 | 11 | 72 | 2.155895063 | 0.007723 | 276 | 0.000226 | 0.000488 |
| P97429 | 11 | 55 | 1.972494064 | 0.043282 | 162 | 0.000181 | 0.000358 |
| Q9JM76 | 11 | 61 | 1.598184808 | 0.015105 | 688 | 0.000229 | 0.000366 |
| P06467 | 10 | 184 | -2.922141172 | 0.0034 | 135 | 0.00166 | 0.000568 |
| Q5BKQ4 | 10 | 226 | 6.856677059 | 0.004816 | 190 | 0.000333 | 0.002282 |
| Q61495 | 10 | 144 | -7.083812731 | 0.000211 | 478 | 0.001489 | 0.00021 |
| Q6UGQ3 | 10 | 269 | 4.905608455 | 0.042549 | 176 | 0.00046 | 0.002258 |
| Q7TSF1 | 10 | 152 | -6.813179278 | 0.000216 | 477 | 0.001562 | 0.000229 |
| O08997 | 10 | 34 | 1.676432881 | 0.019949 | 1120 | 0.000138 | 0.000231 |
| P35979 | 10 | 49 | 2.515562751 | 0.007465 | 1189 | 0.000161 | 0.000405 |
| Q32M27 | 10 | 93 | 3.20627526 | 0.030558 | 1033 | 0.000214 | 0.000685 |
| Q62266 | 10 | 85 | -2.628116525 | 0.021143 | 867 | 0.000578 | 0.00022 |
| Q8VDD5 | 10 | 117 | -2.996240908 | 0.027199 | 299 | 0.00081 | 0.00027 |
| A0A0G2JDS2 | 9 | 34 | 2.141566884 | 0.030464 | 663 | 0.000122 | 0.000261 |
| P32261 | 9 | 79 | -3.187226623 | 0.017696 | 379 | 0.000687 | 0.000216 |
| P51437 | 9 | 53 | -1.835108107 | 0.028037 | 971 | 0.000388 | 0.000212 |
| P62830 | 9 | 36 | 2.073827672 | 0.02569 | 270 | 0.000144 | 0.000299 |
| P63276 | 9 | 28 | 1.980105654 | 0.045186 | 689 | 0.000115 | 0.000227 |
| Q05144 | 9 | 31 | -2.120541938 | 0.032205 | 469 | 0.000236 | 0.000111 |
| Q6ZWN5 | 9 | 54 | 2.192175767 | 0.027306 | 328 | 0.000208 | 0.000457 |
| Q7M742 | 9 | 48 | 1.912552668 | 0.04412 | 262 | 0.00019 | 0.000364 |
| Q8CGR6 | 9 | 41 | 2.126346761 | 0.041124 | 883 | 0.000149 | 0.000317 |
| Q9R1A9 | 9 | 68 | 1.738847706 | 0.021993 | 697 | 0.0003 | 0.000522 |
| P05201 | 8 | 21 | 1.717861675 | 0.000152 | 875 | 0.000106 | 0.000183 |
| P17439 | 8 | 75 | 2.537427397 | 0.01805 | 1053 | 0.000251 | 0.000637 |
| P53810 | 8 | 33 | 2.193468035 | 0.020455 | 474 | 0.000135 | 0.000297 |
| P97430 | 8 | 91 | 1.97669795 | 0.012957 | 825 | 0.000417 | 0.000824 |
| Q02819 | 8 | 23 | 1.912914786 | 0.023742 | 1128 | 0.000106 | 0.000203 |
| Q3TVK3 | 8 | 46 | 2.005731921 | 0.001612 | 286 | 0.000199 | 0.000399 |
| Q64339 | 8 | 51 | 2.15546546 | 0.011005 | 503 | 0.000221 | 0.000477 |
| Q9JII6 | 8 | 36 | 3.308376109 | 0.00301 | 834 | 9.86E-05 | 0.000326 |
| Q9JLF6 | 8 | 83 | -4.124940487 | 0.01569 | 492 | 0.000828 | 0.000201 |

Differential proteins in at least 3 MHV protein replicates (14)

| name | Replicate.count | Total.signal |
| --- | --- | --- |
| A8R0U8 | 4 | 34 |
| Q8BHN3 | 4 | 12 |
| Q3TUY3 | 3 | 10 |
| Q9JL62 | 3 | 8 |
| Q3UWK8 | 3 | 9 |
| Q8R1I1 | 3 | 6 |
| F6RR30 | 3 | 8 |
| P97805 | 3 | 6 |
| P99028 | 3 | 10 |
| Q07797 | 3 | 19 |
| P14901 | 3 | 12 |
| F6WYC8 | 3 | 19 |
| Q9D6U8 | 3 | 9 |
| Q9D7D2 | 3 | 12 |

Differential proteins in at least 3 mock replicates (21)

| name | Replicate.count | Total.signal |
| --- | --- | --- |
| P28293 | 5 | 16 |
| Q9DBB9 | 4 | 15 |
| Q9CZ30 | 4 | 8 |
| Q9R111 | 4 | 20 |
| P08032 | 4 | 81 |
| Q61096 | 4 | 22 |
| Q3UGX2 | 4 | 48 |
| P55849 | 4 | 29 |
| Q61247 | 3 | 12 |
| Q8BTM8 | 3 | 13 |
| P11835 | 3 | 20 |
| Q91Z25 | 3 | 6 |
| P62242 | 3 | 8 |
| Q8BYM1 | 3 | 49 |
| E9QP56 | 3 | 11 |
| Q9D3E6 | 3 | 20 |
| P26039 | 3 | 7 |
| Q60668 | 3 | 6 |
| A0A0R4J1N7 | 3 | 26 |
| A0A075B6A0 | 3 | 12 |
| Q9D7Q0 | 3 | 9 |

Table S3. Gene ontology classifications for biological processes (BP), cellular components (CC) and molecular functions (MF) categories enriched in the differential proteins from MHV-infected and control samples.

| ONTOLOGY | ID | Description | GeneRatio | BgRatio | pvalue | p.adjust | qvalue | geneID | Count | size |
| --- | --- | --- | --- | --- | --- | --- | --- | --- | --- | --- |
| BP | GO:0045861 | negative regulation of proteolysis | 13/127 | 368/28564 | 1.11E-08 | 2.12E-05 | 1.82E-05 | 20700/18054/13030/20701/11905/209294/20115/65019/20568/58214/238568/71907/18816 | 13 | 368 |
| BP | GO:0010466 | negative regulation of peptidase activity | 11/127 | 267/28564 | 3.31E-08 | 3.17E-05 | 2.72E-05 | 20700/18054/13030/20701/11905/209294/20568/58214/238568/71907/18816 | 11 | 267 |
| BP | GO:0051346 | negative regulation of hydrolase activity | 12/127 | 370/28564 | 1.06E-07 | 6.75E-05 | 5.79E-05 | 20700/18054/13030/20701/11905/209294/20568/58214/238568/71907/18816/11814 | 12 | 370 |
| BP | GO:0052547 | regulation of peptidase activity | 13/127 | 466/28564 | 1.73E-07 | 8.02E-05 | 6.88E-05 | 20700/18054/13030/13033/20701/11905/209294/20568/58214/238568/70186/71907/18816 | 13 | 466 |
| BP | GO:0042744 | hydrogen peroxide catabolic process | 5/127 | 30/28564 | 2.09E-07 | 8.02E-05 | 6.88E-05 | 15129/15130/15126/17523/216635 | 5 | 30 |
| BP | GO:0002181 | cytoplasmic translation | 8/127 | 146/28564 | 3.08E-07 | 9.85E-05 | 8.44E-05 | 269261/20115/65019/20068/67186/76846/20116/11991 | 8 | 146 |
| BP | GO:0015670 | carbon dioxide transport | 4/127 | 15/28564 | 4.90E-07 | 0.000134 | 0.000115 | 12349/15129/15130/15126 | 4 | 15 |
| BP | GO:1901136 | carbohydrate derivative catabolic process | 8/127 | 160/28564 | 6.20E-07 | 0.000148 | 0.000127 | 229687/21946/14466/14667/81600/104183/14544/69541 | 8 | 160 |
| BP | GO:0034101 | erythrocyte homeostasis | 8/127 | 167/28564 | 8.57E-07 | 0.000179 | 0.000153 | 15129/15130/15126/20068/14381/100038882/15368/11733 | 8 | 167 |
| BP | GO:0006026 | aminoglycan catabolic process | 5/127 | 40/28564 | 9.32E-07 | 0.000179 | 0.000153 | 229687/21946/81600/104183/69541 | 5 | 40 |
| BP | GO:0015669 | gas transport | 4/127 | 19/28564 | 1.37E-06 | 0.000239 | 0.000205 | 12349/15129/15130/15126 | 4 | 19 |
| BP | GO:0030099 | myeloid cell differentiation | 12/127 | 482/28564 | 1.73E-06 | 0.000277 | 0.000237 | 12349/15129/15130/15126/14466/14381/100038882/17886/109801/19152/192176/11733 | 12 | 482 |
| BP | GO:0048821 | erythrocyte development | 5/127 | 48/28564 | 2.36E-06 | 0.000348 | 0.000298 | 15129/15130/15126/14381/11733 | 5 | 48 |
| BP | GO:0061515 | myeloid cell development | 6/127 | 97/28564 | 4.87E-06 | 0.000667 | 0.000571 | 15129/15130/15126/14381/192176/11733 | 6 | 97 |
| BP | GO:0002262 | myeloid cell homeostasis | 8/127 | 214/28564 | 5.44E-06 | 0.000695 | 0.000596 | 15129/15130/15126/20068/14381/100038882/15368/11733 | 8 | 214 |
| BP | GO:0042743 | hydrogen peroxide metabolic process | 5/127 | 59/28564 | 6.63E-06 | 0.000794 | 0.000681 | 15129/15130/15126/17523/216635 | 5 | 59 |
| BP | GO:0042742 | defense response to bacterium | 10/127 | 386/28564 | 9.30E-06 | 0.001049 | 0.000899 | 21946/17523/17110/12796/20568/76113/100038882/13035/16019/69541 | 10 | 386 |
| BP | GO:0048872 | homeostasis of number of cells | 10/127 | 391/28564 | 1.04E-05 | 0.001107 | 0.000949 | 15129/15130/15126/14466/20068/14381/100038882/15368/20739/11733 | 10 | 391 |
| BP | GO:0002444 | myeloid leukocyte mediated immunity | 6/127 | 121/28564 | 1.74E-05 | 0.001752 | 0.001501 | 12266/13419/19354/15368/13035/16414 | 6 | 121 |
| BP | GO:1901072 | glucosamine-containing compound catabolic process | 3/127 | 12/28564 | 1.83E-05 | 0.001758 | 0.001507 | 229687/81600/104183 | 3 | 12 |
| BP | GO:0002886 | regulation of myeloid leukocyte mediated immunity | 5/127 | 76/28564 | 2.30E-05 | 0.002103 | 0.001802 | 12266/13419/19354/15368/16414 | 5 | 76 |
| BP | GO:0042119 | neutrophil activation | 4/127 | 41/28564 | 3.32E-05 | 0.002895 | 0.002482 | 13419/12796/13035/16414 | 4 | 41 |
| BP | GO:0019755 | one-carbon compound transport | 4/127 | 42/28564 | 3.66E-05 | 0.00305 | 0.002614 | 12349/15129/15130/15126 | 4 | 42 |
| BP | GO:0006778 | porphyrin-containing compound metabolic process | 4/127 | 43/28564 | 4.02E-05 | 0.003212 | 0.002753 | 15368/20739/20741/11733 | 4 | 43 |
| BP | GO:0018149 | peptide cross-linking | 4/127 | 45/28564 | 4.82E-05 | 0.003697 | 0.003169 | 20766/209294/20753/21816 | 4 | 45 |
| BP | GO:0036230 | granulocyte activation | 4/127 | 47/28564 | 5.73E-05 | 0.004227 | 0.003623 | 13419/12796/13035/16414 | 4 | 47 |
| BP | GO:0030218 | erythrocyte differentiation | 6/127 | 155/28564 | 6.98E-05 | 0.004953 | 0.004246 | 15129/15130/15126/14381/100038882/11733 | 6 | 155 |
| BP | GO:0035821 | modulation of process of another organism | 3/127 | 19/28564 | 7.90E-05 | 0.005219 | 0.004473 | 12266/12796/20568 | 3 | 19 |
| BP | GO:0046348 | amino sugar catabolic process | 3/127 | 19/28564 | 7.90E-05 | 0.005219 | 0.004473 | 229687/81600/104183 | 3 | 19 |
| BP | GO:0033013 | tetrapyrrole metabolic process | 4/127 | 53/28564 | 9.22E-05 | 0.005891 | 0.005049 | 15368/20739/20741/11733 | 4 | 53 |
| BP | GO:0072593 | reactive oxygen species metabolic process | 7/127 | 249/28564 | 0.000129262 | 0.007993 | 0.006852 | 15129/15130/15126/17523/14381/16414/216635 | 7 | 249 |
| BP | GO:0043254 | regulation of protein-containing complex assembly | 9/127 | 435/28564 | 0.000147572 | 0.00884 | 0.007578 | 14466/17698/18826/100038882/56378/14544/20739/20741/11867 | 9 | 435 |
| BP | GO:0007015 | actin filament organization | 9/127 | 454/28564 | 0.000202789 | 0.01178 | 0.010097 | 19354/18826/17886/56378/20739/20741/18816/192176/11867 | 9 | 454 |
| BP | GO:0006022 | aminoglycan metabolic process | 5/127 | 121/28564 | 0.000211344 | 0.011916 | 0.010214 | 229687/21946/81600/104183/69541 | 5 | 121 |
| BP | GO:0010951 | negative regulation of endopeptidase activity | 6/127 | 193/28564 | 0.000231151 | 0.012365 | 0.010599 | 20700/20701/58214/238568/71907/18816 | 6 | 193 |
| BP | GO:1901071 | glucosamine-containing compound metabolic process | 3/127 | 27/28564 | 0.000232209 | 0.012365 | 0.010599 | 229687/81600/104183 | 3 | 27 |
| BP | GO:0052548 | regulation of endopeptidase activity | 8/127 | 368/28564 | 0.000249114 | 0.012907 | 0.011063 | 20700/13033/20701/58214/238568/70186/71907/18816 | 8 | 368 |
| BP | GO:0042274 | ribosomal small subunit biogenesis | 4/127 | 74/28564 | 0.000337101 | 0.016881 | 0.01447 | 20115/20068/76846/20116 | 4 | 74 |
| BP | GO:0006779 | porphyrin-containing compound biosynthetic process | 3/127 | 31/28564 | 0.000352245 | 0.016881 | 0.01447 | 20739/20741/11733 | 3 | 31 |
| BP | GO:0033014 | tetrapyrrole biosynthetic process | 3/127 | 31/28564 | 0.000352245 | 0.016881 | 0.01447 | 20739/20741/11733 | 3 | 31 |
| BP | GO:1902903 | regulation of supramolecular fiber organization | 8/127 | 394/28564 | 0.000392855 | 0.018368 | 0.015744 | 15481/56378/14544/20739/20741/18816/192176/11867 | 8 | 394 |
| BP | GO:0045745 | positive regulation of G protein-coupled receptor signaling pathway | 3/127 | 33/28564 | 0.000424788 | 0.019389 | 0.016619 | 12266/317653/17886 | 3 | 33 |
| BP | GO:0006979 | response to oxidative stress | 8/127 | 408/28564 | 0.000494596 | 0.02205 | 0.0189 | 11927/23825/22166/17523/14381/76113/75512/15368 | 8 | 408 |
| BP | GO:0031330 | negative regulation of cellular catabolic process | 6/127 | 240/28564 | 0.000732984 | 0.031787 | 0.027246 | 20115/65019/104009/15368/11814/11991 | 6 | 240 |
| BP | GO:0009895 | negative regulation of catabolic process | 7/127 | 336/28564 | 0.000783912 | 0.031787 | 0.027246 | 20115/65019/104009/15368/192176/11814/11991 | 7 | 336 |
| BP | GO:0016125 | sterol metabolic process | 5/127 | 162/28564 | 0.000803709 | 0.031787 | 0.027246 | 14466/14381/628236/329055/11814 | 5 | 162 |
| BP | GO:0002446 | neutrophil mediated immunity | 3/127 | 41/28564 | 0.000808717 | 0.031787 | 0.027246 | 13419/13035/16414 | 3 | 41 |
| BP | GO:1900003 | regulation of serine-type endopeptidase activity | 2/127 | 10/28564 | 0.000862232 | 0.031787 | 0.027246 | 20700/20701 | 2 | 10 |
| BP | GO:1900004 | negative regulation of serine-type endopeptidase activity | 2/127 | 10/28564 | 0.000862232 | 0.031787 | 0.027246 | 20700/20701 | 2 | 10 |
| BP | GO:1902571 | regulation of serine-type peptidase activity | 2/127 | 10/28564 | 0.000862232 | 0.031787 | 0.027246 | 20700/20701 | 2 | 10 |
| BP | GO:1902572 | negative regulation of serine-type peptidase activity | 2/127 | 10/28564 | 0.000862232 | 0.031787 | 0.027246 | 20700/20701 | 2 | 10 |
| BP | GO:1904667 | negative regulation of ubiquitin protein ligase activity | 2/127 | 10/28564 | 0.000862232 | 0.031787 | 0.027246 | 20115/65019 | 2 | 10 |
| BP | GO:0022411 | cellular component disassembly | 8/127 | 450/28564 | 0.000934211 | 0.03379 | 0.028963 | 12266/14466/65019/15481/18826/13035/20739/20741 | 8 | 450 |
| BP | GO:0045022 | early endosome to late endosome transport | 3/127 | 44/28564 | 0.000995041 | 0.035324 | 0.030278 | 17698/192176/16019 | 3 | 44 |
| BP | GO:0007229 | integrin-mediated signaling pathway | 4/127 | 102/28564 | 0.001131422 | 0.038109 | 0.032665 | 100038882/192176/16414/21894 | 4 | 102 |
| BP | GO:0006040 | amino sugar metabolic process | 3/127 | 46/28564 | 0.00113314 | 0.038109 | 0.032665 | 229687/81600/104183 | 3 | 46 |
| BP | GO:0098927 | vesicle-mediated transport between endosomal compartments | 3/127 | 46/28564 | 0.00113314 | 0.038109 | 0.032665 | 17698/192176/16019 | 3 | 46 |
| BP | GO:0050830 | defense response to Gram-positive bacterium | 5/127 | 176/28564 | 0.001163743 | 0.038423 | 0.032934 | 21946/17110/12796/13035/69541 | 5 | 176 |
| BP | GO:0002274 | myeloid leukocyte activation | 6/127 | 264/28564 | 0.001198202 | 0.038423 | 0.032934 | 13419/12796/19354/15368/13035/16414 | 6 | 264 |
| BP | GO:0140236 | translation at presynapse | 3/127 | 47/28564 | 0.001206491 | 0.038423 | 0.032934 | 269261/65019/67186 | 3 | 47 |
| BP | GO:0071803 | positive regulation of podosome assembly | 2/127 | 12/28564 | 0.00125726 | 0.038423 | 0.032934 | 17698/18826 | 2 | 12 |
| BP | GO:0001580 | detection of chemical stimulus involved in sensory perception of bitter taste | 3/127 | 48/28564 | 0.001282769 | 0.038423 | 0.032934 | 18716/12353/76113 | 3 | 48 |
| BP | GO:0140241 | translation at synapse | 3/127 | 48/28564 | 0.001282769 | 0.038423 | 0.032934 | 269261/65019/67186 | 3 | 48 |
| BP | GO:0140242 | translation at postsynapse | 3/127 | 48/28564 | 0.001282769 | 0.038423 | 0.032934 | 269261/65019/67186 | 3 | 48 |
| BP | GO:0019730 | antimicrobial humoral response | 5/127 | 181/28564 | 0.001317477 | 0.038488 | 0.03299 | 21946/12796/20568/13035/16019 | 5 | 181 |
| BP | GO:0007596 | blood coagulation | 5/127 | 183/28564 | 0.001382989 | 0.038488 | 0.03299 | 12266/11905/13035/18816/192176 | 5 | 183 |
| BP | GO:0002275 | myeloid cell activation involved in immune response | 4/127 | 108/28564 | 0.001397935 | 0.038488 | 0.03299 | 13419/19354/15368/16414 | 4 | 108 |
| BP | GO:0019731 | antibacterial humoral response | 4/127 | 108/28564 | 0.001397935 | 0.038488 | 0.03299 | 12796/20568/13035/16019 | 4 | 108 |
| BP | GO:0002443 | leukocyte mediated immunity | 8/127 | 480/28564 | 0.001407841 | 0.038488 | 0.03299 | 12266/14466/13419/19354/15368/13035/16414/16019 | 8 | 480 |
| BP | GO:0046364 | monosaccharide biosynthetic process | 4/127 | 109/28564 | 0.001446253 | 0.038488 | 0.03299 | 14718/14381/227627/58810 | 4 | 109 |
| BP | GO:0006122 | mitochondrial electron transport, ubiquinol to cytochrome c | 2/127 | 13/28564 | 0.001481533 | 0.038488 | 0.03299 | 66152/66576 | 2 | 13 |
| BP | GO:0007597 | blood coagulation, intrinsic pathway | 2/127 | 13/28564 | 0.001481533 | 0.038488 | 0.03299 | 11905/192176 | 2 | 13 |
| BP | GO:0007599 | hemostasis | 5/127 | 186/28564 | 0.001485722 | 0.038488 | 0.03299 | 12266/11905/13035/18816/192176 | 5 | 186 |
| BP | GO:0050817 | coagulation | 5/127 | 186/28564 | 0.001485722 | 0.038488 | 0.03299 | 12266/11905/13035/18816/192176 | 5 | 186 |
| BP | GO:0110053 | regulation of actin filament organization | 6/127 | 280/28564 | 0.00161572 | 0.040282 | 0.034528 | 56378/20739/20741/18816/192176/11867 | 6 | 280 |
| BP | GO:0050912 | detection of chemical stimulus involved in sensory perception of taste | 3/127 | 52/28564 | 0.001618007 | 0.040282 | 0.034528 | 18716/12353/76113 | 3 | 52 |
| BP | GO:0050913 | sensory perception of bitter taste | 3/127 | 52/28564 | 0.001618007 | 0.040282 | 0.034528 | 18716/12353/76113 | 3 | 52 |
| BP | GO:1990748 | cellular detoxification | 3/127 | 53/28564 | 0.001709556 | 0.04182 | 0.035846 | 17523/76113/58810 | 3 | 53 |
| BP | GO:0046479 | glycosphingolipid catabolic process | 2/127 | 14/28564 | 0.001723431 | 0.04182 | 0.035846 | 14466/14667 | 2 | 14 |
| BP | GO:0002526 | acute inflammatory response | 4/127 | 116/28564 | 0.001817563 | 0.043553 | 0.037332 | 12266/20701/13419/18816 | 4 | 116 |
| BP | GO:0050821 | protein stabilization | 5/127 | 196/28564 | 0.001868991 | 0.044233 | 0.037914 | 20115/65019/13510/225256/192176 | 5 | 196 |
| BP | GO:0042060 | wound healing | 7/127 | 392/28564 | 0.001898426 | 0.044381 | 0.038042 | 12266/11905/17886/15368/13035/18816/192176 | 7 | 392 |
| BP | GO:0010288 | response to lead ion | 2/127 | 15/28564 | 0.001982796 | 0.045795 | 0.039254 | 20700/20701 | 2 | 15 |
| BP | GO:0043300 | regulation of leukocyte degranulation | 3/127 | 57/28564 | 0.002107903 | 0.047814 | 0.040984 | 19354/15368/16414 | 3 | 57 |
| BP | GO:0032963 | collagen metabolic process | 4/127 | 121/28564 | 0.002120098 | 0.047814 | 0.040984 | 14718/13030/19152/18816 | 4 | 121 |
| BP | GO:0006959 | humoral immune response | 6/127 | 299/28564 | 0.002245879 | 0.048485 | 0.041559 | 21946/12266/12796/20568/13035/16019 | 6 | 299 |
| BP | GO:0019377 | glycolipid catabolic process | 2/127 | 16/28564 | 0.00225947 | 0.048485 | 0.041559 | 14466/14667 | 2 | 16 |
| BP | GO:0046185 | aldehyde catabolic process | 2/127 | 16/28564 | 0.00225947 | 0.048485 | 0.041559 | 109801/58810 | 2 | 16 |
| BP | GO:0071801 | regulation of podosome assembly | 2/127 | 16/28564 | 0.00225947 | 0.048485 | 0.041559 | 17698/18826 | 2 | 16 |
| BP | GO:0032271 | regulation of protein polymerization | 5/127 | 206/28564 | 0.002320131 | 0.048485 | 0.041559 | 56378/14544/20739/20741/11867 | 5 | 206 |
| BP | GO:0031640 | killing of cells of another organism | 3/127 | 59/28564 | 0.002326897 | 0.048485 | 0.041559 | 21946/12266/17110 | 3 | 59 |
| BP | GO:0050832 | defense response to fungus | 3/127 | 59/28564 | 0.002326897 | 0.048485 | 0.041559 | 17523/12796/13035 | 3 | 59 |
| CC | GO:0005833 | hemoglobin complex | 5/130 | 13/28585 | 2.25E-09 | 3.68E-07 | 2.88E-07 | 100503605/15129/15130/15126/216635 | 5 | 13 |
| CC | GO:0031838 | haptoglobin-hemoglobin complex | 5/130 | 14/28585 | 3.49E-09 | 3.68E-07 | 2.88E-07 | 100503605/15129/15130/15126/216635 | 5 | 14 |
| CC | GO:0022626 | cytosolic ribosome | 7/130 | 111/28585 | 7.84E-07 | 5.52E-05 | 4.32E-05 | 269261/20115/65019/20068/67186/76846/20116 | 7 | 111 |
| CC | GO:0009898 | cytoplasmic side of plasma membrane | 8/130 | 178/28585 | 1.64E-06 | 8.67E-05 | 6.79E-05 | 17698/14381/14667/13510/225256/17886/20739/11733 | 8 | 178 |
| CC | GO:0098562 | cytoplasmic side of membrane | 8/130 | 205/28585 | 4.69E-06 | 0.000198 | 0.000155 | 17698/14381/14667/13510/225256/17886/20739/11733 | 8 | 205 |
| CC | GO:0044391 | ribosomal subunit | 7/130 | 201/28585 | 3.90E-05 | 0.001371 | 0.001073 | 269261/20115/65019/20068/67186/76846/20116 | 7 | 201 |
| CC | GO:0022627 | cytosolic small ribosomal subunit | 4/130 | 44/28585 | 4.82E-05 | 0.001451 | 0.001137 | 20115/20068/76846/20116 | 4 | 44 |
| CC | GO:0005840 | ribosome | 7/130 | 236/28585 | 0.000107 | 0.002818 | 0.002207 | 269261/20115/65019/20068/67186/76846/20116 | 7 | 236 |
| CC | GO:0005775 | vacuolar lumen | 3/130 | 24/28585 | 0.000173 | 0.00366 | 0.002867 | 14466/15481/19152 | 3 | 24 |
| CC | GO:1904090 | peptidase inhibitor complex | 3/130 | 24/28585 | 0.000173 | 0.00366 | 0.002867 | 13030/209294/238568 | 3 | 24 |
| CC | GO:0030057 | desmosome | 3/130 | 25/28585 | 0.000196 | 0.003768 | 0.002952 | 13510/225256/13505 | 3 | 25 |
| CC | GO:0030863 | cortical cytoskeleton | 5/130 | 119/28585 | 0.000217 | 0.00382 | 0.002992 | 17886/20739/20741/192176/11733 | 5 | 119 |
| CC | GO:0005884 | actin filament | 5/130 | 128/28585 | 0.000305 | 0.004945 | 0.003873 | 19354/18826/17886/56378/192176 | 5 | 128 |
| CC | GO:0030141 | secretory granule | 9/130 | 485/28585 | 0.000389 | 0.005866 | 0.004594 | 17523/17110/17698/12796/11571/626834/17886/13035/19152 | 9 | 485 |
| CC | GO:0015935 | small ribosomal subunit | 4/130 | 81/28585 | 0.000518 | 0.007291 | 0.00571 | 20115/20068/76846/20116 | 4 | 81 |
| CC | GO:0016324 | apical plasma membrane | 8/130 | 406/28585 | 0.000557 | 0.007341 | 0.00575 | 18716/13030/17698/11746/13510/225256/17886/58810 | 8 | 406 |
| CC | GO:0001931 | uropod | 2/130 | 11/28585 | 0.001099 | 0.01261 | 0.009877 | 17698/17886 | 2 | 11 |
| CC | GO:0031904 | endosome lumen | 2/130 | 11/28585 | 0.001099 | 0.01261 | 0.009877 | 13033/15481 | 2 | 11 |
| CC | GO:0005925 | focal adhesion | 5/130 | 174/28585 | 0.001223 | 0.01261 | 0.009877 | 17698/18826/17886/16414/21894 | 5 | 174 |
| CC | GO:0005750 | mitochondrial respiratory chain complex III | 2/130 | 12/28585 | 0.001315 | 0.01261 | 0.009877 | 66152/66576 | 2 | 12 |
| CC | GO:0031254 | cell trailing edge | 2/130 | 12/28585 | 0.001315 | 0.01261 | 0.009877 | 17698/17886 | 2 | 12 |
| CC | GO:0045275 | respiratory chain complex III | 2/130 | 12/28585 | 0.001315 | 0.01261 | 0.009877 | 66152/66576 | 2 | 12 |
| CC | GO:0030055 | cell-substrate junction | 5/130 | 188/28585 | 0.00172 | 0.015783 | 0.012362 | 17698/18826/17886/16414/21894 | 5 | 188 |
| CC | GO:0042581 | specific granule | 2/130 | 14/28585 | 0.001802 | 0.015843 | 0.012409 | 12796/11571 | 2 | 14 |
| CC | GO:0015629 | actin cytoskeleton | 8/130 | 500/28585 | 0.002096 | 0.017024 | 0.013334 | 19354/18826/17886/56378/20739/20741/192176/11867 | 8 | 500 |
| CC | GO:0062023 | collagen-containing extracellular matrix | 7/130 | 390/28585 | 0.002098 | 0.017024 | 0.013334 | 20700/13030/13033/11905/11746/71756/18816 | 7 | 390 |
| CC | GO:0001533 | cornified envelope | 3/130 | 62/28585 | 0.002858 | 0.022334 | 0.017493 | 209294/20129/20753 | 3 | 62 |
| CC | GO:0043209 | myelin sheath | 5/130 | 218/28585 | 0.003263 | 0.023387 | 0.018317 | 12349/15129/17698/18738/15481 | 5 | 218 |
| CC | GO:0022625 | cytosolic large ribosomal subunit | 3/130 | 65/28585 | 0.003268 | 0.023387 | 0.018317 | 269261/65019/67186 | 3 | 65 |
| CC | GO:0044853 | plasma membrane raft | 4/130 | 134/28585 | 0.003325 | 0.023387 | 0.018317 | 13030/15368/19152/16414 | 4 | 134 |
| CC | GO:0043202 | lysosomal lumen | 2/130 | 20/28585 | 0.003696 | 0.025156 | 0.019703 | 14466/15481 | 2 | 20 |
| CC | GO:0016328 | lateral plasma membrane | 3/130 | 72/28585 | 0.004361 | 0.027721 | 0.021712 | 13510/225256/17886 | 3 | 72 |
| CC | GO:0005766 | primary lysosome | 2/130 | 22/28585 | 0.004467 | 0.027721 | 0.021712 | 17523/19152 | 2 | 22 |
| CC | GO:0042582 | azurophil granule | 2/130 | 22/28585 | 0.004467 | 0.027721 | 0.021712 | 17523/19152 | 2 | 22 |
| CC | GO:0005912 | adherens junction | 4/130 | 153/28585 | 0.005321 | 0.032076 | 0.025123 | 17698/17886/21816/21894 | 4 | 153 |
| CC | GO:0030864 | cortical actin cytoskeleton | 3/130 | 84/28585 | 0.006697 | 0.039254 | 0.030746 | 17886/20739/20741 | 3 | 84 |
| MF | GO:0004866 | endopeptidase inhibitor activity | 12/130 | 205/28171 | 2.17E-10 | 2.08E-08 | 1.46E-08 | 20700/18054/12266/20701/11905/209294/20568/20599/58214/238568/71907/18816 | 12 | 205 |
| MF | GO:0004601 | peroxidase activity | 8/130 | 60/28171 | 3.46E-10 | 2.08E-08 | 1.46E-08 | 100503605/15129/15130/15126/17523/76113/75512/216635 | 8 | 60 |
| MF | GO:0030414 | peptidase inhibitor activity | 12/130 | 217/28171 | 4.18E-10 | 2.08E-08 | 1.46E-08 | 20700/18054/12266/20701/11905/209294/20568/20599/58214/238568/71907/18816 | 12 | 217 |
| MF | GO:0004857 | enzyme inhibitor activity | 15/130 | 392/28171 | 4.30E-10 | 2.08E-08 | 1.46E-08 | 20700/18054/12266/20701/11905/209294/20115/65019/20568/20599/58214/238568/71907/18816/11814 | 15 | 392 |
| MF | GO:0016684 | oxidoreductase activity, acting on peroxide as acceptor | 8/130 | 62/28171 | 4.54E-10 | 2.08E-08 | 1.46E-08 | 100503605/15129/15130/15126/17523/76113/75512/216635 | 8 | 62 |
| MF | GO:0061135 | endopeptidase regulator activity | 12/130 | 221/28171 | 5.15E-10 | 2.08E-08 | 1.46E-08 | 20700/18054/12266/20701/11905/209294/20568/20599/58214/238568/71907/18816 | 12 | 221 |
| MF | GO:0004553 | hydrolase activity, hydrolyzing O-glycosyl compounds | 9/130 | 103/28171 | 1.25E-09 | 4.32E-08 | 3.04E-08 | 229687/11722/109959/14466/17110/14667/81600/14376/69541 | 9 | 103 |
| MF | GO:0031720 | haptoglobin binding | 5/130 | 13/28171 | 2.42E-09 | 7.32E-08 | 5.16E-08 | 100503605/15129/15130/15126/216635 | 5 | 13 |
| MF | GO:0061134 | peptidase regulator activity | 12/130 | 276/28171 | 6.37E-09 | 1.71E-07 | 1.21E-07 | 20700/18054/12266/20701/11905/209294/20568/20599/58214/238568/71907/18816 | 12 | 276 |
| MF | GO:0016209 | antioxidant activity | 8/130 | 88/28171 | 7.80E-09 | 1.89E-07 | 1.33E-07 | 100503605/15129/15130/15126/17523/76113/75512/216635 | 8 | 88 |
| MF | GO:0016798 | hydrolase activity, acting on glycosyl bonds | 9/130 | 137/28171 | 1.56E-08 | 3.44E-07 | 2.42E-07 | 229687/11722/109959/14466/17110/14667/81600/14376/69541 | 9 | 137 |
| MF | GO:0019825 | oxygen binding | 5/130 | 27/28171 | 1.44E-07 | 2.91E-06 | 2.05E-06 | 100503605/15129/15130/15126/216635 | 5 | 27 |
| MF | GO:0005344 | oxygen carrier activity | 4/130 | 14/28171 | 4.18E-07 | 7.78E-06 | 5.48E-06 | 15129/15130/15126/216635 | 4 | 14 |
| MF | GO:0046906 | tetrapyrrole binding | 8/130 | 169/28171 | 1.24E-06 | 2.14E-05 | 1.51E-05 | 21452/15129/15130/15126/17523/76113/15368/216635 | 8 | 169 |
| MF | GO:0004867 | serine-type endopeptidase inhibitor activity | 7/130 | 119/28171 | 1.38E-06 | 2.23E-05 | 1.57E-05 | 20700/20701/11905/20568/238568/71907/18816 | 7 | 119 |
| MF | GO:0140104 | molecular carrier activity | 6/130 | 88/28171 | 3.42E-06 | 5.17E-05 | 3.64E-05 | 11927/21452/15129/15130/15126/216635 | 6 | 88 |
| MF | GO:0051015 | actin filament binding | 8/130 | 221/28171 | 9.04E-06 | 0.000129 | 9.06E-05 | 18826/17886/56378/20739/20741/192176/11867/21894 | 8 | 221 |
| MF | GO:0020037 | heme binding | 7/130 | 160/28171 | 9.84E-06 | 0.000132 | 9.32E-05 | 15129/15130/15126/17523/76113/15368/216635 | 7 | 160 |
| MF | GO:0003735 | structural constituent of ribosome | 7/130 | 167/28171 | 1.30E-05 | 0.000166 | 0.000117 | 269261/20115/65019/20068/67186/76846/20116 | 7 | 167 |
| MF | GO:0008061 | chitin binding | 3/130 | 11/28171 | 1.54E-05 | 0.000187 | 0.000131 | 229687/81600/104183 | 3 | 11 |
| MF | GO:0030492 | hemoglobin binding | 3/130 | 13/28171 | 2.65E-05 | 0.000306 | 0.000216 | 100503605/15129/15130 | 3 | 13 |
| MF | GO:0002020 | protease binding | 7/130 | 192/28171 | 3.19E-05 | 0.000351 | 0.000247 | 20700/20701/11905/209294/238568/13035/18816 | 7 | 192 |
| MF | GO:0061783 | peptidoglycan muralytic activity | 3/130 | 15/28171 | 4.20E-05 | 0.000441 | 0.000311 | 21946/17110/69541 | 3 | 15 |
| MF | GO:0003779 | actin binding | 10/130 | 447/28171 | 4.48E-05 | 0.000452 | 0.000318 | 17698/13419/18826/17886/56378/20739/20741/192176/11867/21894 | 10 | 447 |
| MF | GO:0005550 | pheromone binding | 5/130 | 93/28171 | 7.27E-05 | 0.000704 | 0.000496 | 17844/16820/17842/381530/16821 | 5 | 93 |
| MF | GO:0016298 | lipase activity | 5/130 | 127/28171 | 0.000314 | 0.002923 | 0.002059 | 18946/67717/628236/15368/329055 | 5 | 127 |
| MF | GO:0005200 | structural constituent of cytoskeleton | 4/130 | 72/28171 | 0.000349 | 0.003132 | 0.002207 | 56378/20741/11867/21894 | 4 | 72 |
| MF | GO:0005178 | integrin binding | 5/130 | 154/28171 | 0.000756 | 0.006536 | 0.004606 | 18826/100038882/17886/16414/21894 | 5 | 154 |
| MF | GO:0043177 | organic acid binding | 6/130 | 236/28171 | 0.000815 | 0.006802 | 0.004793 | 15129/15130/14718/15126/18738/216635 | 6 | 236 |
| MF | GO:1990948 | ubiquitin ligase inhibitor activity | 2/130 | 10/28171 | 0.000928 | 0.007487 | 0.005276 | 20115/65019 | 2 | 10 |
| MF | GO:0052689 | carboxylic ester hydrolase activity | 5/130 | 165/28171 | 0.00103 | 0.008042 | 0.005667 | 12349/18946/67717/628236/329055 | 5 | 165 |
| MF | GO:0003796 | lysozyme activity | 2/130 | 11/28171 | 0.001131 | 0.008294 | 0.005844 | 17110/69541 | 2 | 11 |
| MF | GO:0055105 | ubiquitin-protein transferase inhibitor activity | 2/130 | 11/28171 | 0.001131 | 0.008294 | 0.005844 | 20115/65019 | 2 | 11 |
| MF | GO:0015926 | glucosidase activity | 2/130 | 13/28171 | 0.001594 | 0.011348 | 0.007996 | 14466/14376 | 2 | 13 |
| MF | GO:0004089 | carbonate dehydratase activity | 2/130 | 15/28171 | 0.002133 | 0.013953 | 0.009832 | 12349/12353 | 2 | 15 |
| MF | GO:0031404 | chloride ion binding | 2/130 | 15/28171 | 0.002133 | 0.013953 | 0.009832 | 11722/109959 | 2 | 15 |
| MF | GO:0045295 | gamma-catenin binding | 2/130 | 15/28171 | 0.002133 | 0.013953 | 0.009832 | 13510/225256 | 2 | 15 |
| MF | GO:0034987 | immunoglobulin receptor binding | 2/130 | 16/28171 | 0.002431 | 0.015479 | 0.010908 | 192176/16019 | 2 | 16 |
| MF | GO:0042834 | peptidoglycan binding | 2/130 | 17/28171 | 0.002746 | 0.017042 | 0.012009 | 21946/16019 | 2 | 17 |
| MF | GO:0050839 | cell adhesion molecule binding | 6/130 | 305/28171 | 0.002981 | 0.017749 | 0.012507 | 17698/18826/100038882/17886/16414/21894 | 6 | 305 |
| MF | GO:0004771 | sterol esterase activity | 2/130 | 18/28171 | 0.00308 | 0.017749 | 0.012507 | 628236/329055 | 2 | 18 |
| MF | GO:0005186 | pheromone activity | 2/130 | 18/28171 | 0.00308 | 0.017749 | 0.012507 | 381530/668200 | 2 | 18 |
| MF | GO:0004806 | triglyceride lipase activity | 2/130 | 19/28171 | 0.003432 | 0.019318 | 0.013612 | 18946/67717 | 2 | 19 |
| MF | GO:0120014 | phospholipid transfer activity | 2/130 | 22/28171 | 0.004595 | 0.025273 | 0.017809 | 18738/56356 | 2 | 22 |
| MF | GO:0005539 | glycosaminoglycan binding | 5/130 | 235/28171 | 0.004765 | 0.025624 | 0.018056 | 21946/17523/11905/13035/16019 | 5 | 235 |
| MF | GO:0019843 | rRNA binding | 3/130 | 76/28171 | 0.005281 | 0.027514 | 0.019388 | 269261/65019/76846 | 3 | 76 |
| MF | GO:0004175 | endopeptidase activity | 7/130 | 456/28171 | 0.005344 | 0.027514 | 0.019388 | 18716/13030/13033/317653/626834/13035/19152 | 7 | 456 |
| MF | GO:1901567 | fatty acid derivative binding | 2/130 | 24/28171 | 0.005457 | 0.027514 | 0.019388 | 18738/15481 | 2 | 24 |
| MF | GO:0004869 | cysteine-type endopeptidase inhibitor activity | 3/130 | 78/28171 | 0.005677 | 0.028038 | 0.019757 | 18054/209294/58214 | 3 | 78 |
| MF | GO:0055106 | ubiquitin-protein transferase regulator activity | 2/130 | 26/28171 | 0.006388 | 0.030916 | 0.021785 | 20115/65019 | 2 | 26 |
| MF | GO:0005543 | phospholipid binding | 7/130 | 480/28171 | 0.007009 | 0.033258 | 0.023436 | 18738/15481/11746/56356/11814/21894/16019 | 7 | 480 |
| MF | GO:0031210 | phosphatidylcholine binding | 2/130 | 28/28171 | 0.007385 | 0.034367 | 0.024217 | 18738/16019 | 2 | 28 |
| MF | GO:0004190 | aspartic-type endopeptidase activity | 2/130 | 31/28171 | 0.009003 | 0.041107 | 0.028966 | 18716/13033 | 2 | 31 |
| MF | GO:0070001 | aspartic-type peptidase activity | 2/130 | 32/28171 | 0.009574 | 0.042906 | 0.030234 | 18716/13033 | 2 | 32 |

Table S4. Detailed assembly of glycosylated S-protein of MHV.

| **Residue (chain)** | **Type** | **Sequence** | **Source** |
| --- | --- | --- | --- |
| **N31 (A)** | FA2 | bDGlcNAc(1→2)aDMan(1→6)[bDGlcNAc(1→2)aDMan(1→3)]bDMan(1→4)bDGlcNAc(1→4) [aLFuc(1→6)]bDGlcNAc(1→)PROA-31 | Watanabe et al. [13] |
| **N657 (A)** | A2 | bDGlcNAc(1→2)aDMan(1→6) [bDGlcNAc(1→2)aDMan(1→3)]bDMan(1→4)bDGlcNAc(1→4)bDGlcNAc(1→)PROA-657 | Watanabe et al. [13] |
| **N754 (A)** | Hybrid G1 | bDGal(1→4)bDGlcNAc(1→2)aDMan(1→3)[aDMan(1→6) [aDMan(1→3)]aDMan(1→6)]bDMan(1→4)bDGlcNAc(1→4)bDGlcNAc(1→)PROA-754 | Watanabe et al. [13] |
| **N844 (A)** | M6 | aDMan(1→6)[aDMan(1→3)]aDMan(1→6) [aDMan(1→2)aDMan(1→3)]bDMan(1→4)bDGlcNAc(1→4)bDGlcNAc(1→)PROA-844 | Watanabe et al. [13] |
| **N31 (B)** | FA3 | bDGlcNAc(1→6)[bDGlcNAc(1→2)]aDMan(1→6) [bDGlcNAc(1→2)aDMan(1→3)]bDMan(1→4)bDGlcNAc(1→4)[aLFuc(1→6)]bDGlcNAc(1→)PROB-31 | Watanabe et al. [13] |
| **N657 (B)** | FA2 | bDGlcNAc(1→2)aDMan(1→6)[bDGlcNAc(1→2)aDMan(1→3)]bDMan(1→4)bDGlcNAc(1→4) [aLFuc(1→6)]bDGlcNAc(1→)PROB-657 | Watanabe et al. [13] |
| **N754 (B)** | M5 | aDMan(1→6)[aDMan(1→3)]aDMan(1→6) [aDMan(1→3)]bDMan(1→4)bDGlcNAc(1→4)bDGlcNAc(1→)PROB-754 | Watanabe et al. [13] |
| **N844 (B)** | M7 | aDMan(1→2)aDMan(1→2)aDMan(1→3)[aDMan(1→6) [aDMan(1→3)]aDMan(1→6)]bDMan(1→4)bDGlcNAc(1→4)bDGlcNAc(1→)PROB-844 | Watanabe et al. [13] |
| **N31 (C)** | FA3 | bDGlcNAc(1→6)[bDGlcNAc(1→2)]aDMan(1→6) [bDGlcNAc(1→2)aDMan(1→3)]bDMan(1→4)bDGlcNAc(1→4)[aLFuc(1→6)]bDGlcNAc(1→)PROC-31 | Watanabe et al. [13] |
| **N657 (C)** | FA2 | bDGlcNAc(1→2)aDMan(1→6)[bDGlcNAc(1→2)aDMan(1→3)]bDMan(1→4)bDGlcNAc(1→4) [aLFuc(1→6)]bDGlcNAc(1→)PROC-657 | Watanabe et al. [13] |
| **N754 (C)** | M6 | aDMan(1→6)[aDMan(1→3)]aDMan(1→6) [aDMan(1→2)aDMan(1→3)]bDMan(1→4)bDGlcNAc(1→4)bDGlcNAc(1→)PROC-754 | Watanabe et al. [13] |
| **N844 (C)** | M5 | aDMan(1→6)[aDMan(1→3)]aDMan(1→6) [aDMan(1→3)]bDMan(1→4)bDGlcNAc(1→4)bDGlcNAc(1→)PROC-844 | Watanabe et al. [13] |
| **N192 (A)** | M5 | aDMan(1→6)[aDMan(1→3)]aDMan(1→6) [aDMan(1→3)]bDMan(1→4)bDGlcNAc(1→4)bDGlcNAc(1→)PROA-192 | 6VSJ [8] |
| **N192 (B)** | M5 | aDMan(1→6)[aDMan(1→3)]aDMan(1→6) [aDMan(1→3)]bDMan(1→4)bDGlcNAc(1→4)bDGlcNAc(1→)PROB-192 | 6VSJ [8] |
| **N192 (C)** | M5 | aDMan(1→6)[aDMan(1→3)]aDMan(1→6) [aDMan(1→3)]bDMan(1→4)bDGlcNAc(1→4)bDGlcNAc(1→)PROC-192 | 6VSJ [8] |
| **N435 (A)** | M6 | aDMan(1→6)[aDMan(1→3)]aDMan(1→6) [aDMan(1→2)aDMan(1→3)]bDMan(1→4)bDGlcNAc(1→4)bDGlcNAc(1→)PROA-435 | 6VSJ [8] |
| **N435 (B)** | M5 | aDMan(1→6)[aDMan(1→3)]aDMan(1→6) [aDMan(1→3)]bDMan(1→4)bDGlcNAc(1→4)bDGlcNAc(1→)PROB-435 | 6VSJ [8] |
| **N435 (C)** | M7 | aDMan(1→2)aDMan(1→2)aDMan(1→3)[aDMan(1→6) [aDMan(1→3)]aDMan(1→6)]bDMan(1→4)bDGlcNAc(1→4)bDGlcNAc(1→)PROC-435 | 6VSJ [8] |
| **N357 (A)** | M5 | aDMan(1→6)[aDMan(1→3)]aDMan(1→6) [aDMan(1→3)]bDMan(1→4)bDGlcNAc(1→4)bDGlcNAc(1→)PROA-357 | NXS motif |
| **N357 (B)** | M6 | aDMan(1→6)[aDMan(1→3)]aDMan(1→6) [aDMan(1→2)aDMan(1→3)]bDMan(1→4)bDGlcNAc(1→4)bDGlcNAc(1→)PROB-357 | NXS motif |
| **N357 (C)** | M5 | aDMan(1→6)[aDMan(1→3)]aDMan(1→6) [aDMan(1→3)]bDMan(1→4)bDGlcNAc(1→4)bDGlcNAc(1→)PROC-357 | NXS motif |
| **N530 (A)** | M7 | aDMan(1→2)aDMan(1→2)aDMan(1→3)[aDMan(1→6) [aDMan(1→3)]aDMan(1→6)]bDMan(1→4)bDGlcNAc(1→4)bDGlcNAc(1→)PROA-530 | NXS motif |
| **N530 (B)** | M6 | aDMan(1→6)[aDMan(1→3)]aDMan(1→6) [aDMan(1→2)aDMan(1→3)]bDMan(1→4)bDGlcNAc(1→4)bDGlcNAc(1→)PROB-530 | NXS motif |
| **N530 (C)** | M5 | aDMan(1→6)[aDMan(1→3)]aDMan(1→6) [aDMan(1→3)]bDMan(1→4)bDGlcNAc(1→4)bDGlcNAc(1→)PROC-530 | NXS motif |
| **N665 (A)** | M5 | aDMan(1→6)[aDMan(1→3)]aDMan(1→6) [aDMan(1→3)]bDMan(1→4)bDGlcNAc(1→4)bDGlcNAc(1→)PROA-665 | NXS motif |
| **N665 (B)** | M7 | aDMan(1→2)aDMan(1→2)aDMan(1→3)[aDMan(1→6) [aDMan(1→3)]aDMan(1→6)]bDMan(1→4)bDGlcNAc(1→4)bDGlcNAc(1→)PROB-665 | NXS motif |
| **N665 (C)** | M5 | aDMan(1→6)[aDMan(1→3)]aDMan(1→6) [aDMan(1→3)]bDMan(1→4)bDGlcNAc(1→4)bDGlcNAc(1→)PROC-665 | NXS motif |
| **N737 (A)** | M5 | aDMan(1→6)[aDMan(1→3)]aDMan(1→6) [aDMan(1→3)]bDMan(1→4)bDGlcNAc(1→4)bDGlcNAc(1→)PROA-737 | NXS motif |
| **N737 (B)** | M5 | aDMan(1→6)[aDMan(1→3)]aDMan(1→6) [aDMan(1→3)]bDMan(1→4)bDGlcNAc(1→4)bDGlcNAc(1→)PROB-737 | NXS motif |
| **N737 (C)** | M6 | aDMan(1→6)[aDMan(1→3)]aDMan(1→6) [aDMan(1→2)aDMan(1→3)]bDMan(1→4)bDGlcNAc(1→4)bDGlcNAc(1→)PROC-737 | NXS motif |
| **N1126 (A)** | M7 | aDMan(1→2)aDMan(1→2)aDMan(1→3)[aDMan(1→6) [aDMan(1→3)]aDMan(1→6)]bDMan(1→4)bDGlcNAc(1→4)bDGlcNAc(1→)PROA-1126 | NXS motif |
| **N1126 (B)** | M5 | aDMan(1→6)[aDMan(1→3)]aDMan(1→6) [aDMan(1→3)]bDMan(1→4)bDGlcNAc(1→4)bDGlcNAc(1→)PROB-1126 | NXS motif |
| **N1126 (C)** | M5 | aDMan(1→6)[aDMan(1→3)]aDMan(1→6) [aDMan(1→3)]bDMan(1→4)bDGlcNAc(1→4)bDGlcNAc(1→)PROC-1126 | NXS motif |
| **N60 (A)** | M6 | aDMan(1→6)[aDMan(1→3)]aDMan(1→6) [aDMan(1→2)aDMan(1→3)]bDMan(1→4)bDGlcNAc(1→4)bDGlcNAc(1→)PROA-60 | NXT motif |
| **N60 (B)** | M6 | aDMan(1→6)[aDMan(1→3)]aDMan(1→6) [aDMan(1→2)aDMan(1→3)]bDMan(1→4)bDGlcNAc(1→4)bDGlcNAc(1→)PROB-60 | NXT motif |
| **N60 (C)** | M5 | aDMan(1→6)[aDMan(1→3)]aDMan(1→6) [aDMan(1→3)]bDMan(1→4)bDGlcNAc(1→4)bDGlcNAc(1→)PROC-60 | NXT motif |
| **N625 (A)** | M5 | aDMan(1→6)[aDMan(1→3)]aDMan(1→6) [aDMan(1→3)]bDMan(1→4)bDGlcNAc(1→4)bDGlcNAc(1→)PROA-625 | NXT motif |
| **N625 (B)** | M7 | aDMan(1→2)aDMan(1→2)aDMan(1→3)[aDMan(1→6) [aDMan(1→3)]aDMan(1→6)]bDMan(1→4)bDGlcNAc(1→4)bDGlcNAc(1→)PROB-625 | NXT motif |
| **N625 (C)** | M5 | aDMan(1→6)[aDMan(1→3)]aDMan(1→6) [aDMan(1→3)]bDMan(1→4)bDGlcNAc(1→4)bDGlcNAc(1→)PROC-625 | NXT motif |
| **N688 (A)** | M6 | aDMan(1→6)[aDMan(1→3)]aDMan(1→6) [aDMan(1→2)aDMan(1→3)]bDMan(1→4)bDGlcNAc(1→4)bDGlcNAc(1→)PROA-688 | NXT motif |
| **N688 (B)** | M5 | aDMan(1→6)[aDMan(1→3)]aDMan(1→6) [aDMan(1→3)]bDMan(1→4)bDGlcNAc(1→4)bDGlcNAc(1→)PROB-688 | NXT motif |
| **N688 (C)** | M7 | aDMan(1→2)aDMan(1→2)aDMan(1→3)[aDMan(1→6) [aDMan(1→3)]aDMan(1→6)]bDMan(1→4)bDGlcNAc(1→4)bDGlcNAc(1→)PROC-688 | NXT motif |
| **N893 (A)** | M6 | aDMan(1→6)[aDMan(1→3)]aDMan(1→6) [aDMan(1→2)aDMan(1→3)]bDMan(1→4)bDGlcNAc(1→4)bDGlcNAc(1→)PROA-893 | NXT motif |
| **N893 (B)** | M5 | aDMan(1→6)[aDMan(1→3)]aDMan(1→6) [aDMan(1→3)]bDMan(1→4)bDGlcNAc(1→4)bDGlcNAc(1→)PROB-893 | NXT motif |
| **N893 (C)** | M7 | aDMan(1→2)aDMan(1→2)aDMan(1→3)[aDMan(1→6) [aDMan(1→3)]aDMan(1→6)]bDMan(1→4)bDGlcNAc(1→4)bDGlcNAc(1→)PROC-893 | NXT motif |

1. SUPPLEMENTARY INFORMATION REFERENCES

[1] Arévalo AP, Pagotto R, Pórfido JL, Daghero H, Segovia M, Yamasaki K, et al. Ivermectin reduces in vivo coronavirus infection in a mouse experimental model. Sci Rep 2021;11. https://doi.org/10.1038/S41598-021-86679-0.

[2] Kyuwa S, Shibata S, Tagawa YI, Iwakura Y, Machii K, Urano T. Acute hepatic failure in IFN-γ-deficient BALB/c mice after murine coronavirus infection. Virus Res 2002;83:169–77. https://doi.org/10.1016/S0168-1702(01)00432-4.

[3] Carvalho PC, Lima DB, Leprevost F V., Santos MDM, Fischer JSG, Aquino PF, et al. Integrated analysis of shotgun proteomic data with PatternLab for proteomics 4.0. Nat Protoc 2015 111 2015;11:102–17. https://doi.org/10.1038/nprot.2015.133.

[4] Santos MDM, Lima DB, Fischer JSG, Clasen MA, Kurt LU, Camillo-Andrade AC, et al. Simple, efficient and thorough shotgun proteomic analysis with PatternLab V. Nat Protoc 2022 177 2022;17:1553–78. https://doi.org/10.1038/s41596-022-00690-x.

[5] Wu T, Hu E, Xu S, Chen M, Guo P, Dai Z, et al. clusterProfiler 4.0: A universal enrichment tool for interpreting omics data. Innov 2021;2:100141. https://doi.org/10.1016/J.XINN.2021.100141.

[6] Rivera B, Leyva A, Portela MM, Moratorio G, Moreno P, Durán R, et al. Quantitative proteomic dataset from oro- and naso-pharyngeal swabs used for COVID-19 diagnosis: Detection of viral proteins and host’s biological processes altered by the infection. Data Br 2020;32. https://doi.org/10.1016/J.DIB.2020.106121.

[7] Perez-Riverol Y, Bai J, Bandla C, García-Seisdedos D, Hewapathirana S, Kamatchinathan S, et al. The PRIDE database resources in 2022: a hub for mass spectrometry-based proteomics evidences. Nucleic Acids Res 2022;50:D543–52. https://doi.org/10.1093/NAR/GKAB1038.

[8] Shang J, Wan Y, Liu C, Yount B, Gully K, Yang Y, et al. Structure of mouse coronavirus spike protein complexed with receptor reveals mechanism for viral entry. PLoS Pathog 2020;16. https://doi.org/10.1371/JOURNAL.PPAT.1008392.

[9] Molecular Operating Environment (MOE) n.d.

[10] Rosa A, Pye VE, Graham C, Muir L, Seow J, Ng KW, et al. SARS-CoV-2 can recruit a heme metabolite to evade antibody immunity. Sci Adv 2021;7. https://doi.org/10.1126/sciadv.abg7607.

[11] Shapiro A, Botha JD, Pastore A, Lesk AM. A method for multiple superposition of structures. Acta Crystallogr A 1992;48 ( Pt 1):11–4. https://doi.org/10.1107/S010876739100867X.

[12] Case DA, Berryman JT, Betz RM, Cerutti DS, Cheatham III TE, Darden TA, et al. AMBER 2015. Univ California, San Fr 2015.

[13] Watanabe Y, Allen JD, Wrapp D, McLellan JS, Crispin M. Site-specific glycan analysis of the SARS-CoV-2 spike. Science 2020;369:330–3. https://doi.org/10.1126/SCIENCE.ABB9983.

[14] Watanabe Y, Berndsen ZT, Raghwani J, Seabright GE, Allen JD, Pybus OG, et al. Vulnerabilities in coronavirus glycan shields despite extensive glycosylation. Nat Commun 2020;11. https://doi.org/10.1038/S41467-020-16567-0.

[15] Shajahan A, Supekar NT, Gleinich AS, Azadi P. Deducing the N- and O-glycosylation profile of the spike protein of novel coronavirus SARS-CoV-2. Glycobiology 2020;30:981. https://doi.org/10.1093/GLYCOB/CWAA042.

[16] Walls AC, Park YJ, Tortorici MA, Wall A, McGuire AT, Veesler D. Structure, Function, and Antigenicity of the SARS-CoV-2 Spike Glycoprotein. Cell 2020;181:281-292.e6. https://doi.org/10.1016/J.CELL.2020.02.058.

[17] Casalino L, Gaieb Z, Goldsmith JA, Hjorth CK, Dommer AC, Harbison AM, et al. Beyond Shielding: The Roles of Glycans in SARS-CoV-2 Spike Protein. BioRxiv 2020. https://doi.org/10.1101/2020.06.11.146522.

[18] Zheng J, Yamada Y, Fung TS, Huang M, Chia R, Liu DX. Identification of N-linked glycosylation sites in the spike protein and their functional impact on the replication and infectivity of coronavirus infectious bronchitis virus in cell culture. Virology 2018;513:65. https://doi.org/10.1016/J.VIROL.2017.10.003.

[19] Ritchie G, Harvey DJ, Feldmann F, Stroeher U, Feldmann H, Royle L, et al. Identification of N-linked carbohydrates from severe acute respiratory syndrome (SARS) spike glycoprotein. Virology 2010;399:257–69. https://doi.org/10.1016/J.VIROL.2009.12.020.

[20] Park SJ, Lee J, Qi Y, Kern NR, Lee HS, Jo S, et al. CHARMM-GUI Glycan Modeler for modeling and simulation of carbohydrates and glycoconjugates. Glycobiology 2019;29:320–31. https://doi.org/10.1093/GLYCOB/CWZ003.

[21] Jo S, Kim T, Iyer VG, Im W. CHARMM-GUI: A web-based graphical user interface for CHARMM. J Comput Chem 2008;29:1859–65. https://doi.org/10.1002/JCC.20945.

[22] Abraham MJ, Murtola T, Schulz R, Páll S, Smith JC, Hess B, et al. GROMACS: High performance molecular simulations through multi-level parallelism from laptops to supercomputers. SoftX 2015;1:19–25. https://doi.org/10.1016/J.SOFTX.2015.06.001.

[23] Nosé S. A unified formulation of the constant temperature molecular dynamics methods. J Chem Phys 1984;81:511–9. https://doi.org/10.1063/1.447334.

[24] Berendsen HJC, Postma JPM, Van Gunsteren WF, Dinola A, Haak JR. Molecular dynamics with coupling to an external bath. J Chem Phys 1984;81:3684–90. https://doi.org/10.1063/1.448118.

[25] Bussi G, Donadio D, Parrinello M. Canonical sampling through velocity rescaling. J Chem Phys 2007;126. https://doi.org/10.1063/1.2408420/186581.

[26] Parrinello M, Rahman A. Polymorphic transitions in single crystals: A new molecular dynamics method. J Appl Phys; (United States) 1981;52:12:7182–90. https://doi.org/10.1063/1.328693.

[27] Ewald PP. Die Berechnung optischer und elektrostatischer Gitterpotentiale. Ann Phys 1921;369:253–87. https://doi.org/10.1002/ANDP.19213690304.

[28] Bekker H. LINCS: A linear constraint solver for molecular simulations. J Comput Chem 1997.

[29] Gowers RJ, Linke M, Barnoud J, E Reddy TJ, Melo MN, Seyler SL, et al. MDAnalysis: A Python Package for the Rapid Analysis of Molecular Dynamics Simulations. PROC 15th PYTHON Sci CONF 2016.

[30] Humphrey W, Dalke A, Schulten K. VMD: visual molecular dynamics. J Mol Graph 1996;14:33–8, 27. https://doi.org/10.1016/0263-7855(96)00018-5.

[31] Rammauro F, Carrión F, Olivero-Deibe N, Fló M, Ferreira A, Pritsch O, et al. Humoral immune response characterization of heterologous prime-boost vaccination with CoronaVac and BNT162b2. Vaccine 2022;40:5189–96. https://doi.org/10.1016/J.VACCINE.2022.07.023.

[32] Freeman SL, Oliveira ASF, Gallio AE, Rosa A, Simitakou MK, Arthur CJ, et al. Heme binding to the SARS-CoV-2 spike glycoprotein. J Biol Chem 2023. https://doi.org/10.1016/j.jbc.2023.105014.
